# Comparative effectiveness of non-surgical interventions and their combinations for the prevention of breast cancer-related lymphedema: a systematic review and Bayesian network meta-analysis

**DOI:** 10.3389/fonc.2026.1940372

**Published:** 2026-09-21

**Authors:** Naihao Zhu, Jinxia Chang, Gang Cao, Rufeng Teng, Jinbang Wang, Wenyu Bu, Yanhong Peng, Jianchun Cao

**Affiliations:** 1 Beijing University of Chinese Medicine Dongfang Hospital, Beijing, China; 2 Tianjin University of Traditional Chinese Medicine, Tianjin, China; 3 Tianjin Academy of Traditional Chinese Medicine Affiliated Hospital, Tianjin, China

**Keywords:** Bayesian network meta-analysis, breast cancer-related lymphedema, compression therapy, intermittent pneumatic compression, manual lymphatic drainage, prevention, resistance training, shoulder range of motion

## Abstract

**Objective:**

The aims of this study were to compare the relative effectiveness of non-surgical preventive interventions and their combinations on breast cancer-related lymphedema (BCRL) incidence and shoulder abduction range of motion (ROM) using Bayesian network meta-analysis (NMA) and to assess the certainty of evidence using the Confidence in Network Meta-Analysis (CINeMA) framework.

**Methods:**

Eight databases (PubMed, Embase, Web of Science, Cochrane Library, CNKI, WanFang, VIP, and SinoMed) were searched through January 2026. Randomized controlled trials (RCTs) of non-surgical interventions for BCRL prevention in post-surgical breast cancer patients were included. The primary outcome was BCRL incidence, which was analyzed using a Bayesian random-effects NMA with arm-level binomial likelihood and log link (risk ratio). The primary analysis was restricted to studies with a follow-up of at least 6 months, based on evidence that the risk of BCRL in axillary lymph node dissection populations peaks at 6–12 months postoperatively. A meta-regression with follow-up duration as a covariate was conducted on the full 45-study network to validate this restriction. Shoulder abduction ROM was analyzed as a secondary outcome after data cleaning to ensure measurement homogeneity.

**Results:**

A total of 100 RCTs were included in the qualitative synthesis. The primary BCRL analysis comprised 27 studies (57 arms, 4,660 participants, 11 nodes). Seven interventions showed credible intervals excluding 1: compression therapy combined with resistance training (COMP+RT) [risk ratio (RR) = 0.146, 95% credible interval (95%CrI) = 0.057–0.366, surface under the cumulative ranking curve (SUCRA) = 85.1%], RT with supervised exercise (RT+SUP-EX) (RR = 0.147, 95%CrI = 0.019–0.665, SUCRA = 80.8%), COMP with manual lymphatic drainage (COMP+MLD) (RR = 0.121, 95%CrI = 0.004–0.915, SUCRA = 80.6%), intermittent pneumatic compression with MLD (IPC+MLD) (RR = 0.255, 95%CrI = 0.153–0.397, SUCRA = 69.3%), microwave therapy (MW) (RR = 0.313, 95%CrI = 0.090–0.926, SUCRA = 57.8%), IPC (RR = 0.396, 95%CrI = 0.197–0.765, SUCRA = 47.8%), and MLD (RR = 0.467, 95%CrI = 0.320–0.650, SUCRA = 39.2%). COMP+RT ranked first in three of six analytical approaches. However, it was excluded as a sparse node under stricter diagnostic definitions. The meta-regression coefficient for follow-up duration was statistically significant (*β* = 0.077/month, 95%CrI = 0.026–0.128), confirming that the treatment effects attenuated with longer follow-up. The cleaned ROM analysis (12 studies, 1,079 participants, 11 nodes) identified eight interventions with credible intervals excluding zero, led by acupoint massage with isokinetic strength training (AM+ISO) [mean difference (MD) = 22.83°, 95%CrI = 11.06–34.89, SUCRA = 85.2%]. Two interventions ranked in the top four for both outcomes: COMP+RT (BCRL rank 1 and ROM rank 4) and IPC+MLD (BCRL rank 4 and ROM rank 3).

**Conclusion:**

COMP+RT demonstrated the most robust evidence profile among all evaluated interventions, ranking first for BCRL incidence in three of six analytical approaches and in the top four for shoulder ROM. However, its evidence base was concentrated among studies using author-defined diagnostic criteria; under stricter diagnostic definitions, COMP+RT was excluded as a sparse node. These findings should be interpreted in the context of a predominantly low certainty evidence. Confirmatory trials with standardized diagnostic criteria and follow-up of at least 12 months are needed.

**Systematic review registration:**

https://www.crd.york.ac.uk/PROSPERO/, identifier CRD420261279759.

## 1 Introduction

Breast cancer-related lymphedema (BCRL) is one of the most common long-term complications of breast cancer treatment, manifesting as persistent swelling of the ipsilateral upper limb accompanied by heaviness, pain, restricted shoulder mobility, and reduced grip strength. The underlying pathophysiology involves surgical and radiation-induced disruption of the axillary lymphatic drainage pathways, leading to interstitial fluid accumulation, chronic inflammation, adipose deposition, and tissue fibrosis. Once BCRL progresses beyond the early reversible stage, it typically requires lifelong management. Prevention is therefore a clinical priority that should be integrated into postoperative rehabilitation and survivorship care.

The incidence of BCRL is substantial and time-dependent. DiSipio et al. (1) estimated an overall incidence of 21.4% among breast cancer survivors. However, the rates vary with surgical extent, radiotherapy exposure, diagnostic criteria, and follow-up duration (15). McDuff et al. (2), in a prospective cohort of 2,171 women, demonstrated that BCRL risk peaks at 6–12 months in patients undergoing axillary lymph node dissection (ALND) without regional radiation, at 18–24 months in those receiving ALND with radiation, and at 36–48 months following sentinel lymph node biopsy with radiation. This time-dependent epidemiology has direct implications for how prevention trials should be synthesized: studies measuring BCRL at 3 months capture a fundamentally different risk phase than those measuring at 12 months, and pooling them without adjustment may compromise the comparative effectiveness estimates.

A diverse array of non-surgical interventions has been proposed for BCRL prevention. These include manual lymphatic drainage (MLD), compression therapy (COMP), intermittent pneumatic compression (IPC), resistance training (RT), supervised exercise (SUP-EX), acupoint massage (AM), moxibustion (Mox), and various combinations. Each modality targets a different segment of the pathophysiological cascade: COMP and IPC address fluid accumulation and venous–lymphatic return, MLD promotes superficial lymphatic transport, RT and SUP-EX enhance the skeletal muscle pump and shoulder girdle function, while AM and Mox have been explored as adjunctive modalities in integrative rehabilitation programs.

The evidence base for BCRL prevention remains fragmented. Existing systematic reviews have focused on single-intervention categories: Hayes et al. (3) evaluated exercise, Su et al. (5) examined IPC, and Rafn et al. (4) assessed prospective surveillance-triggered intervention. Each made important contributions, but none could answer the question that matters most to practitioners and patients: among all available preventive strategies, which offers the greatest protection? Traditional pairwise meta-analysis cannot address this question because it compares only two interventions at a time. The Multinational Association of Supportive Care in Cancer (MASCC) clinical practice guidance (6) provides consensus-based recommendations, but relies on expert opinion rather than quantitative comparative effectiveness data.

To address this gap, we conducted a Bayesian network meta-analysis (NMA) incorporating all major categories of non-surgical preventive interventions and their combinations. Three design features distinguish this study. First, the primary analysis of BCRL incidence was restricted to studies with at least 6 months of follow-up, based on the clinical epidemiology of BCRL onset in ALND populations, with a network meta-regression validating this restriction. Second, shoulder abduction range of motion (ROM) was analyzed as a secondary outcome after systematic data cleaning, capturing the early rehabilitation window. Third, evidence certainty was assessed using the Confidence in Network Meta-Analysis (CINeMA) framework, and a prespecified three-dimensional interpretation framework integrating surface under the cumulative ranking curve (SUCRA) stability, CINeMA certainty, and cross-outcome consistency was applied to generate clinically actionable recommendations.

## 2 Methods

### 2.1 Registration, reporting standards, and study design

This study is a systematic review and Bayesian NMA of randomized controlled trials (RCTs) evaluating non-surgical interventions for the prevention of BCRL. The study was reported in accordance with the Preferred Reporting Items for Systematic Reviews and Meta-analyses (PRISMA) 2020 statement (7), the PRISMA extension for network meta-analyses (8), and the Cochrane Handbook recommendations for undertaking NMA (11). The protocol was prospectively registered with PROSPERO (CRD420261279759). The scope was limited to preventive interventions initiated after breast cancer surgery, and interventions for established clinical lymphedema were excluded.

### 2.2 Search strategy

Eight electronic databases were systematically searched: PubMed, Embase, Web of Science, Cochrane Library, China National Knowledge Infrastructure (CNKI), WanFang Data, VIP Database, and SinoMed. All searches were conducted between 20 and 24 January 2026, with no language restrictions. The reference lists of all included studies and relevant systematic reviews were screened for additional eligible records. The search strategy combined terms across four modules: disease (breast cancer, breast neoplasms), outcome (lymphedema), intervention (manual lymphatic drainage, compression, exercise, resistance training, intermittent pneumatic compression, acupoint massage, moxibustion), and study design (randomized). Database-specific adaptations of subject headings, free-text terms, and Boolean operators were applied. The full search strategies are provided in **Supplementary Material 1**, Section S1 reported following the PRISMA-S guidelines (17).

### 2.3 Eligibility criteria

Studies were eligible according to the following PICOS (population, intervention, comparison, outcome, and study design) criteria. Participants were adult women who had undergone breast cancer surgery and were at risk of BCRL, but had not developed established clinical lymphedema at baseline. No restrictions were placed on surgical type, radiotherapy, or systemic therapy.

Interventions were non-surgical measures aimed at preventing postoperative BCRL, including but not limited to MLD, IPC, COMP, RT, SUP-EX, AM, Mox, isokinetic strength training (ISO), and their combinations. Comparators included usual care, health education, limb protection advice, unsupervised basic functional exercise, no additional active intervention, or other active preventive measures.

The primary outcome was BCRL incidence. Secondary outcomes included shoulder abduction ROM and upper limb volume difference. Only RCTs with two or more arms were eligible. Conference abstracts without full-text availability, non-randomized studies, cohort studies, case series, reviews, animal experiments, and duplicate publications were excluded. For studies using ambiguous terms such as “treatment” or “prevention and treatment,” inclusion required confirmation that participants had not developed established lymphedema before randomization and that the stated intervention purpose was prevention or early risk control.

### 2.4 Study selection and data extraction

Search records were imported into EndNote for deduplication. Two reviewers independently screened the titles and abstracts, followed by full-text assessment, with disagreements resolved through discussion or adjudication by a third reviewer. Data were extracted using a predesigned form covering author, publication year, country, sample size, surgical and treatment background, original intervention description, standardized intervention node, BCRL diagnostic method and threshold, follow-up time point, event counts and sample sizes for binary outcomes, and the means, standard deviations, and sample sizes for continuous outcomes. All extracted data were independently verified by two reviewers.

### 2.5 Intervention node standardization

A component-based node standardization strategy was adopted. Usual care, health education, limb protection, unsupervised basic functional exercise, and routine rehabilitation guidance were unified into the reference node UC-EX (usual care/education with unsupervised exercise). When both arms in a study received identical background care, the nodes were coded based solely on the additional active preventive components. Combination interventions were coded by their active components (e.g., IPC+MLD or COMP+RT). The complete node coding scheme and definitions are provided in **Supplementary Table 1**, Section S3.

### 2.6 Outcome definitions and data handling

#### 2.6.1 Primary outcome: BCRL incidence

BCRL incidence was a binary outcome. When a study reported multiple BCRL definitions, a predefined priority hierarchy was applied: volumetric binary incidence (definition class 1A) was preferred over circumferential binary incidence (1B), which was preferred over unclear or author-defined thresholds (1D).

The primary analysis was restricted to studies with follow-up of at least 6 months. McDuff et al. (2) demonstrated that the risk of BCRL in ALND populations peaks at 6–12 months postoperatively. Data collected before this window capture a period of systematically lower event rates, and pooling such data with studies with longer follow-up violates the transitivity assumption underlying NMA. This time restriction is applicable to the present network because 38 of the 40 studies with available surgical data (95%) enrolled exclusively ALND patients. Studies with follow-up of less than 6 months were retained in a meta-regression sensitivity analysis.

The event counts and sample sizes were extracted for each study arm. Single-zero-event studies were retained in the arm-level binomial likelihood model without continuity correction. Double-zero-event studies were excluded from the risk ratio (RR) model.

#### 2.6.2 Secondary outcome: shoulder abduction ROM

Shoulder abduction ROM was a continuous outcome measured in degrees, with higher values indicating better function [mean difference (MD) > 0 favoring the intervention]. A data cleaning protocol was applied prior to the analysis to ensure measurement homogeneity. Studies were excluded if they reported change from baseline scores rather than post-treatment absolute values, measured ROM at a time point reflecting acute surgical restriction rather than recovery trajectory, or reported absolute values clinically incomparable with the majority of the included studies. After exclusion of five studies on these grounds, one additional study (study 041: AM+IPC *vs*. AM) became disconnected from the reference node UC-EX and was excluded along with its two nodes, yielding 12 studies with 11 nodes.

#### 2.6.3 Secondary outcome: upper limb volume difference

Upper limb volume difference was originally planned as a continuous NMA outcome. Data auditing identified two irreconcilable problems: studies mixed fundamentally different constructs (post-treatment inter-limb difference *vs*. change from baseline in volume), and 5 of 12 studies reported implausibly narrow standard deviations relative to their means (SD/mean ratio < 0.10). NMA was abandoned for this outcome, and directional findings are presented as a descriptive narrative synthesis.

### 2.7 Transitivity assessment

The transitivity assumption was assessed across four potential effect modifiers.

1. Follow-up duration: Because BCRL incidence is cumulative and hazard varies with time, studies measuring outcomes at different time points may have systematically different baseline event rates. This was addressed through the ≥6-month time restriction and meta-regression (*Section 2.11.1*).
2. Axillary surgical extent: Among the 40 studies with available data, 38 (95%) enrolled exclusively ALND patients, indicating high population homogeneity.
3. The BCRL diagnostic criteria varied across studies (volumetric 1A, circumferential 1B, and unclear 1D), addressed through diagnostic-class sensitivity analyses. Radiotherapy exposure could not be assessed as a transitivity variable because 64% of the studies did not report radiotherapy rates.
4. Baseline risk—the event risk in the reference (UC-EX) arm—was considered as a potential effect modifier, both because it summarizes unmeasured patient-level determinants of BCRL risk and because relative effect measures are not invariant to it. Variations in baseline risk were addressed using three features of the analysis: the ≥6-month restriction substantially reduces variation from the largest single determinant of cumulative incidence; the network meta-regression on follow-up duration (*Section 2.11.1*) directly adjusts for this determinant; and the diagnostic-class sensitivity analyses (*Section 2.11.2*) address the fact that the diagnostic threshold determines what is counted as an event and therefore sets the observed baseline risk. The distribution of the control arm event rates across the primary analysis studies is reported in *Section 3.3.1*.

### 2.8 Risk of bias assessment

Methodological quality was assessed using the Cochrane Risk of Bias 2 (RoB 2) tool (9) across five domains: randomization process, deviations from intended interventions, missing outcome data, measurement of the outcome, and selection of the reported result. Two reviewers assessed each study independently, with disagreements resolved through discussion or adjudication by a third reviewer.

### 2.9 Certainty of network evidence

The certainty of evidence for each network comparison was evaluated using the CINeMA framework (10) across six domains: within-study bias, reporting bias, indirectness, imprecision, heterogeneity, and incoherence. Reporting bias was informed by comparison-adjusted funnel plots and the Egger regression test. Imprecision was judged against prespecified clinical equivalence margins: RR = 0.80–1.25 for BCRL incidence and ±10° for shoulder abduction ROM. Heterogeneity was assessed using the posterior distribution of the between-study standard deviation (*τ*). Incoherence was evaluated using global consistency testing and local node-splitting analysis.

### 2.10 Statistical analysis

#### 2.10.1 Model specification

Bayesian random-effects NMA was used to estimate the relative treatment effects. For BCRL incidence, an arm-level binomial likelihood with log link function was specified (13), yielding treatment effects as log RRs. The network reference node was UC-EX; RR < 1 indicates reduced BCRL risk. RR was selected over odds ratio (OR) because it provides a more clinically interpretable effect measure for preventive interventions, and the observed control arm BCRL incidence in the primary analysis (pooled = 22.3%, median across studies = 25.0%) lies in a range where the non-collapsibility of the OR can overstate the effect magnitudes. A sensitivity analysis using ORs (logit link) was conducted to verify that this choice did not influence the conclusions.

Treatment effect priors were set as d_k_ ~ Normal(0, 2.5^2^), and the between-study heterogeneity parameter was assigned *τ* ~ HalfNormal(0, 1^2^). For shoulder abduction ROM, a normal likelihood with identity link was specified, with treatment effects as MDs in degrees (MD > 0 favoring the intervention). Treatment effect priors were d_k_ ~ Normal(0, 100^2^) and *τ* ~ HalfNormal(0, 50^2^). Within-study correlations for multi-arm studies were preserved in both models.

#### 2.10.2 MCMC sampling and convergence

All models were fitted using the gemtc package (version 0.8-4) (12) in R, calling JAGS for Markov chain Monte Carlo sampling. Four chains were run, each with 10,000 adaptation iterations, 50,000 burn-in iterations, and 100,000 posterior sampling iterations (thinning interval = 10). Convergence was assessed using the Gelman–Rubin statistic (Rhat < 1.05, target ≤ 1.01), trace plots, posterior density plots, autocorrelation plots, and effective sample size (ESS > 1,000).

#### 2.10.3 Model fit and ranking

Model fit was evaluated using the deviance information criterion (DIC). The intervention rankings were computed from posterior ranking probabilities and the SUCRA. The SUCRA values were interpreted as summaries of ranking probability, not as measures of evidence certainty or clinical recommendation strength.

### 2.11 Sensitivity analyses and consistency assessment

#### 2.11.1 Network meta-regression

To evaluate whether treatment effects were modified by follow-up duration, a Bayesian network meta-regression was conducted on all 45 sparse-removed studies, with follow-up time (months) as a study-level covariate. A shared coefficient treatment–covariate interaction model was specified, with the covariate centered and standardized following Gelman et al. (14). This analysis served two purposes: to estimate the magnitude of the time–treatment interaction and to verify whether the SUCRA rankings from the time-restricted primary analysis are consistent with those obtained after statistical adjustment for follow-up time.

#### 2.11.2 Data-level sensitivity analyses

Additional prespecified sensitivity analyses included the following: restriction to studies with volumetric diagnosis only (class 1A); exclusion of studies with unclear diagnostic thresholds (retaining 1A and 1B); restriction to non-Chinese-language publications; and use of OR as an alternative effect measure. For each subset, network connectivity was rechecked and treatment nodes appearing in only one study were removed before model fitting, consistent with the sparse node exclusion applied to the primary analysis.

#### 2.11.3 Prior sensitivity

For BCRL, treatment effect priors were varied to Normal(0, 5^2^) and Normal(0, 1.5^2^), while heterogeneity priors were varied to HalfNormal(0, 0.5^2^), HalfNormal(0, 2^2^), and Uniform(0, 2). For ROM, treatment effect priors were varied to Normal(0, 200^2^) and Normal(0, 50^2^), and heterogeneity priors were varied to HalfNormal(0, 100^2^) and HalfNormal(0, 25^2^).

#### 2.11.4 Consistency assessment

Global consistency was evaluated by comparing the DIC between the consistency model and an unrelated mean effects (UME) model. Local consistency was assessed using node-splitting analysis on all comparisons with separable direct and indirect evidence.

### 2.12 Integrated interpretation framework

An intervention was considered a candidate for clinical recommendation when it satisfied at least two of three prespecified criteria: a) SUCRA ranking stability across the time-restricted primary analysis, meta-regression, prior sensitivity analyses, and alternative effect measure; b) CINeMA certainty of low or above for the comparison against UC-EX (the threshold was set at low rather than moderate as the 11-node network yielded low certainty for majority of the comparisons and a moderate threshold would have excluded nearly all interventions from the framework); and c) cross-outcome consistency, defined as ranking in the top four for both BCRL incidence and shoulder abduction ROM. The final interpretation integrated effect estimates, credible intervals, SUCRA stability, network sparsity, contribution matrix patterns, and CINeMA ratings.

All analyses were performed in R (version 4.3) using the gemtc (version 0.8-4) (12), netmeta, and coda packages.

## 3 Results

### 3.1 Literature screening and study characteristics

The database searches yielded 4,419 records, with three additional records identified through reference list screening, totaling 4,422 records. After deduplication, 2,749 records underwent title and abstract screening, of which 2,382 were excluded. The remaining 367 articles proceeded to full-text assessment. After excluding 267 articles (including one with unavailable full text), 100 RCTs were included in the qualitative synthesis. The PRISMA flow diagram is presented in **Figure 1**.

**Figure 1:**
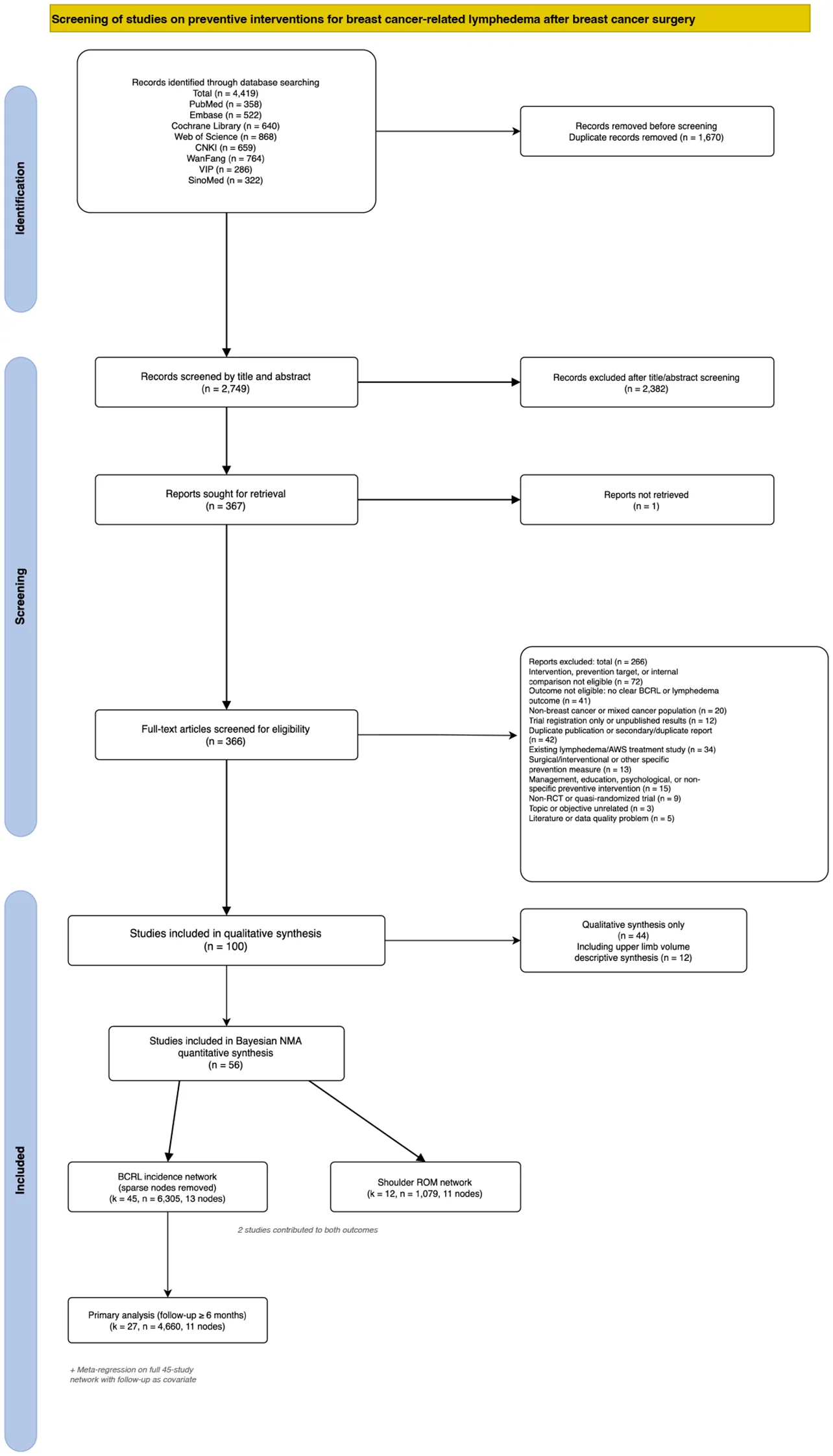
Preferred Reporting Items for Systematic Reviews and Meta-analyses (PRISMA) 2020 flow diagram for study selection.

Of the 100 included RCTs, 56 contributed data to at least one Bayesian NMA quantitative model (**Table 1**). The primary BCRL incidence analysis included 27 studies with follow-up of 6 months or longer. The full sparse-removed network of 45 studies was used in the meta-regression sensitivity analysis. The cleaned ROM analysis included 12 studies. An additional 12 studies reported upper limb volume data and were included in the descriptive synthesis. The remaining studies were retained in the qualitative synthesis and study characteristics summary.

**Table 1:** Characteristics of the 56 studies included in the Bayesian network meta-analysis (NMA) quantitative synthesis.

Author (year)	Country	Type	Participant characteristics			Intervention		Control	Outcome indicator
			*N*	Age (years)	Health status	Intervention	BCRL month		
Torres Lacomba et al. (2010) (18)	Spain	RCT	120	52.9 ± 10.7/52.9 ± 12.5	Postoperative BC; no baseline BCRL	MLD	12	UC-EX	BCRL incidence/severity, arm circumference, arm volume
Schmitz et al. (2010) (19)	USA	RCT	154	54 ± 8/56 ± 8	BC survivors at risk of BCRL	RT	12	UC-EX	BCRL incidence/severity
Sagen et al. (2009) (20)	Norway	RCT	204	54.9 ± 0.6/55.9 ± 0.5	Postoperative breast cancer	RT+SUP-EX	3	SUP-EX	BCRL incidence/severity, pain
Ochalek et al. (2019) (21)	Poland	RCT	45	NR	Postoperative breast cancer	COMP	12	UC-EX	BCRL incidence/severity, arm volume
Lin et al. (2023) (22)	China	RCT	200	51.69 ± 10.14/49.58 ± 10.91/47.37 ± 9.99/49.38 ± 9.51	Postoperative breast cancer	SUP-EX; RT+SUP-EX	6	UC-EX	QoL
Devoogdt et al. (2011) (23)	Belgium	RCT	160	55.8 ± 12.5/54.5 ± 11.1	Postoperative breast cancer	MLD	12	UC-EX	BCRL incidence/severity, arm circumference, arm volume
Castro-Sánchez et al. (2011) (24)	Spain	RCT	48	42.23 ± 9.42/49.28 ± 10.13	Postoperative BC receiving radiotherapy	COMP+MLD	8	UC-EX	Arm volume, QoL, pain
Bundred et al. (2023) (25)	UK	RCT	143	55.5(33.5-89.9)/55.8(32.0-86.9)	Postoperative BC; subclinical arm swelling	COMP	12	UC-EX	BCRL incidence/severity, QoL
Ammitzbøll et al. (2019) (26)	Denmark	RCT	158	53 ± 10/52 ± 10	Postoperative BC; no baseline BCRL	RT	12	UC-EX	BCRL incidence/severity, arm volume
Fan et al. (2026) (27)	China	RCT	114	51.54 ± 4.57/49.11 ± 9.35/46.73 ± 10.98	Postoperative BC; no baseline BCRL	RT	12	UC-EX	BCRL incidence/severity, arm circumference, strength
Xu et al. (2022) (28)	China	RCT	170	48.35 ± 7.12/47.14 ± 6.46	Postoperative breast cancer	AM+IPC	3	IPC	BCRL incidence/severity, shoulder ROM, QoL
Xiao et al. (2022) (29)	China	RCT	64	52.7 ± 8.3/50.6 ± 6.7	Postoperative BC receiving radiotherapy	ISO+KT	1.17	UC-EX	BCRL incidence/severity, shoulder ROM, upper limb function
Wang et al. (2022) (30)	China	RCT	160	54.1 ± 6.5/53.7 ± 6.2	Postoperative breast cancer	MLD	6	UC-EX	BCRL incidence/severity, shoulder ROM, QoL
Ou et al. (2023) (31)	China	RCT	80	42.7 ± 3.3/48.2 ± 2.5	Postoperative BC; no baseline BCRL	MLD	3	UC-EX	BCRL incidence/severity, shoulder ROM, pain
Yang et al. (2021) (32)	China	RCT	100	43.85 ± 8.67/43.91 ± 7.82	Postoperative breast cancer	RT	NA	UC-EX	BCRL incidence/severity, arm volume, shoulder ROM
Zhou (2024) (33)	China	RCT	70	48.84 ± 2.19/48.86 ± 2.15	Postoperative breast cancer	RT	3	UC-EX	BCRL incidence/severity
Huang (2021) (34)	China	RCT	72	44.86 ± 5.74/44.93 ± 5.78	Postoperative breast cancer	AM+IPC	3	AM	BCRL incidence/severity, upper limb function
Huang (2021) (35)	China	RCT	86	65.2 ± 2.4/64.9 ± 2.2	Postoperative breast cancer	SUP-EX	3	UC-EX	BCRL incidence/severity, shoulder ROM, strength
Huang (2022) (36)	China	RCT	120	40.20 ± 5.13/40.55 ± 5.21	Postoperative breast cancer	AM+IPC	1	AM	BCRL incidence/severity, shoulder ROM, QoL
Wang (2018) (37)	China	RCT	90	51.2 ± 4.3/51.3 ± 4.5	Postoperative breast cancer	MLD	6	UC-EX	BCRL incidence/severity
Lian et al. (2016) (38)	China	RCT	165	41.9 ± 8.2/42.6 ± 8.5/42.0 ± 9.1	Postoperative breast cancer	IPC+MLD; MLD	6	UC-EX	BCRL incidence/severity, arm circumference, upper limb function
Gao et al. (2014) (39)	China	RCT	60	NR	Postoperative breast cancer	IPC	3	UC-EX	BCRL incidence/severity
Ma et al. (2021) (40)	China	RCT	80	53.67 ± 4.85/54.12 ± 4.63	Postoperative breast cancer	MLD	12	UC-EX	BCRL incidence/severity
Ma et al. (2013) (41)	China	RCT	120	51.76 ± 4.80/50.23 ± 4.75	Postoperative breast cancer	IPC+MLD	NA	UC-EX	BCRL incidence/severity, shoulder ROM, pain, strength
Hao et al. (2015) (42)	China	RCT	72	NR	Postoperative breast cancer	AM	3	UC-EX	BCRL incidence/severity, upper limb function
Zhao et al. (2024) (43)	China	RCT	104	44.61 ± 8.46/45.30 ± 8.14	Postoperative breast cancer	RT	6	UC-EX	BCRL incidence/severity, QoL
Jia (2023) (44)	China	RCT	82	49.85 ± 6.38/50.12 ± 6.50	Postoperative breast cancer	AM+ISO	1	UC-EX	BCRL incidence/severity, shoulder ROM, upper limb function, QoL, strength
Luo et al. (2021) (45)	China	RCT	110	44.69 ± 5.36/44.36 ± 5.00	Postoperative breast cancer	IPC	6	UC-EX	BCRL incidence/severity, QoL, psychological outcomes
Cheng et al. (2021) (46)	China	RCT	86	47.82 ± 5.79/46.35 ± 6.27	Postoperative breast cancer	IPC	NA	UC-EX	BCRL incidence/severity, shoulder ROM
Chu et al. (2014) (47)	China	RCT	80	49.5/48.7	Postoperative breast cancer	IPC	6	UC-EX	Shoulder ROM, pain, complications
Shi et al. (2022) (48)	China	RCT	96	52.26 ± 5.79/51.98 ± 6.82	Postoperative breast cancer	KT	6	UC-EX	BCRL incidence/severity, arm circumference, shoulder ROM, upper limb function, QoL
Wang et al. (2021) (49)	China	RCT	88	51.46 ± 4.28/52.27 ± 4.49	Postoperative breast cancer	IPC_MLD	1	MLD	BCRL incidence/severity, shoulder ROM
Wang et al. (2020) (50)	China	RCT	90	NR	Postoperative breast cancer	MW; AM+MW	3	UC-EX	BCRL incidence/severity
Wang et al. (2019) (51)	China	RCT	200	NR	Postoperative breast cancer	MLD	6	UC-EX	BCRL incidence/severity
Wang et al. (2012) (52)	China	RCT	60	NR	Postoperative breast cancer	Mox	2	UC-EX	BCRL incidence/severity, shoulder ROM, upper limb function, strength
Wang (2018) (53)	China	RCT	280	51.4 ± 3.7/52.2 ± 3.5	Postoperative breast cancer	IPC+MLD	6	UC-EX	BCRL incidence/severity, arm circumference
Xiong et al. (2017) (54)	China	RCT	120	41.8 ± 1.6/42.7 ± 1.7	Postoperative breast cancer	IPC+MLD	12	UC-EX	BCRL incidence/severity
Yin et al. (2020) (55)	China	RCT	126	NR	Postoperative BC; no baseline BCRL	MLD; IPC+MLD	9	UC-EX	Shoulder ROM, upper limb function
Ouyang (2017) (56)	China	RCT	92	45.06 ± 4.23/44.85 ± 4.09	Postoperative BC receiving radiotherapy	IPC+MLD	6	UC-EX	BCRL incidence/severity, shoulder ROM
Liang et al. (2020) (57)	China	RCT	213	NR	Postoperative BC; no baseline BCRL	IPC+MLD	6	UC-EX	BCRL incidence/severity
Lin et al. (2021) (58)	China	RCT	80	44.50 ± 3.20/44.83 ± 3.11	Postoperative breast cancer	IPC	6	UC-EX	BCRL incidence/severity, shoulder ROM, upper limb function
Yang et al. (2019) (59)	China	RCT	86	46.54 ± 10.58/46.34 ± 11.58	Postoperative breast cancer	MW	12	UC-EX	BCRL incidence/severity, upper limb function, pain
Wu (2020) (60)	China	RCT	68	43.25 ± 5.86/43.37 ± 5.74	Postoperative BC; no baseline BCRL	COMP+RT	3	UC-EX	BCRL incidence/severity, arm volume, shoulder ROM
Li et al. (2018) (61)	China	RCT	116	47.53 ± 8.34/44.90 ± 10.00	Postoperative BC; no baseline BCRL	MLD	3	UC-EX	BCRL incidence/severity, QoL, pain
Li (2021) (62)	China	RCT	85	52.8 ± 3.2/52.5 ± 3.8	Postoperative breast cancer	IPC	3	UC-EX	BCRL incidence/severity, shoulder ROM, QoL
Li et al. (2019) (63)	China	RCT	1472	45.31 ± 10.05/44.46 ± 8.06	Postoperative BC; no baseline BCRL	COMP+RT	12	UC-EX	BCRL incidence/severity, upper limb function
Zhu et al. (2021) (64)	China	RCT	60	49.03 ± 4.78/48.41 ± 4.73	Postoperative breast cancer	MLD	3	UC-EX	BCRL incidence/severity, pain
Xu et al. (2009) (65)	China	RCT	100	NR	Postoperative BC receiving radiotherapy	IPC	NA	UC-EX	BCRL incidence/severity, shoulder ROM
Xu et al. (2020) (66)	China	RCT	120	45.5 ± 4.3/44.1 ± 4.8	Postoperative BC receiving radiotherapy	MLD	6	UC-EX	BCRL incidence/severity, arm volume
Zhang et al. (2014) (67)	China	RCT	70	NR	Postoperative breast cancer	MW	6	UC-EX	BCRL incidence/severity, strength
Liao (2015) (68)	China	RCT	80	NR	Postoperative breast cancer	COMP	3	UC-EX	BCRL incidence/severity
Jiang et al. (2014) (69)	China	RCT	90	NR	Postoperative breast cancer	COMP	3	UC-EX	BCRL incidence/severity
Yao et al. (2023) (70)	China	RCT	114	NR	Postoperative BC; no baseline BCRL	MLD; COMP+MLD	3	UC-EX	BCRL incidence/severity
Xia et al. (2018) (71)	China	RCT	80	58.4 ± 5.9/58.2 ± 6.3	Postoperative breast cancer	IPC	3	UC-EX	BCRL incidence/severity, shoulder ROM
Liu et al. (2021) (72)	China	RCT	88	58.96 ± 3.24/59.13 ± 3.58	Postoperative breast cancer	IPC+MLD	3	MLD	BCRL incidence/severity
He et al. (2025) (73)	China	RCT	130	52.36 ± 6.49/51.93 ± 6.27	Postoperative BC; no baseline BCRL	MLD+KT	1	UC-EX	BCRL incidence/severity, shoulder ROM, upper limb function, QoL

Intervention and breast cancer-related lymphedema (BCRL) month are taken directly from the supplied BCRL incidence workbook. For 11 ROM-only studies, intervention is taken from ROM_cleaned_13studies.csv and BCRL month is NA. Female was entered as N only when the source explicitly described the participants as women; otherwise, NR was retained.

Intervention types: MLD, manual lymphatic drainage; RT, resistance training; AE, aerobic exercise; IPC, intermittent pneumatic compression; COMP, compression garment/sleeve; AM, acupoint massage; MW, microwave therapy; ISO, isokinetic exercise; KT, kinesiotaping. Exercise/control terms: UC-EX, usual care or usual/unsupervised exercise; UNSUP-EX, unsupervised exercise; SUP-EX, supervised exercise; OTHER, other non-surgical intervention. Outcomes: BCRL, breast cancer-related lymphedema; ROM, range of motion; QoL, quality of life; NR, not reported; NA, not applicable.

The follow-up time distribution across the 45 studies in the sparse-removed BCRL network was heterogeneous: 18 studies reported data at 3 months, 14 studies at 6 months, 2 studies at 8–9 months, and 11 studies at 12 months. Among the 40 studies with available surgical data, 38 (95%) exclusively enrolled patients who had undergone ALND. Radiotherapy rates were reported in only 16 of 45 studies (36%). By publication language, 82 studies were published in Chinese and 18 in English.

### 3.2 Risk of bias

Risk of bias assessment using the Cochrane RoB 2 tool classified 14 studies as low risk and 86 as some concerns; no study was judged to be at high overall risk of bias. The primary sources of concern were insufficient reporting of randomization sequence generation and allocation concealment and the inherent difficulty of blinding participants and personnel in rehabilitation and physical therapy interventions. The RoB 2 summary is presented in **Figure 2**.

**Figure 2:**
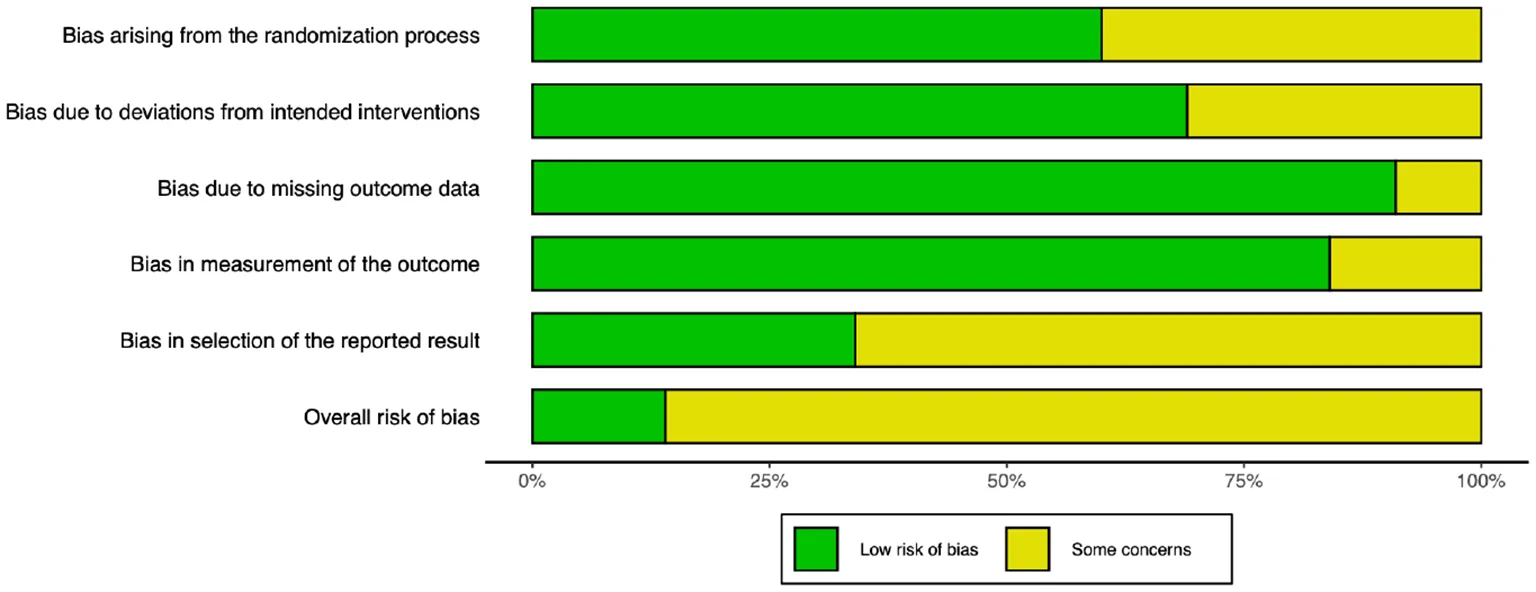
Risk of bias summary: proportion of studies at each level of risk for each domain (Cochrane RoB 2).

### 3.3 Primary outcome: BCRL incidence (≥6 months follow-up)

#### 3.3.1 Network description

The primary analysis included 27 studies with follow-up of at least 6 months, comprising 57 study arms, 4,660 participants, and 11 intervention nodes. No double-zero-event or single-zero-event studies were present. The network structure is presented in **Figure 3A**. All 27 studies contributed a UC-EX reference arm. The control arm BCRL event rates varied across studies (median = 0.250, IQR = 0.187–0.312; range = 0.099–0.617, pooled 510/2,292 = 0.223). Because 4 of 10 active nodes were supported by a single study and multi-arm trials contributed the same control arm rate to more than one node, the between-node differences were summarized descriptively rather than tested inferentially, and no clear systematic pattern was apparent.

**Figure 3:**
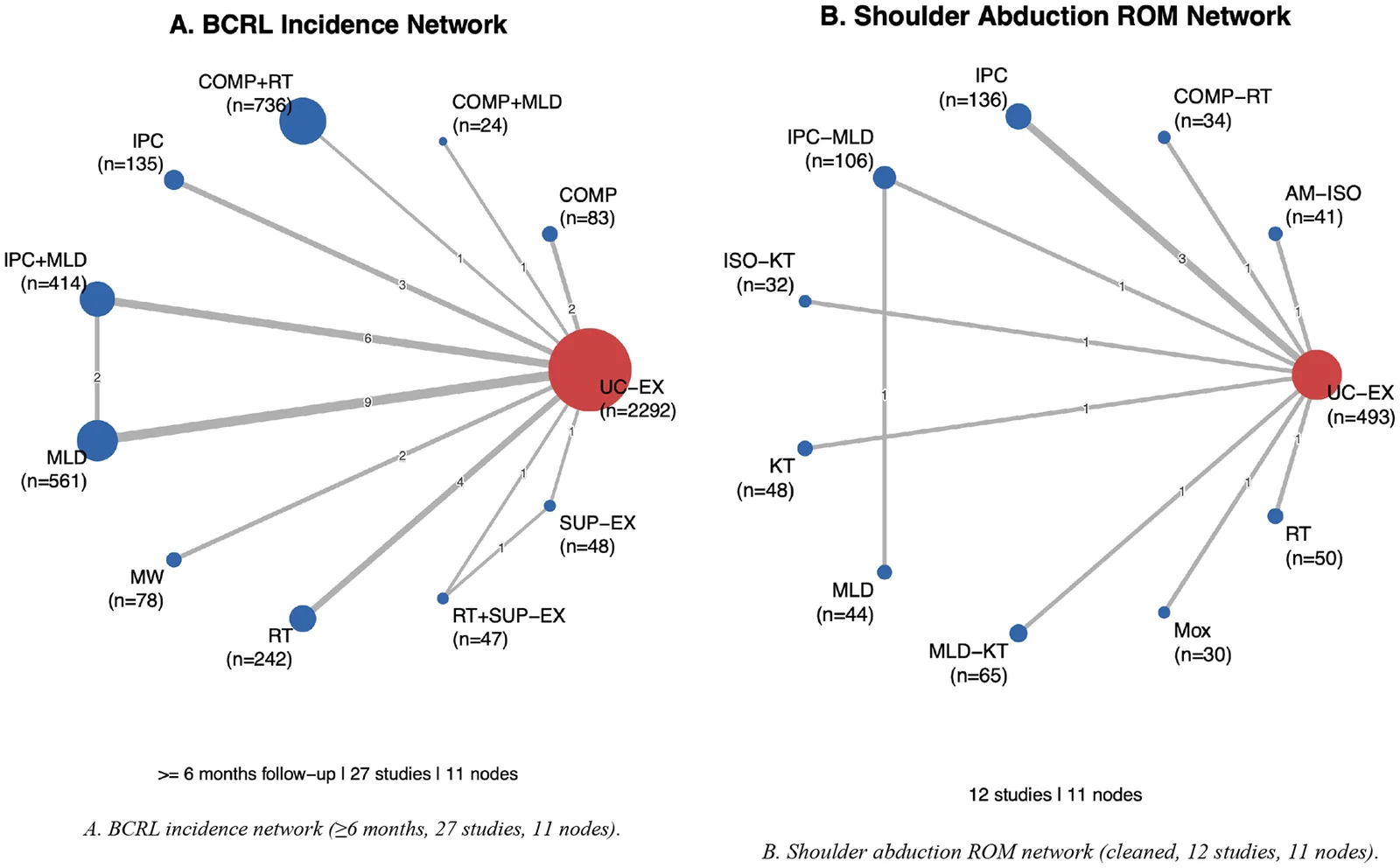
**(A)** Breast cancer-related lymphedema (BCRL) incidence network (≥6 months, 27 studies, 11 nodes). **(B)** Shoulder abduction range of motion (ROM) network (cleaned, 12 studies, 11 nodes).

#### 3.3.2 Model convergence and fit

The Bayesian random-effects consistency model converged satisfactorily. The maximum Gelman–Rubin Rhat upper confidence limit was 1.003 and the minimum ESS was 8,387, both well within acceptable thresholds. The model DIC was 100.93. The between-study heterogeneity parameter had a posterior median of *τ* = 0.352 [95% credible interval (95%CrI) = 0.097–0.650], corresponding to *τ*^2^ = 0.124, indicating moderate heterogeneity across studies.

#### 3.3.3 Treatment effects and rankings

Relative to UC-EX, 7 of 10 active interventions showed statistically meaningful reductions in BCRL risk, with 95%CrIs excluding 1. Ordered by SUCRA ranking, these were: COMP+RT (RR = 0.146, 95%CrI = 0.057–0.366, SUCRA = 85.1%), RT+SUP-EX (RR = 0.147, 95%CrI = 0.019–0.665, SUCRA = 80.8%), COMP+MLD (RR = 0.121, 95%CrI = 0.004–0.915, SUCRA = 80.6%), IPC+MLD (RR = 0.255, 95%CrI = 0.153–0.397, SUCRA = 69.3%), microwave therapy (MW) (RR = 0.313, 95%CrI = 0.090–0.926, SUCRA = 57.8%), IPC (RR = 0.396, 95%CrI = 0.197–0.765, SUCRA = 47.8%), and MLD (RR = 0.467, 95%CrI = 0.320–0.650, SUCRA = 39.2%).

COMP+RT ranked first by SUCRA with the narrowest credible interval among the top three, reflecting the relative precision of its effect estimate. COMP+MLD had the lowest point estimate (RR = 0.121), but ranked third by SUCRA as its wide credible interval—spanning from 0.004 to 0.915 based on a single study with 48 participants (24 per arm)—diluted its cumulative ranking probability. This dissociation between point estimate and SUCRA rank illustrates the influence of estimation precision on Bayesian ranking probabilities.

The remaining three active interventions had credible intervals crossing 1: SUP-EX (RR = 0.485, 95%CrI = 0.138–1.539, SUCRA = 38.1%), COMP (RR = 0.612, 95%CrI = 0.260–1.377, SUCRA = 26.6%), and RT (RR = 0.672, 95%CrI = 0.368–1.167, SUCRA = 21.3%). UC-EX ranked last (SUCRA = 3.5%). The complete treatment effect estimates and SUCRA values are presented in **Table 2** and **Figures 4** and **5**.

**Table 2:** Breast cancer-related lymphedema (BCRL) incidence: Bayesian network meta-analysis (NMA) primary analysis results (≥6 months *vs*. UC-EX).

Intervention	RR (median)	95% CrI lower	95% CrI upper	SUCRA (%)
COMP+RT	**0.146**	0.057	0.366	85.1
RT+SUP-EX	**0.147**	0.019	0.665	80.8
COMP+MLD	**0.121**	0.004	0.915	80.6
IPC+MLD	**0.255**	0.153	0.397	69.3
MW	**0.313**	0.090	0.926	57.8
IPC	**0.396**	0.197	0.765	47.8
MLD	**0.467**	0.320	0.650	39.2
SUP-EX	0.485	0.138	1.539	38.1
COMP	0.612	0.260	1.377	26.6
RT	0.672	0.368	1.167	21.3
UC-EX	–	–	–	3.5

Values in bold indicate that the 95% CrI excludes 1.

SUCRA, surface under the cumulative ranking curve; COMP, compression therapy; RT, resistance training; SUP-EX, supervised exercise; MLD, manual lymphatic drainage; IPC, intermittent pneumatic compression; MW, microwave therapy; UC-EX, usual care/education with unsupervised exercise.

**Figure 4:**
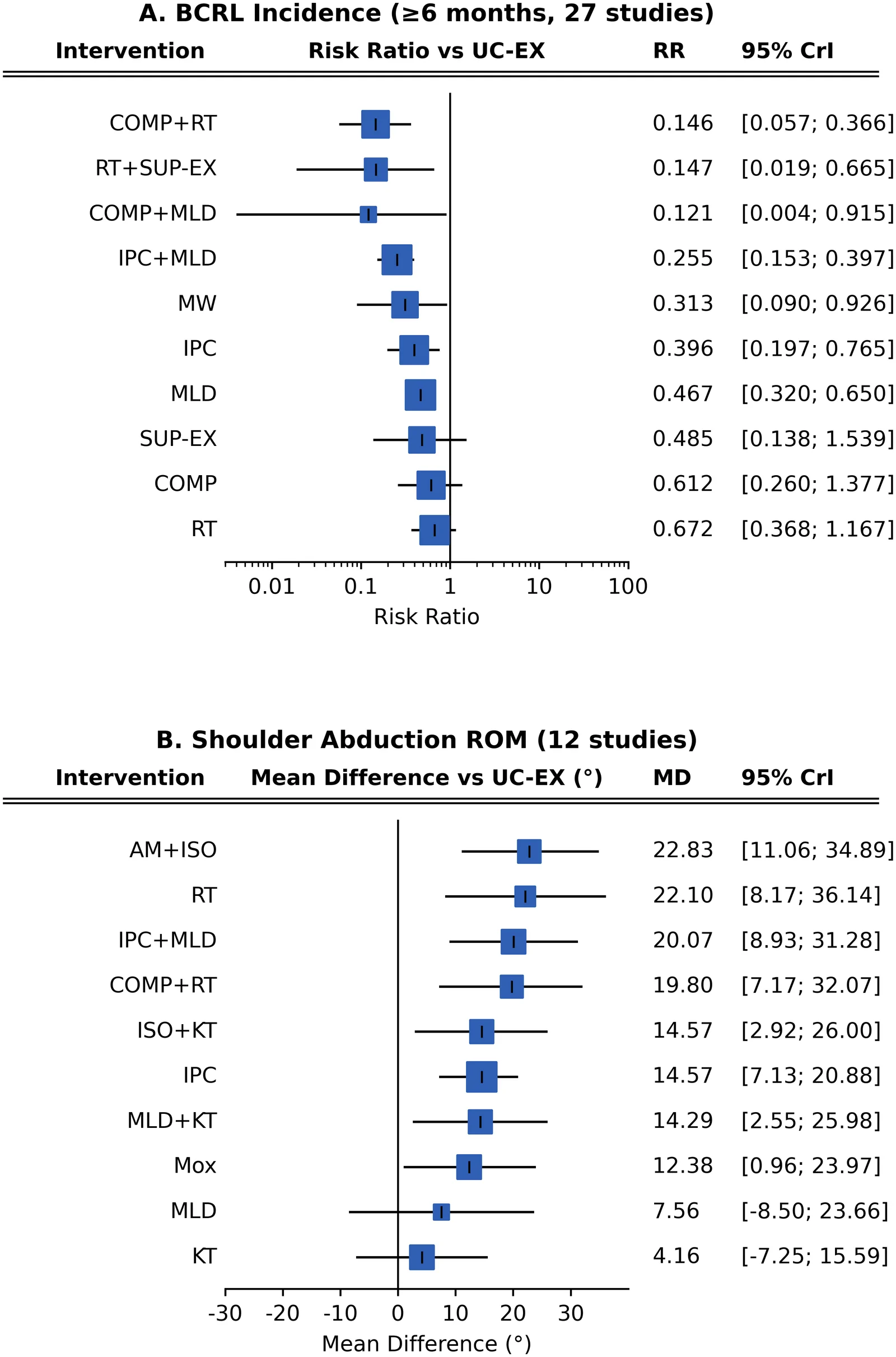
Forest plots. **(A)** Risk ratio (RR) *vs*. UC-EX (usual care/education with unsupervised exercise), breast cancer-related lymphedema (BCRL) incidence. **(B)** Mean difference (MD) *vs*. UC-EX, shoulder abduction range of motion (ROM).

**Figure 5:**
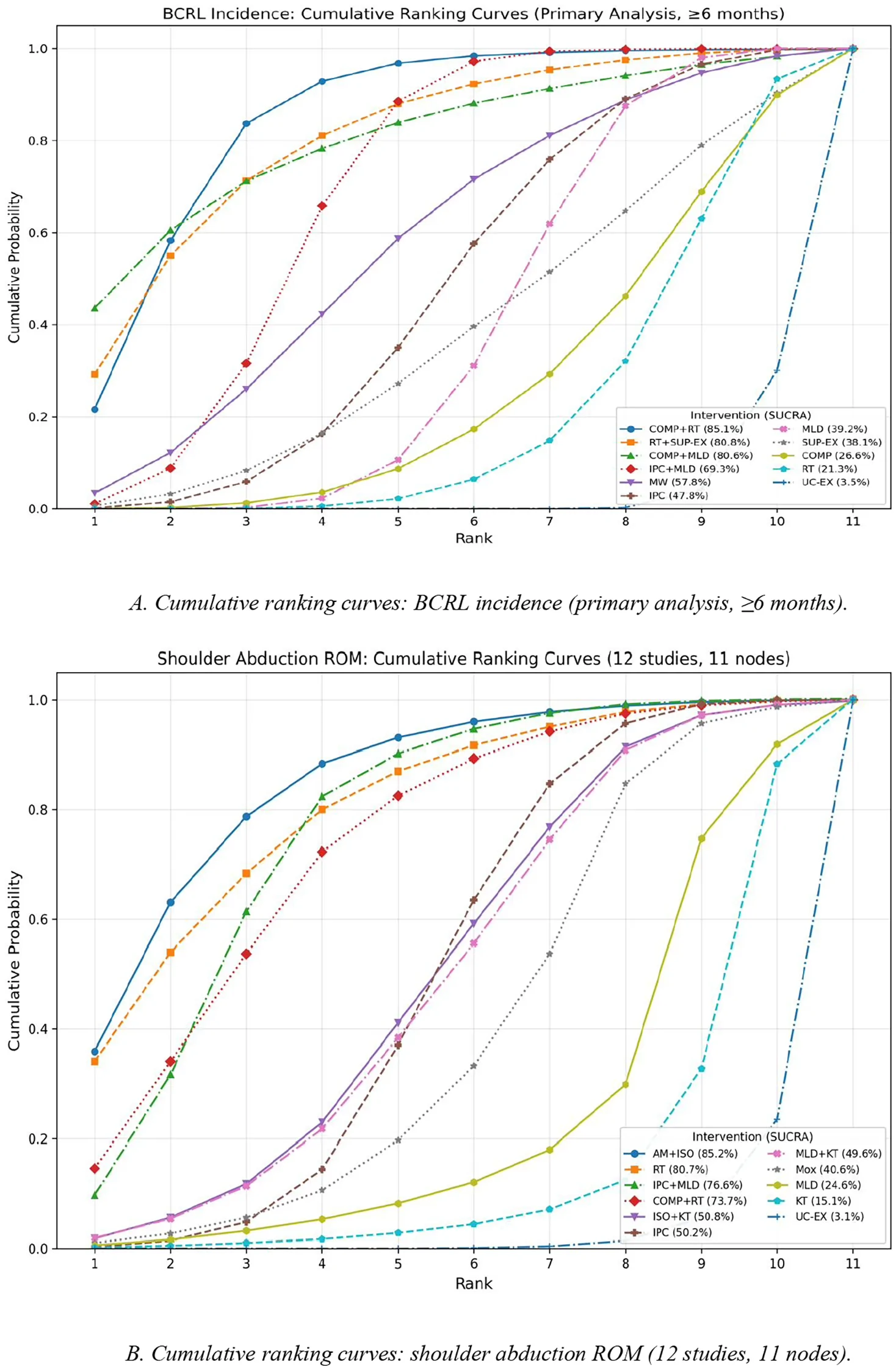
Cumulative ranking curves. **(A)** Breast cancer-related lymphedema (BCRL) incidence (primary analysis, ≥6 months). **(B)** Shoulder abduction range of motion (ROM) (12 studies, 11 nodes).

#### 3.3.4 Consistency assessment

The UME model yielded a DIC of 100.93, which was identical to that of the consistency model, providing no evidence of global inconsistency. The network structure permitted node splitting for one comparison (IPC+MLD *vs*. MLD), which showed no significant discrepancy between direct and indirect evidence (*p* = 0.264). The remaining comparisons lacked closed loops and were therefore structurally non-splittable.

#### 3.3.5 Publication bias

The comparison-adjusted funnel plot (**Figure 6**) showed visual asymmetry, and the Egger regression test was statistically significant (*p* = 0.008), indicating potential small-study effects. This finding is consistent with the high proportion of Chinese-language publications (82%) and the predominance of small single-center trials in the network.

**Figure 6:**
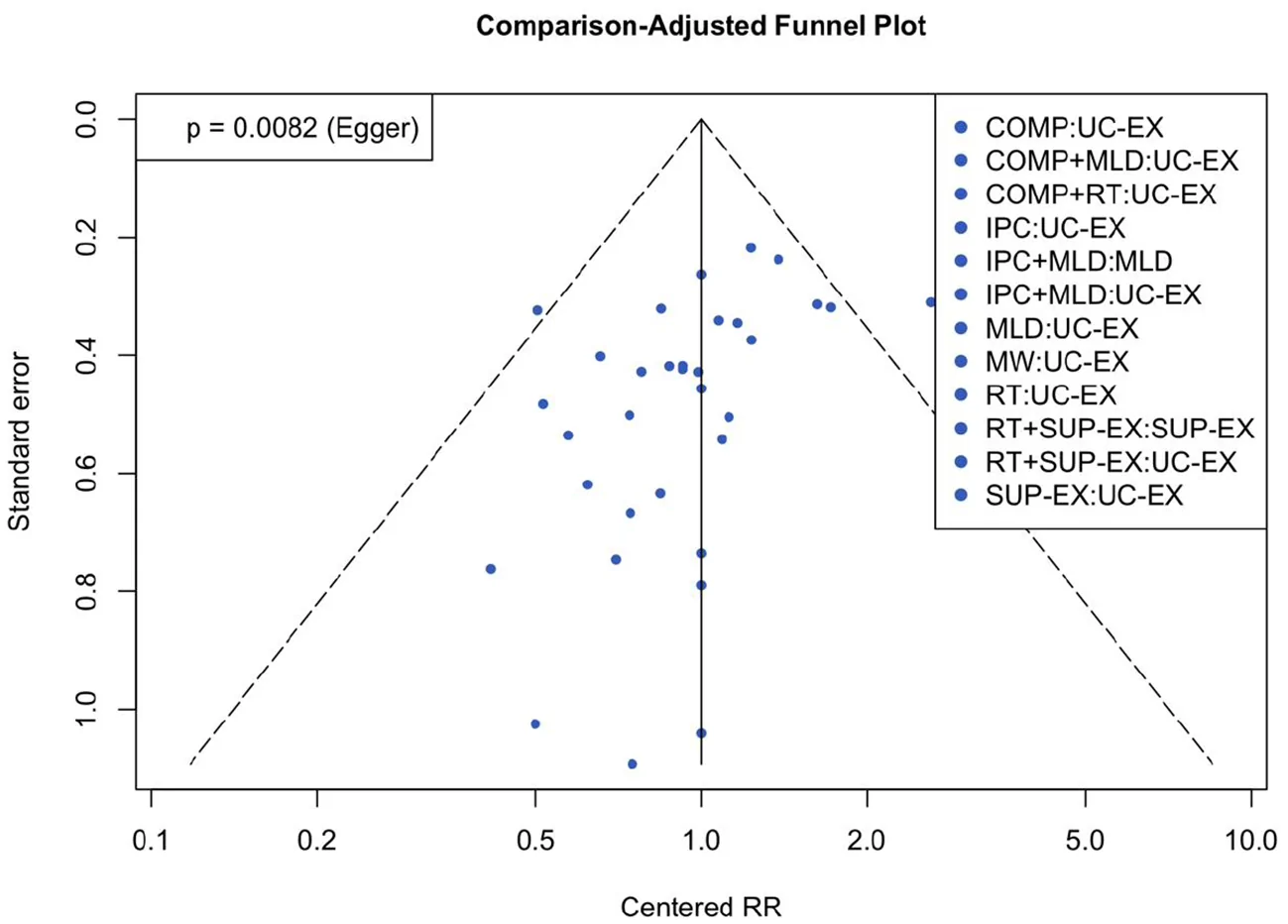
Comparison-adjusted funnel plot: breast cancer-related lymphedema (BCRL) incidence (Egger *p* = 0.008).

### 3.4 Sensitivity analyses for the primary outcome

#### 3.4.1 Sparse-removed network without time restriction (45 studies)

The full sparse-removed network included 45 studies, 94 arms, 6,305 participants, and 13 nodes. Compared with the primary analysis, this network incorporated 18 additional studies with follow-up of less than 6 months and retained two additional intervention nodes: AM and AM+IPC. AM+IPC ranked first (SUCRA = 89.2%, RR = 0.117, 95%CrI = 0.040–0.319) and COMP+RT ranked second (SUCRA = 85.5%, RR = 0.143, 95%CrI = 0.065–0.295). All evidence supporting AM and AM+IPC came exclusively from studies with less than 6 months of follow-up. The Egger test was more strongly significant in this broader network (*p* = 0.0003), and the between-study heterogeneity was somewhat lower (*τ* = 0.293, 95%CrI = 0.044–0.519) than that in the primary analysis (*τ* = 0.352).

#### 3.4.2 Diagnostic criteria and publication language restrictions

To assess the influence of diagnostic heterogeneity on the rankings, two prespecified subsets were analyzed after applying within-subset sparse node removal. The volumetric-only subset (class 1A) retained seven studies, five nodes, and 15 arms, while the clear definition subset (classes 1A and 1B) retained 29 studies, 10 nodes, and 61 arms (one double-zero-event study excluded). In both subsets, COMP+RT appeared in only one study and was removed as a sparse node, indicating that its evidence in the primary analysis relies predominantly on studies using author-defined diagnostic thresholds (class 1D).

In the volumetric-only analysis, RT+SUP-EX ranked first (SUCRA = 90.8%, RR = 0.245, 95%CrI = 0.054–0.911), followed by SUP-EX (68.0%) and COMP (49.3%). Heterogeneity was moderate (*τ* = 0.317, 95%CrI = 0.016–1.204). In the 1A + 1B analysis, AM+IPC ranked first (SUCRA = 95.7%, RR = 0.126, 95%CrI = 0.043–0.331), followed by RT+SUP-EX (SUCRA = 77.0%, RR = 0.236, 95%CrI = 0.076–0.654) and IPC+MLD (SUCRA = 74.8%, RR = 0.267, 95%CrI = 0.173–0.390). There were six of nine active treatments that achieved credible intervals excluding the null. Heterogeneity was lower (*τ* = 0.215, 95%CrI = 0.014–0.504), consistent with the reduced diagnostic variability in this subset.

An exploratory analysis restricted to non-Chinese-language publications (six studies, four nodes after sparse node removal) yielded a small star-shaped network in which no intervention achieved a credible interval excluding the null (COMP: RR = 0.604, 95%CrI = 0.150–2.207; MLD: RR = 0.679, 95%CrI = 0.165–2.246; and RT: RR = 0.898, 95%CrI = 0.244–3.125), reflecting limited statistical power. The full dataset sensitivity results are provided in **Supplementary Table 11**.

#### 3.4.3 Network meta-regression adjusting for follow-up time

A shared-coefficient meta-regression on all 45 studies with follow-up duration (months) as a covariate yielded a regression coefficient of *β* = 0.077 per month (95%CrI = 0.026–0.128). The credible interval excluded zero, indicating that follow-up duration significantly modified the relative treatment effects: each additional month of follow-up was associated with an 8.0% attenuation in relative risk reduction (RR multiplier of 1.080 per month). The DIC of the meta-regression model (150.22) was lower than that of the unadjusted full-network model (155.75), and the residual heterogeneity decreased by 38% (*τ* = 0.181 *vs*. 0.293), confirming that follow-up duration accounted for a substantial portion of the between-study variability.

After adjustment, COMP+RT remained first (SUCRA = 93.5%), followed by AM+IPC (84.4%), COMP+MLD (83.9%), and IPC+MLD (70.4%). The SUCRA gap between COMP+RT and AM+IPC widened from 3.7 percentage points in the unadjusted network to 9.1 points after time adjustment, indicating that the apparent competitiveness of AM+IPC in the full network was partly attributable to its short follow-up window.

#### 3.4.4 Alternative effect measure

A sensitivity analysis using ORs (logit link) on the same 27 primary analysis studies confirmed the stability of the findings. COMP+RT remained first (SUCRA = 79.4%), followed by RT+SUP-EX (78.9%), COMP+MLD (78.7%), and IPC+MLD (74.5%). The same seven interventions showed credible intervals excluding the null, and the Spearman’s rank correlation between the RR and OR SUCRA rankings was 1.000.

#### 3.4.5 Prior sensitivity

Treatment effect priors were varied to Normal(0, 5^2^) and Normal(0, 1.5^2^), while the heterogeneity priors were varied to HalfNormal(0, 0.5^2^), HalfNormal(0, 2^2^), and Uniform(0, 2). COMP+RT ranked first across all six prior specifications (including the baseline), with a maximum rank change of two positions among the second- through fourth-ranked interventions. The full prior sensitivity results are provided in **Supplementary Table 5**.

#### 3.4.6 Cross-analysis ranking stability

COMP+RT ranked first in three of six analytical approaches: the time-restricted primary analysis, the meta-regression, and the OR sensitivity analysis (**Table 3**). In the sparse-removed full network, it ranked second (SUCRA = 85.5% *vs*. 89.2% for AM+IPC). However, in both the volumetric-only (1A) and clear definition (1A + 1B) subsets, COMP+RT appeared in only one study and was excluded as a sparse node; RT+SUP-EX and AM+IPC ranked first in these subsets, respectively. This indicates that the top ranking of COMP+RT is robust across analytical methods and time restrictions, but is contingent on the inclusion of studies using author-defined (class 1D) diagnostic thresholds.

**Table 3:** Surface under the cumulative ranking curve (SUCRA) comparisons across analytical approaches.

Intervention	Primary (RR) (%)	Primary (OR) (%)	Meta-regression (%)	Sparse-removed (45 studies) (%)	1A volumetric (7 studies) (%)	1A+1B (29 studies) (%)
COMP+RT	85.1	79.4	93.5	85.5	–	–
RT+SUP-EX	80.8	78.9	54.0	57.6	90.8	77.0
COMP+MLD	80.6	78.7	83.9	85.4	–	–
IPC+MLD	69.3	74.5	70.4	65.9	–	74.8
MW	57.8	55.6	51.1	45.6	–	–
IPC	47.8	49.4	41.0	49.6	–	58.6
MLD	39.2	43.7	29.9	28.7	–	37.3
SUP-EX	38.1	37.4	42.6	48.0	68.0	56.0
COMP	26.6	27.2	39.6	33.7	49.3	28.5
RT	21.3	21.4	27.1	17.7	30.1	13.6
UC-EX	3.5	3.8	0.6	0.5	11.8	4.3
AM	–	–	31.9	42.6	–	54.2
AM+IPC	–	–	84.4	89.2	–	95.7

RR, risk ratio; OR, odds ratio; COMP, compression therapy; RT, resistance training; SUP-EX, supervised exercise; MLD, manual lymphatic drainage; IPC, intermittent pneumatic compression; MW, microwave therapy; UC-EX, usual care/education with unsupervised exercise.

### 3.5 Secondary outcome: shoulder abduction ROM

#### 3.5.1 Data cleaning

The original ROM dataset comprised 18 studies. Five were excluded based on prespecified cleaning criteria. Studies 059 and 106 reported change from baseline scores rather than post-treatment absolute values, making them incompatible with the remaining studies. Study 102 measured ROM at 30 days postoperatively, when shoulder abduction had decreased from baseline (from 92° to 75°–87°), reflecting acute surgical restriction rather than functional recovery. Study 097 showed no intergroup difference at 12 weeks (87° *vs*. 87°). Study 026, although reporting absolute values, measured ROM at 3 months when patients were still severely restricted (67° and 53°), clinically incomparable with the 130°–170° range observed in the other studies. After these exclusions, study 041 (AM+IPC *vs*. AM) became disconnected from the reference node UC-EX. This study and its two nodes (AM and AM+IPC) were therefore excluded from the network, yielding 12 studies with 11 nodes.

#### 3.5.2 Network description and temporal coverage

The cleaned network comprised 12 studies, 24 study arms, 1,079 participants, and 11 intervention nodes. Of 12 studies, 10 reported outcomes within the first 1–3 months postoperatively, representing the critical window for early shoulder rehabilitation during which abduction recovers most rapidly and the functional gap between interventions and usual care is most clinically apparent. One study provided data at 6 months. One study did not specify the exact follow-up duration, but reported values consistent with early-to-mid recovery. The network structure is presented in **Figure 3B**.

#### 3.5.3 Model results

The Bayesian random-effects consistency model converged satisfactorily (maximum Rhat upper CI = 1.003, minimum ESS = 6,095). The model DIC was 46.41. The between-study heterogeneity was moderate (*τ* = 2.828, 95%CrI = 0.130–13.241, *τ*^2^ ≈ 7.998). The UME model showed no improvement in fit (ΔDIC = 0.04), supporting the consistency assumption. No comparisons were structurally splittable as the network was entirely star-shaped through UC-EX. The Egger regression test was statistically significant (*p* = 0.037), indicating potential small-study effects. The comparison-adjusted funnel plot is presented in **Figure 7**.

**Figure 7:**
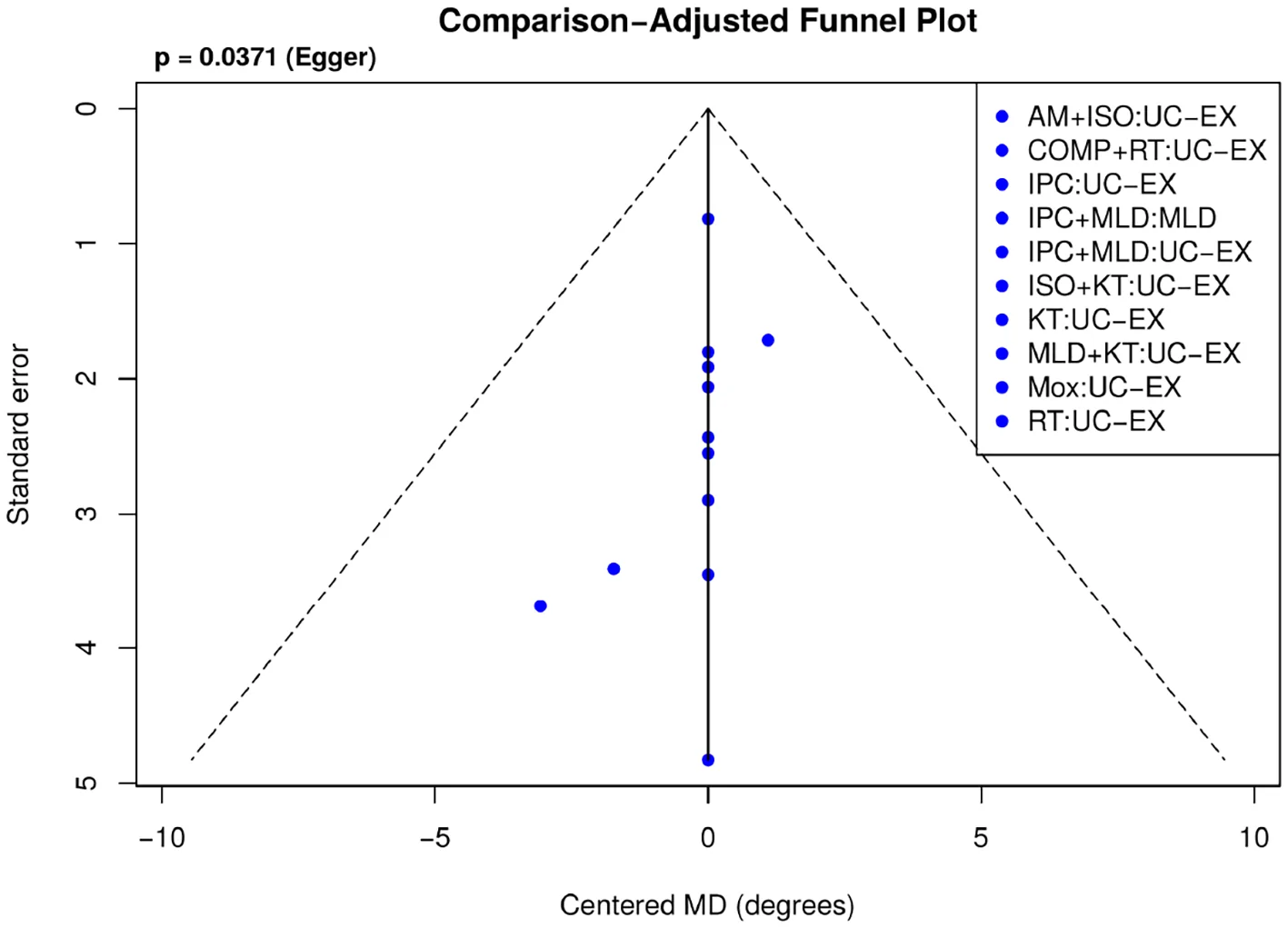
Comparison-adjusted funnel plot: shoulder abduction range of motion (ROM) (Egger *p* = 0.037).

Out of 10 active interventions, eight demonstrated statistically meaningful improvements in shoulder abduction relative to UC-EX, with 95%CrIs excluding zero, ordered by SUCRA ranking: AM+ISO (MD = 22.83°, 95%CrI = 11.06–34.89, SUCRA = 85.2%), RT (MD = 22.10°, 95%CrI = 8.17–36.14, SUCRA = 80.7%), IPC+MLD (MD = 20.07°, 95%CrI = 8.93–31.28, SUCRA = 76.6%), COMP+RT (MD = 19.80°, 95%CrI = 7.17–32.07, SUCRA = 73.7%), ISO+KT (MD = 14.57°, 95%CrI = 2.92–26.00, SUCRA = 50.8%), IPC (MD = 14.57°, 95%CrI = 7.13–20.88, SUCRA = 50.2%), MLD+KT (MD = 14.29°, 95%CrI = 2.55–25.98, SUCRA = 49.6%), and Mox (MD = 12.38°, 95%CrI = 0.96–23.97, SUCRA = 40.6%). MLD (MD = 7.56°, 95%CrI = −8.50 to 23.66) and KT (MD = 4.16°, 95%CrI = −7.25 to 15.59) showed favorable point estimates, but with credible intervals crossing zero. The complete results are presented in **Table 4** and **Figures 4B** and **5B**.

**Table 4:** Shoulder abduction range of motion (ROM): Bayesian network meta-analysis (NMA) results (*vs*. UC-EX).

Intervention	MD (deg)	95%CrI lower	95%CrI upper	SUCRA (%)
AM+ISO	**22.83**	11.06	34.89	85.2
RT	**22.10**	8.17	36.14	80.7
IPC+MLD	**20.07**	8.93	31.28	76.6
COMP+RT	**19.80**	7.17	32.07	73.7
ISO+KT	**14.57**	2.92	26.00	50.8
IPC	**14.57**	7.13	20.88	50.2
MLD+KT	**14.29**	2.55	25.98	49.6
Mox	**12.38**	0.96	23.97	40.6
MLD	7.56	−8.50	23.66	24.6
KT	4.16	−7.25	15.59	15.1
UC-EX	–	–	–	3.1

Values in bold indicate that the 95% CrI excludes 0.

SUCRA, surface under the cumulative ranking curve; MD, mean difference; AM, acupoint massage; ISO, isokinetic strength training; RT, resistance training; IPC, intermittent pneumatic compression; MLD, manual lymphatic drainage; COMP, compression therapy; UC-EX, usual care/education with unsupervised exercise; Mox, moxibustion; KT, kinesiotaping.

#### 3.5.4 Prior sensitivity

Prior sensitivity analyses confirmed the stability of the ROM rankings. AM+ISO, RT, and IPC+MLD consistently occupied the top three positions, and COMP+RT remained fourth, across all prior specifications tested (**Supplementary Table 5**).

### 3.6 Secondary outcome: upper limb volume difference (descriptive synthesis)

A total of 12 studies reported upper limb volume data. As described in *Section 2.6.3*, NMA was not performed for this outcome due to the irreconcilable heterogeneity in the construct measured (inter-limb difference *vs*. change from baseline) and the implausible standard deviations in five studies. Among the 12 studies, combination interventions (COMP+MLD+RT, COMP+MLD, and COMP+RT) showed point estimates favoring reduced volume difference compared with UC-EX, and single-modality interventions (MLD, RT, and Mox) showed directional trends toward benefit. The magnitude of these differences varied substantially, and the data quality concerns preclude quantitative ranking. This outcome is interpreted as a directional supplement to the BCRL incidence and ROM analyses, without independent weight in the integrated interpretation.

### 3.7 Certainty of evidence

#### 3.7.1 BCRL incidence

CINeMA assessment evaluated all pairwise comparisons across six domains (**Table 5**). Within-study bias was rated as some concerns for all comparisons, reflecting the predominance of some concerns judgments in the RoB 2 assessment (86 of 100 studies). Reporting bias was rated as some concerns based on the significant Egger test (*p* = 0.008). Indirectness was rated as no concerns. Heterogeneity was rated as some concerns (*τ* = 0.352). Incoherence was rated as no concerns (ΔDIC = 0, node-splitting *p* > 0.05).

**Table 5:** Confidence in Network Meta-Analysis (CINeMA) certainty of evidence summary.

Outcome	Within-study bias	Reporting bias	Indirectness	Imprecision	Heterogeneity	Incoherence	Predominant certainty
BCRL incidence (≥6 months)	Some concerns	Some concerns	No concerns	Varies	Some concerns	No concerns	Low
Shoulder abduction ROM	Some concerns	Some concerns	No concerns	Varies	Some concerns	No concerns	Low/very low

BCRL, breast cancer-related lymphedema; ROM, range of motion.

For the top-ranked comparisons, COMP+RT *vs*. UC-EX was rated low certainty (downgraded for within-study bias and reporting bias, with a narrow credible interval partially offsetting imprecision concerns), IPC+MLD *vs*. UC-EX was rated moderate (supported by six study arms and 414 participants), and MLD *vs*. UC-EX was rated moderate (supported by nine study arms and 561 participants). The majority of the remaining comparisons were rated low or very low, primarily due to imprecision and sparse evidence.

#### 3.7.2 Shoulder abduction ROM

Within-study bias was rated as some concerns. Reporting bias was rated as some concerns (Egger *p* = 0.037). Indirectness was rated as no concerns. Heterogeneity was rated as some concerns (*τ* ≈ 2.8°, moderate relative to effect magnitudes of 12°–23°). Incoherence was rated as no concerns (ΔDIC = 0.04). The eight comparisons with credible intervals excluding zero were rated low certainty. The remaining comparisons were rated very low.

#### 3.7.3 Cross-outcome integration

Two interventions simultaneously ranked in the top four for both outcomes—COMP+RT (BCRL: SUCRA = 85.1%, rank 1; ROM: SUCRA = 73.7%, rank 4) and IPC+MLD (BCRL: SUCRA = 69.3%, rank 4; ROM: SUCRA = 76.6%, rank 3)—both with credible intervals excluding the null in both analyses. Under the prespecified integrated interpretation framework (*Section 2.12*), both satisfied cross-outcome consistency and CINeMA certainty of low or above. However, their evidence profiles differed: COMP+RT had the highest BCRL SUCRA with stable rankings across three of six analytical approaches, but was supported predominantly by a single large trial and rated low certainty for BCRL. IPC+MLD had a lower BCRL SUCRA, but was supported by multiple direct comparisons and rated moderate certainty for BCRL. COMP+RT thus showed the strongest ranking signal, whereas IPC+MLD showed a more balanced profile across ranking, replication, and certainty. The remaining interventions met one or no criteria.

## 4 Discussion

### 4.1 Combination interventions outperform single-modality approaches

The most consistent finding across all analyses was the dominance of the combination interventions. COMP+RT, RT+SUP-EX, COMP+MLD, and IPC+MLD—all two-component combinations—occupied the top four SUCRA positions, whereas the highest-ranked single-modality interventions (MW and IPC) ranked fifth and sixth, respectively. This pattern was robust across the time-restricted primary analysis, the meta-regression, and the OR sensitivity analysis.

The superiority of combination strategies is consistent with the multifactorial pathophysiology of BCRL. Postoperative lymphedema develops through a cascade of lymphatic drainage impairment, interstitial fluid accumulation, reduced muscle pump function, chronic inflammation, and progressive fibrosis. Single-modality interventions target one segment of this cascade: compression limits fluid accumulation, MLD promotes superficial lymphatic transport, and RT enhances the skeletal muscle pump. Combination strategies engage multiple pathophysiological targets simultaneously. COMP+RT, for instance, pairs external compression with active muscle pump forces—a dual mechanism that may account for its consistent advantage across both BCRL incidence and ROM outcomes.

This finding resonates with the established logic of complete decongestive therapy (CDT), the criterion-standard multimodal treatment for established lymphedema, which combines compression bandaging, MLD, decongestive exercises, and skincare (74). The effectiveness of CDT is attributed precisely to its engagement of multiple fluid transport pathways simultaneously rather than reliance on any single mechanism. That the same principle—multimodal targeting of the lymphatic–venous drainage axis—appears to govern preventive efficacy suggests a continuity between the pathophysiological rationale for treatment and prevention. From a mechanistic standpoint, COMP exerts sustained external hydrostatic pressure that limits interstitial fluid accumulation and maintains tissue pressure gradients favoring lymphatic absorption, while RT generates rhythmic skeletal muscle contractions that produce intermittent compressive forces on intramuscular and perimuscular lymphatic and venous vessels, facilitating antegrade flow through the one-way valve system of collecting lymphatics (75). When applied concurrently, the static pressure gradient from compression and the dynamic pumping from muscle contraction may act synergistically: compression prevents retrograde fluid pooling during the relaxation phase of each contraction, while muscle pump activation augments the pressure gradient that drives lymphatic propulsion. This dual-pathway model is consistent with lymphatic physiology research demonstrating that approximately one-third of lower extremity lymph transport at rest is attributable to the extrinsic skeletal muscle pump and two-thirds to intrinsic lymphangion contractility and that external compression can augment both components (75).

This interpretation carries an important caveat. The ranking advantage of combination interventions may partly reflect a dose–response effect—patients receiving two active interventions receive more therapeutic contact—rather than true mechanistic synergy between components. Factorial trial designs that separately test each component and their combination would be required to isolate the independent contribution of each element.

### 4.2 COMP+RT: evidence basis for the primary recommendation

COMP+RT ranked first for BCRL incidence in three of six analytical approaches: the time-restricted primary analysis (SUCRA = 85.1%), the meta-regression (93.5%), and the OR sensitivity analysis (79.4%). In the full sparse-removed network, it ranked second (SUCRA = 85.5%), within 3.7 percentage points of the top-ranked AM+IPC. Its RR of 0.146 (95%CrI = 0.057–0.366) corresponds to an 85% reduction in BCRL risk relative to usual care. Prior sensitivity analyses confirmed that this ranking was invariant to the choice of treatment effect and heterogeneity priors. However, when the analysis was restricted to studies using volumetric or circumferential diagnostic criteria (classes 1A and 1A + 1B), COMP+RT was represented by only one study and was excluded as a sparse node, indicating that its evidence base is concentrated among studies using author-defined diagnostic thresholds.

COMP+RT was one of two interventions that simultaneously ranked in the top four for both outcomes—BCRL incidence (rank 1) and shoulder abduction ROM (MD = 19.80°, 95%CrI = 7.17–32.07, rank 4)—alongside IPC+MLD (BCRL rank 4; ROM: MD = 20.07°, 95%CrI = 8.93–31.28, rank 3). The BCRL and ROM analyses captured complementary postoperative windows—the 6- to 36-month BCRL risk period and the 1- to 3-month rapid rehabilitation period—and the cross-outcome consistency of COMP+RT suggests that it may address both early functional recovery and longer-term lymphedema prevention. Under the prespecified integrated interpretation framework, both COMP+RT and IPC+MLD satisfied cross-outcome consistency and the CINeMA certainty criteria. COMP+RT additionally demonstrated the most stable SUCRA ranking across sensitivity analyses, although its evidence was concentrated in a single large trial with low CINeMA certainty. IPC+MLD, in contrast, was supported by multiple direct comparisons and with moderate CINeMA certainty for BCRL incidence.

From a clinical rehabilitation perspective, the cross-outcome profile of COMP+RT has a practical implication that extends beyond ranking statistics. The BCRL incidence analysis and the ROM analysis captured two distinct but sequential phases of the postoperative trajectory: shoulder abduction ROM recovers most rapidly during the first 1–3 months, when patients transition from acute surgical restriction toward functional independence, while the risk of BCRL peaks between 6 and 12 months in ALND populations (2), when cumulative lymphatic overload exceeds compensatory capacity. An intervention that ranks favorably in both windows suggests utility across the full rehabilitation arc—from early postoperative functional recovery through to the subsequent lymphedema surveillance period. This temporal complementarity has implications for clinical pathway design: rather than prescribing compression for long-term prevention and a separate exercise protocol for early mobility, COMP+RT may serve as a unified intervention that addresses both objectives within a single program. However, the current evidence does not establish whether COMP+RT should be initiated immediately postoperatively, after wound healing, or at a later stage. The optimal timing, intensity, and duration of each component remain to be defined by future dose-finding studies.

### 4.3 Follow-up duration as an effect modifier

The meta-regression yielded a statistically significant regression coefficient (*β* = 0.077/month, 95%CrI = 0.026–0.128), indicating that the relative treatment effects attenuated by approximately 8% per additional month of follow-up. This finding has two implications.

First, it validates the ≥6-month time restriction used in the primary analysis. Studies with short follow-up capture a period before the BCRL incidence peak in ALND populations, when the event rates are systematically lower and the relative treatment effects may appear inflated. The significant *β* value confirms that mixing short- and long-follow-up studies in a single network introduces heterogeneity that is not purely random but reflects a structured time–treatment interaction. This finding has methodological relevance for future NMA of BCRL prevention: follow-up duration should be treated as an effect modifier rather than ignored.

Second, it contextualizes the AM+IPC signal. AM+IPC ranked first in the full sparse-removed network (SUCRA = 89.2%), but was absent from the primary analysis because all supporting evidence came from studies with less than 6 months of follow-up. After time adjustment, AM+IPC retained second place (SUCRA = 84.4%), but the SUCRA gap with COMP+RT widened from 3.7 to 9.1 percentage points. Whether the early benefit of AM+IPC translates into sustained prevention during the 6- to 12-month peak risk window remains unknown and requires trials with extended follow-up.

### 4.4 Comparison with prior reviews

Previous systematic reviews of BCRL prevention have focused on individual intervention categories. Hayes et al. (3) evaluated exercise and found it safe and potentially effective, but could not compare it against non-exercise interventions. The Cochrane review by McNeely et al. (76) similarly synthesized exercise interventions for upper limb dysfunction after breast cancer treatment, an outcome domain that overlaps with our secondary ROM analysis. Similarly to Hayes et al., it addressed a single intervention category and could not rank exercise against compression- or drainage-based strategies. That review concluded that exercise can result in a significant and clinically meaningful improvement in shoulder ROM and found no evidence of an increased risk of lymphedema from exercise at any time point. Su et al. (5) examined IPC and reported a pooled RR of 0.36 (95%CI = 0.22–0.58), consistent with our estimate for IPC (RR = 0.396, 95%CrI = 0.197–0.765). Rafn et al. (4) demonstrated that prospective surveillance with triggered early intervention reduces chronic BCRL, but did not compare specific preventive modalities. The MASCC clinical practice guidance (6) provided consensus-based recommendations using the Delphi methodology, but lacked quantitative comparative effectiveness data. The Academy of Oncologic Physical Therapy (AOPT) clinical practice guideline (16) similarly addressed lymphedema interventions, but focused on treatment rather than comparative prevention.

This study extends the evidence base by simultaneously comparing all intervention categories within a single framework, by explicitly addressing the time-dependent nature of BCRL incidence through time-restricted analysis with meta-regression validation, and by applying the CINeMA framework to quantify the evidence certainty for each network comparison.

### 4.5 Limitations

Several limitations should be considered. First, the network combined studies using different BCRL diagnostic criteria (volumetric, circumferential, and author-defined thresholds). When restricted to studies with objective diagnostic definitions (classes 1A and 1A + 1B), COMP+RT—the top-ranked intervention in the primary analysis—appeared in only one study and was excluded as a sparse node; RT+SUP-EX and AM+IPC ranked first in those subsets, respectively. This suggests that the comparative ranking of COMP+RT under stricter diagnostic definitions remains uncertain and warrants further investigation with standardized outcome measurement.

Second, radiotherapy exposure—a known modifier of late-onset BCRL risk—could not be assessed as a transitivity variable because 64% of the studies did not report radiotherapy rates.

Third, the NMA for upper limb volume was abandoned due to irreconcilable data quality issues. Although directional findings were consistent with the BCRL incidence results, the absence of a quantitative volume analysis limits triangulation across outcomes.

Fourth, the ROM analysis primarily covered the first 1–3 months postoperatively. Whether early functional gains are maintained longer term cannot be determined from these data. The complementarity between the BCRL time window (≥6 months) and the ROM time window (1–3 months) captures two distinct phases of recovery, but neither outcome alone covers the full trajectory.

Fifth, COMP+MLD ranked third in the primary analysis with a credible interval excluding 1 (0.004–0.915), but was supported by a single study with 48 participants (24 per arm). The interval spans nearly three orders of magnitude, and this ranking requires confirmation in additional trials.

Sixth, the network was dominated by star-shaped connections through UC-EX, with limited direct comparisons between active interventions. The majority of relative effects rely substantially on indirect evidence.

Seventh, the Egger regression test was significant in both the BCRL (*p* = 0.008) and ROM (*p* = 0.037) networks, and the high proportion of Chinese-language publications (82%) with predominantly some concerns RoB ratings raises concerns about small-study effects. The Egger *p*-value attenuated from 0.0003 in the full network to 0.008 in the time-restricted primary analysis, suggesting that the exclusion of short-term studies—predominantly small Chinese-language trials—partially reduced this effect.

Eighth, the control arm event rates varied approximately sixfold across the primary analysis studies (0.099–0.617). Although no clear systematic pattern across intervention nodes was apparent, COMP+RT—the top-ranked intervention—was supported by a single study whose control arm event rate (0.145) was the fourth lowest of the 27 studies and well below the overall median (0.250). Because the RR is not invariant to the baseline risk, part of the apparent magnitude of the COMP+RT effect may reflect the comparatively low baseline risk in its single supporting trial. This observation is descriptive and does not establish a causal explanation for the ranking, but reinforces the need for confirmatory trials in populations with documented baseline risk profiles.

### 4.6 Clinical implications

While these findings should not be interpreted as definitive clinical recommendations given the predominantly low certainty of evidence, they carry several implications for clinical practice, guideline development, and postoperative care delivery.

Compression alone is insufficient: active exercise is essential. A notable finding with immediate clinical relevance is the contrast between COMP alone and COMP+RT. COMP as a single-modality preventive strategy did not achieve a credible interval excluding the null (RR = 0.612, 95%CrI = 0.260–1.377, SUCRA = 26.6%), whereas COMP+RT ranked first (RR = 0.146, 95%CrI = 0.057–0.366, SUCRA = 85.1%). This disparity is consistent with the PLACE randomized trial (25), in which prophylactic compression sleeves alone after ALND did not significantly prevent lymphedema at 24 months in the overall cohort. The convergence of our network-level finding with the head-to-head result from the PLACE trial reinforces the interpretation that passive compression without concurrent activation of the skeletal muscle pump provides only a partial barrier against interstitial fluid accumulation. From a clinical standpoint, this implies that prescribing a compression garment without an accompanying structured RT program may not confer meaningful preventive benefit and that clinicians should view the two components as complementary rather than substitutable.

Integration with the prospective surveillance model (PSM): the PSM, as originally described by Stout et al. (77) and subsequently implemented at multiple cancer centers, provides a framework for early detection and triggered intervention through periodic postoperative limb monitoring. Our meta-regression finding that the follow-up duration significantly modifies the relative treatment effects (*β* = 0.077/month, 95%CrI = 0.026–0.128) provides quantitative support for the core premise of PSM: the timing of intervention matters as treatment effects are not constant across the postoperative trajectory. Specifically, the attenuation of relative risk reduction with longer follow-up suggests that preventive interventions exert their greatest benefit during the early-to-mid postoperative period, before chronic tissue remodeling diminishes responsiveness. This aligns with the PSM philosophy of proactive rather than reactive management and suggests that COMP+RT or other combination strategies should be initiated within the first months after surgery rather than deferred until subclinical edema is detected. Future implementation research should evaluate how the preventive interventions identified in this NMA can be embedded within the existing PSM pathways to create an integrated surveillance-and-prevention protocol.

Resource-stratified recommendations: Clinical implementation will necessarily depend on locally available resources, expertise, and patient preferences. Based on the hierarchy of evidence in this NMA, a three-tier framework may be considered. For institutions with access to rehabilitation specialists and structured exercise programs, COMP+RT represents the first-line combination, given its top ranking in three of six analytical approaches and its dual benefit for both lymphedema prevention and shoulder function. For settings with physical therapy services but without formal RT infrastructure, IPC+MLD offers an alternative with moderate CINeMA certainty evidence (SUCRA = 69.3%, RR = 0.255, 95%CrI = 0.153–0.397) and a narrower credible interval that reflects its larger contributing evidence base (six study arms, 414 participants). For primary care or community settings where access to specialized lymphedema services is limited, single-modality interventions with credible intervals excluding the null—particularly MLD (SUCRA = 39.2%, moderate CINeMA certainty) and IPC (SUCRA = 47.8%)—may represent feasible starting points, with referral to multimodal programs when available. This stratification is not intended as a rigid protocol, but as a decision-support heuristic that acknowledges the reality of heterogeneous healthcare delivery systems.

Safety considerations: None of the active interventions evaluated in this analysis was associated with increased BCRL risk or impaired shoulder function relative to usual care. This safety profile is particularly relevant for RT, which has historically been discouraged in breast cancer survivors due to theoretical concerns that increased upper limb blood flow and muscle loading might provoke or exacerbate lymphedema. Multiple randomized trials and systematic reviews have since demonstrated that progressive RT does not increase the lymphedema incidence or severity in women with or who are at risk of BCRL (3, 19, 76), and at least one long-term follow-up study has confirmed sustained preventive benefits at 3.5 years (78). The present NMA extends these safety findings to the network-level comparison, providing additional reassurance that combination interventions incorporating RT can be recommended without evidence of harm.

These implications should be weighed against individual patient preferences, comorbidities, treatment-related factors (including radiotherapy exposure and extent of axillary surgery), and the specific risk profile of each patient. The predominantly low certainty of evidence underscores that these suggestions represent the best available evidence rather than strong guideline-grade recommendations and that shared decision-making between clinicians and patients remains essential.

### 4.7 Implications for future research

COMP+RT should be evaluated in large multicenter RCTs with standardized BCRL diagnostic criteria and follow-up of at least 12 months to confirm its cross-outcome benefit profile. AM+IPC requires trials with extended follow-up (≥12 months) to determine whether its short-term signal translates into durable prevention. Head-to-head trials comparing the top-ranked combinations (e.g., COMP+RT *vs*. IPC+MLD) would provide the direct evidence currently absent from the network. Future primary studies should adopt standardized volumetric diagnostic criteria, report outcomes at both 6 and 12 months, and systematically document ALND extent and radiotherapy exposure. Future syntheses should also consider multivariate NMA to model BCRL incidence and shoulder function jointly. This was not feasible here: the primary ≥6-month BCRL network (27 studies) shared no studies with the analyzed ROM network (12 studies), and even in the all-follow-up 45-study BCRL network, only two studies overlapped with the ROM network, with both reporting BCRL at 3 months. The within-study correlation between outcomes therefore could not be estimated from the data and would have had to be assumed, and a joint model would contain many unobserved treatment–outcome combinations. Primary trials reporting both outcomes at common time points would make a joint model informative.

## 5 Conclusion

Among the 11 non-surgical interventions evaluated in this Bayesian NMA, COMP+RT (compression therapy combined with resistance training) demonstrated the most robust evidence profile. It ranked first for BCRL incidence reduction in three of six analytical approaches and was one of two interventions—alongside IPC+MLD—that simultaneously ranked in the top four for both BCRL incidence and shoulder abduction ROM. However, sensitivity analyses restricted to studies with volumetric or circumferential diagnostic criteria revealed that COMP+RT appeared in only one such study, and its top ranking was not maintained under these stricter definitions. Seven interventions showed credible intervals excluding the null for BCRL incidence, and eight showed credible intervals excluding zero for ROM. The meta-regression confirmed that follow-up duration significantly modifies the treatment effects (*β* = 0.077/month), validating the ≥6-month time restriction and highlighting the need for adequate follow-up in future prevention trials. IPC+MLD also ranked in the top four for both outcomes and was supported by moderate certainty BCRL evidence, representing a well-replicated alternative. RT+SUP-EX is an additional option with credible intervals excluding the null for BCRL, although without corresponding ROM network evidence. AM+IPC demonstrated a strong short-term signal that persisted after time adjustment, but lacks evidence beyond 3 months. These findings should be interpreted in the context of predominantly low certainty evidence, and confirmatory trials with standardized diagnostic criteria and follow-up of at least 12 months are needed.

## Data Availability

The original contributions presented in the study are included in the article/**Supplementary Material**. Further inquiries can be directed to the corresponding author.
