# Supplementary material for: Comparative effectiveness of non-surgical interventions and their combinations for the prevention of breast cancer-related lymphedema: a systematic review and Bayesian network meta-analysis
Source: Front Oncol. 2026 Sep 21;16:1940372. doi: 10.3389/fonc.2026.1940372 (PMC13634858; doi:10.3389/fonc.2026.1940372)
Supplement: Supplementary file 1 [file Table1.docx]

**Supplementary Appendix 1. Supplementary Materials**

**Supplementary Material S1. Complete search strategies**

The original search strategies for each database are presented below. Original Chinese search terms are retained for Chinese-language databases to preserve reproducibility.

The original search strategies for each database are presented below. To avoid line breaks within long search strings in tables, this supplementary material presents the strategies by database. Original Chinese search terms are retained for Chinese-language databases to preserve reproducibility.

**CNKI**

TKA = ('乳腺癌' + '乳腺肿瘤' + '乳腺恶性肿瘤') AND TKA = ('淋巴水肿' + '上肢淋巴水肿' + '术后淋巴水肿' + '上肢水肿' + '臂水肿' + '乳腺癌相关淋巴水肿') AND TKA = ('针灸' + '针刺' + '电针' + '艾灸' + '温针灸' + '温针' +'穴位按摩' + '穴位按压' + '点按' + '按摩' + '推拿' +'气压治疗' + '空气波' + '间歇充气加压' + '充气加压' + '气压波' +'淋巴引流' + '徒手淋巴引流' + '手法淋巴引流' + '淋巴引流按摩' +'加压' + '压力治疗' + '弹力袖套' + '压力袖套' + '弹力绷带' + '压力绷带' + '压迫疗法' + '压力衣' + '物理治疗' + '理疗' + '康复' + '康复治疗' + '康复训练' + '功能训练' + '功能锻炼' + '上肢功能锻炼' + '运动疗法' + '运动训练' + '锻炼' + '阻力训练' + '抗阻训练' + '力量训练' + '伸展' + '拉伸' + '关节活动' + '关节活动度' + '活动度训练' + '肩关节活动' + 'ROM' + '瑜伽' + '太极' + '太极拳' + '八段锦' + '气功' + '导引' + '穴位贴敷' + '中药湿敷' + '中药熏洗' + '中药塌渍' + '刮痧' + '拔罐' + '中药热奄包') AND FT =('随机' + '随机分组' + '随机对照')

**Wanfang**

主题:(乳腺癌 or 乳腺肿瘤 or 乳腺恶性肿瘤) and 主题:(淋巴水肿 or 上肢淋巴水肿 or 术后淋巴水肿 or 上肢水肿 or 臂水肿) and 主题:(针灸 or 针刺 or 电针 or 艾灸 or 温针灸 or 温针 or 穴位按摩 or 穴位按压 or 按摩 or 推拿 or 点按 or 气压治疗 or 空气波 or 间歇充气加压 or 充气加压 or 淋巴引流 or 徒手淋巴引流 or 手法淋巴引流 or 加压 or 弹力袖套 or 压力治疗 or 物理治疗 or 理疗 or 康复 or 康复治疗 or 康复训练 or 功能训练 or 功能锻炼 or 上肢功能锻炼 or 运动疗法 or 运动训练 or 锻炼 or 阻力训练 or 抗阻训练 or 力量训练 or 伸展 or 拉伸 or 关节活动 or 活动 or 关节活动度 or 活动度训练 or 肩关节活动 or ROM or 瑜伽 or 太极 or 太极拳 or 八段锦 or 气功 or 导引 or 穴位贴敷 or 中药湿敷 or 中药熏洗 or 中药塌渍 or 刮痧 or 拔罐 or 中药热奄包) 全部:(随机 or 随机分组 or 随机对照)

**VIP Database**

(M=(乳腺癌 OR 乳腺肿瘤 OR 乳腺恶性肿瘤) OR K=(乳腺癌 OR 乳腺肿瘤 OR 乳腺恶性肿瘤)) AND (M=(淋巴水肿 OR 上肢淋巴水肿 OR 术后淋巴水肿 OR 上肢水肿 OR 臂水肿) OR K=(淋巴水肿 OR 上肢淋巴水肿 OR 术后淋巴水肿 OR 上肢水肿 OR 臂水肿)) AND (M=(针灸 OR 针刺 OR 电针 OR 艾灸 OR 温针灸 OR 温针 OR 穴位按摩 OR 穴位按压 OR 按摩 OR 推拿 OR 点按 OR 气压治疗 OR 空气波 OR 间歇充气加压 OR 充气加压 OR 淋巴引流 OR 徒手淋巴引流 OR 手法淋巴引流 OR 加压 OR 弹力袖套 OR 压力治疗) OR K=(针灸 OR 针刺 OR 电针 OR 艾灸 OR 温针灸 OR 温针 OR 穴位按摩 OR 穴位按压 OR 按摩 OR 推拿 OR 点按 OR 气压治疗 OR 空气波 OR 间歇充气加压 OR 充气加压 OR 淋巴引流 OR 徒手淋巴引流 OR 手法淋巴引流 OR 加压 OR 弹力袖套 OR 压力治疗))

**SinoMed**

1	6）"乳腺癌"[常用字段:智能] OR "乳腺肿瘤"[常用字段:智能] OR "乳腺恶性肿瘤"[常用字段:智能] OR "乳腺肿瘤"[不加权:扩展]
2	7）"淋巴水肿"[常用字段:智能] OR "上肢淋巴水肿"[常用字段:智能] OR "术后淋巴水肿"[常用字段:智能] OR "上肢水肿"[常用字段:智能] OR "臂水肿"[常用字段:智能] OR "淋巴水肿"[不加权:扩展] OR "乳腺癌淋巴水肿"[不加权:扩展] OR "乳腺癌淋巴水肿"[不加权:扩展] OR "乳腺癌淋巴水肿"[不加权:扩展] OR "乳腺癌淋巴水肿"[不加权:扩展] OR "乳腺癌淋巴水肿"[不加权:扩展]
3	8）"加压"[常用字段:智能] OR "弹力袖套"[常用字段:智能] OR "压力治疗"[常用字段:智能] OR "推拿"[常用字段:智能] OR "点按"[常用字段:智能] OR "气压治疗"[常用字段:智能] OR "空气波"[常用字段:智能] OR "间歇充气加压"[常用字段:智能] OR "充气加压"[常用字段:智能] OR "淋巴引流"[常用字段:智能] OR "徒手淋巴引流"[常用字段:智能] OR "手法淋巴引流"[常用字段:智能] OR "针灸"[常用字段:智能] OR "针刺"[常用字段:智能] OR "电针"[常用字段:智能] OR "艾灸"[常用字段:智能] OR "温针灸"[常用字段:智能] OR "温针"[常用字段:智能] OR "穴位按摩"[常用字段:智能] OR "穴位按压"[常用字段:智能] OR "按摩"[常用字段:智能] OR "按摩"[不加权:扩展] OR "针灸疗法"[不加权:扩展] OR "温针疗法"[不加权:扩展] OR "针灸疗法"[不加权:扩展] OR "推拿疗法"[不加权:扩展] OR "推拿疗法"[不加权:扩展] OR "点穴疗法"[不加权:扩展] OR "穴位按压"[不加权:扩展] OR "淋巴引流按摩"[不加权:扩展] OR "长袜, 压力"[不加权:扩展]
4	9）"运动疗法"[不加权:扩展]
5	10）"耐力训练"[常用字段:智能] OR "运动疗法"[常用字段:智能] OR "持续被动性肌牵张运动"[常用字段:智能] OR "抗阻训练"[常用字段:智能] OR "牵张训练"[常用字段:智能]
6	14）"外治疗法"[不加权:扩展] OR "冲洗疗法"[不加权:扩展] OR "拔罐疗法"[不加权:扩展] OR "敷贴疗法"[不加权:扩展] OR "溻渍疗法"[不加权:扩展] OR "湿敷疗法"[不加权:扩展] OR "刮痧疗法"[不加权:扩展] OR "热敷疗法"[不加权:扩展]
7	15）(#14) OR (#10) OR (#9) OR (#8)
8	16）(#15) AND (#7) AND (#6)
9	17）((#15) AND (#7) AND (#6)) AND ( 随机对照试验[文献类型])

**PubMed**

("Breast Neoplasms"[MeSH Terms] OR ("Breast Neoplasms"[Title/Abstract] OR "breast tumors"[Title/Abstract] OR "breast cancer"[Title/Abstract])) AND ("lymphoedema"[Title/Abstract] OR "Lymphedema"[Title/Abstract] OR "lymph* edem*"[Title/Abstract] OR "lymph* oedem*"[Title/Abstract] OR ("lymph*"[All Fields] AND "swell"[Title/Abstract]) OR "elephantias*"[Title/Abstract] OR "BCRL"[Title/Abstract] OR "Lymphedema"[MeSH Terms]) AND ("randomized controlled trial"[Publication Type] OR "controlled clinical trial"[Publication Type] OR "randomized"[Title/Abstract] OR "randomized controlled trials as topic"[MeSH Major Topic] OR "randomly"[Title/Abstract]) AND ("Physical Therapy Modalities"[MeSH Terms] OR ("low intensity laser therapy"[Title/Abstract] OR "manual lymphatic drainage"[Title/Abstract] OR "MLD"[Title/Abstract] OR "massage interventions"[Title/Abstract] OR "chinese massage"[Title/Abstract] OR "compression sleeves"[Title/Abstract] OR "compression therapy"[Title/Abstract] OR "skin care"[Title/Abstract] OR "intermittent pneumatic compression pump"[Title/Abstract] OR "IPC"[Title/Abstract] OR "pneumatic cuffs"[Title/Abstract] OR "thermal therapy"[Title/Abstract] OR "Exercise"[Title/Abstract] OR "resistance training"[Title/Abstract] OR "progressive resistance training"[Title/Abstract] OR "ROM"[Title/Abstract] OR "rehabilitation exercise"[Title/Abstract] OR "aquatic exercises"[Title/Abstract] OR "aerobic"[Title/Abstract] OR "anaerobic"[Title/Abstract] OR "kinesiology taping"[Title/Abstract] OR "decongestive lymphatic therapy"[Title/Abstract] OR "DLT"[Title/Abstract]) OR ("Acupuncture"[MeSH Terms] OR "Moxibustion"[MeSH Terms] OR ("Acupuncture"[Title/Abstract] OR "acupunctur*"[Title/Abstract] OR "acupress*"[Title/Abstract] OR "needling"[Title/Abstract] OR "electric-acupuncture"[Title/Abstract] OR "Zhenjiu"[Title/Abstract] OR "meridian"[Title/Abstract] OR "ching lo"[Title/Abstract] OR "jing luo"[Title/Abstract] OR "Moxibustion"[Title/Abstract] OR "needl"[Title/Abstract] OR "acupuncture therapy"[Title/Abstract] OR ("Acupuncture"[Title/Abstract] AND "Moxibustion"[Title/Abstract]) OR "warm needle"[Title/Abstract] OR "blood letting puncture"[Title/Abstract] OR ("blood letting puncture"[Title/Abstract] AND "cupping"[Title/Abstract]) OR "electroacupuncture*"[Title/Abstract] OR "acupoint"[Title/Abstract] OR "acupoint massage"[Title/Abstract] OR "acupoint application"[Title/Abstract]) OR ("Fumigation"[Title/Abstract] OR "Fumigations"[Title/Abstract] OR "Washing"[Title/Abstract])))

**Web of Science Core Collection Search Strategy**

Database: Web of Science Core Collection. Run date: 24 Jan 2026.

ID: #1

Search strategy: English: (((TS=( Breast Neoplasms)) OR AB=(breast cancer)) OR AB=(breast tumor)) OR AB=(breast neoplasm)

Hits: 422062

ID: #2

Search strategy: English: (((TS=(lymphedema)) OR AB=('lymphedema)) OR AB=(breast cancer-related lymphedema)) OR AB=(postmastectomy lymphedema)

Hits: 13703

ID: #3

Search strategy: English: #2 AND #1

Hits: 3399

ID: #4

Search strategy: English: (((((((((((((((((((((TS=(acupuncture)) OR AB=(acupuncture)) OR AB=(acupressure)) OR AB=(acupuncture analgesia)) OR AB=(auricular acupuncture)) OR AB=(catgut embedding)) OR AB=(electroacupuncture)) OR AB=( pharmacopuncture)) OR AB=(blood-letting puncture)) OR AB=(moxibustion)) OR AB=(needling)) OR AB=(zhenjiu)) OR AB=( meridian)) OR AB=(ching lo)) OR AB=(meridian system)) OR AB=(warm acupuncture)) OR AB=(acupuncture point)) OR AB=(acupoint massage)) OR AB=(acupoint application)) OR AB=(fumigation)) OR AB=(washing)) OR TS=(moxibustion)

Hits: 319844

ID: #5

Search strategy: English: ((((((((((((((((((TS=(Physical Therapy Modalities)) OR AB=(low level laser therapy)) OR AB=(manual lymphatic drainage)) OR AB=(massage)) OR AB=(chinese massage)) OR AB=(compression sleeve)) OR AB=(compression therapy)) OR AB=(skin care)) OR AB=(intermittent pneumaticcompression pump)) OR AB=('pneumatic cuffs)) OR AB=(thermotherapy)) OR AB=(exercise)) OR AB=(resistance training)) OR AB=(progressive resistance training)) OR AB=(rehabilitation exercise)) OR AB=(aquatic exercise)) OR AB=(aerobic exercise)) OR AB=(kinesio taping)) OR AB=(decongestive lymphatic therapy)

Hits: 540331

ID: #6

Search strategy: English: #5 OR #4. Chinese note: combined the traditional Chinese medicine external/acupuncture intervention search block with the physical therapy/exercise rehabilitation intervention search block.

Hits: 853884

**Embase Search Strategy**

Database: Embase. Run date: 24 Jan 2026

ID: #1

Search strategy: English: 'breast tumor'/exp

Hits: 798,504

ID: #2

Search strategy: English: 'breast cancer':ab,ti OR 'breast tumor':ab,ti OR 'breast neoplasm':ab,ti

Hits: 591,884

ID: #3

Search strategy: English: #1 OR #2

Hits: 857,092

ID: #4

Search strategy: English: 'lymphedema'/exp

Hits: 50,474

ID: #5

Search strategy: English: 'lymphedema':ab,ti OR 'breast cancer-related lymphedema':ab,ti OR 'postmastectomy lymphedema':ab,ti

Hits: 17,528

ID: #6

Search strategy: English: #4 OR #5

Hits: 52,247

ID: #7

Search strategy: English: #3 AND #6

Hits: 8,515

ID: #8

Search strategy: English: acupuncture:ab,ti OR 'acupressure':ab,ti OR 'acupuncture analgesia':ab,ti OR 'auricular acupuncture':ab,ti OR 'catgut embedding':ab,ti OR electroacupuncture:ab,ti OR pharmacopuncture:ab,ti OR 'blood-letting puncture':ab,ti OR 'moxibustion':ab,ti OR 'needling':ab,ti OR zhenjiu:ab,ti OR meridian:ab,ti OR 'ching lo':ab,ti OR 'meridian system':ab,ti OR 'warm acupuncture':ab,ti OR 'acupuncture point':ab,ti OR 'acupoint massage':ab,ti OR 'acupoint application':ab,ti OR 'fumigation':ab,ti OR 'washing':ab,ti [ID inferred from #11; original export did not display the line number.]

Hits: 118,898

ID: #10

Search strategy: English: 'moxibustion'/exp

Hits: 5,892

ID: #11

Search strategy: English: #8 OR #9 OR #10

Hits: 136,168

ID: #12

Search strategy: English: low level laser therapy':ab,ti OR 'manual lymphatic drainage':ab,ti OR 'massage':ab,ti OR 'chinese massage':ab,ti OR 'compression sleeve':ab,ti OR 'compression therapy':ab,ti OR 'skin care':ab,ti OR 'intermittent pneumaticcompression pump':ab,ti OR 'pneumatic cuffs':ab,ti OR 'thermotherapy':ab,ti OR 'exercise':ab,ti OR 'resistance training':ab,ti OR 'progressive resistance training':ab,ti OR 'rehabilitation exercise':ab,ti OR 'aquatic exercise':ab,ti OR 'aerobic exercise':ab,ti OR 'kinesio taping':ab,ti OR 'decongestive lymphatic therapy':ab,ti [ID inferred from #14; original export did not display the line number.]

Hits: 561,086

ID: #14

Search strategy: English: #12 OR #13

Hits: 685,450

ID: #15

Search strategy: English: #11 OR #14

Hits: 811,585

ID: #16

Search strategy: English: 'randomized controlled trial':it OR 'controlled clinical trial':it OR randomized:ab,ti OR randomly:ab,ti OR 'randomized controlled trials as topic':ab,ti

Hits: 1,867,858

ID: #17

Search strategy: English: #7 AND #15 AND #16

Hits: 522

**Cochrane Library Search Strategy**

Database: Cochrane Library. Date run: 24/01/2026 11:02:49.

ID: #1

Search strategy /: English: MeSH descriptor: [Breast Neoplasms] explode all trees

Hits: 21393

ID: #2

Search strategy /: English: (breast cancer):ti,ab,kw OR (breast NEXT cancer):ti,ab,kw OR (cancer of the breast):ti,ab,kw OR (breast NEXT neoplasm):ti,ab,kw OR (breast NEXT tumor):ti,ab,kw

Hits: 50006

ID: #3

Search strategy /: English: (breast NEXT tumour):ti,ab,kw OR (breast NEXT carcinoma):ti,ab,kw OR (breast NEXT malignan):ti,ab,kw OR (mammary NEXT cancer):ti,ab,kw OR (mammary NEXT neoplasm):ti,ab,kw

Hits: 3727

ID: #4

Search strategy /: English: (mammary NEXT carcinoma):ti,ab,kw OR (mastectom):ti,ab,kw OR (lumpectom):ti,ab,kw OR (breast conserving):ti,ab,kw OR (breast-conserving):ti,ab,kw

Hits: 1995

ID: #5

Search strategy /: English: #1 OR #2 OR #3 OR #4

Hits: 51735

ID: #6

Search strategy /: English: MeSH descriptor: [Lymphedema] explode all trees

Hits: 995

ID: #7

Search strategy /: English: (lymphedema):ti,ab,kw OR (lymphoedema):ti,ab,kw OR (BCRL):ti,ab,kw OR (breast cancer-related lymphedema):ti,ab,kw OR (breast cancer lymphedema):ti,ab,kw

Hits: 2163

ID: #8

Search strategy /: English: (postmastectomy lymphedema):ti,ab,kw OR (arm lymphedema):ti,ab,kw OR (upper limb lymphedema):ti,ab,kw OR (arm swelling):ti,ab,kw OR (upper limb swelling):ti,ab,kw

Hits: 2111

ID: #9

Search strategy /: English: #6 OR #7 OR #8

Hits: 3316

ID: #10

Search strategy /: English: MeSH descriptor: [Acupressure] explode all trees

Hits: 646

ID: #11

Search strategy /: English: MeSH descriptor: [Acupuncture Therapy] explode all trees

Hits: 7410

ID: #12

Search strategy /: English: MeSH descriptor: [Electroacupuncture] explode all trees

Hits: 1243

ID: #13

Search strategy /: English: MeSH descriptor: [Moxibustion] explode all trees

Hits: 714

ID: #14

Search strategy /: English: (acupressure):ti,ab,kw OR (shiatsu):ti,ab,kw OR (acupoint massage):ti,ab,kw OR (acupoint stimulation):ti,ab,kw OR (acupuncture):ti,ab,kw

Hits: 25142

ID: #15

Search strategy /: English: (electroacupuncture):ti,ab,kw OR (moxibustion):ti,ab,kw OR (moxa):ti,ab,kw OR (TEAS):ti,ab,kw OR (transcutaneous electrical acupoint stimulation):ti,ab,kw

Hits: 7942

ID: #16

Search strategy /: English: MeSH descriptor: [Physical Therapy Modalities] explode all trees

Hits: 43151

ID: #17

Search strategy /: English: MeSH descriptor: [Exercise Therapy] explode all trees

Hits: 24080

ID: #18

Search strategy /: English: MeSH descriptor: [Massage] explode all trees

Hits: 1804

ID: #19

Search strategy /: English: (manual lymph):ti,ab,kw OR (drainage):ti,ab,kw OR (manual lymphatic drainage):ti,ab,kw OR (MLD):ti,ab,kw OR (lymphatic drainage):ti,ab,kw

Hits: 14015

ID: #20

Search strategy /: English: (lymph* drainage):ti,ab,kw OR (complex decongestive):ti,ab,kw OR (CDT):ti,ab,kw

Hits: 2812

ID: #21

Search strategy /: English: (compression):ti,ab,kw OR (compression sleeve):ti,ab,kw OR (compression garment):ti,ab,kw OR (bandag):ti,ab,kw OR (compression stocking):ti,ab,kw

Hits: 13201

ID: #22

Search strategy /: English: (compression hosiery):ti,ab,kw

Hits: 84

ID: #23

Search strategy /: English: (intermittent pneumatic compression):ti,ab,kw OR (pneumatic compression):ti,ab,kw OR (pneumatic compression device):ti,ab,kw OR (IPC):ti,ab,kw OR (intermittent pneumatic compression device):ti,ab,kw

Hits: 1624

ID: #24

Search strategy /: English: #10 OR #11 OR #12 OR #13 OR #14 OR #15

Hits: 27928

ID: #25

Search strategy /: English: #16 OR #17 OR #18 OR #19 OR #20 OR #21 OR #22 OR #23

Hits: 70839

ID: #26

Search strategy /: English: #24 OR #25

Hits: 95897

ID: #27

Search strategy /: English: #5 AND #9 AND #26

Hits: 640

**Supplementary Material S2. RoB 2.0 assessment**

**Supplementary Figure S2-1. RoB 2.0 risk-of-bias summary plot**

*[See standalone file: RoB2_summary_barplot.png/pdf]*

**Supplementary Table S2-1. RoB 2.0 study-level assessment details**

| **ID** | **Study** | **D1 Random.** | **D2 Deviat.** | **D3 Missing** | **D4 Measure.** | **D5 Select.** | **Overall** |
| --- | --- | --- | --- | --- | --- | --- | --- |
| 1 | zimmerman 2012 | Low | Low | Low | Low | Some concerns | Some concerns |
| 2 | zhang 2025 | Low | Low | Low | Low | Some concerns | Some concerns |
| 3 | torres lacomba 2010 | Low | Low | Low | Low | Low | Low |
| 4 | schmitz 2010 | Low | Low | Low | Low | Low | Low |
| 5 | ochalek 2018 | Low | Low | Low | Low | Some concerns | Some concerns |
| 6 | lin 2023 | Low | Some concerns | Low | Low | Some concerns | Some concerns |
| 7 | kas ozdemir | Low | Low | Low | Low | Some concerns | Some concerns |
| 8 | hu 2025 | Low | Low | Low | Low | Low | Low |
| 9 | donmez 2017 | Low | Low | Low | Low | Low | Low |
| 10 | devoogdt 2011 | Low | Low | Low | Low | Low | Low |
| 11 | castro-sanchez 2011 | Low | Low | Low | Some concerns | Some concerns | Some concerns |
| 12 | bundred 2023 | Low | Low | Low | Low | Some concerns | Some concerns |
| 13 | anderson 2012 | Low | Low | Low | Low | Low | Low |
| 14 | ammitzboll 2019 | Low | Low | Low | Low | Some concerns | Some concerns |
| 15 | rindner 2019 | Low | Low | Low | Low | Some concerns | Some concerns |
| 16 | sagen 2009 | Low | Low | Low | Low | Low | Low |
| 17 | fan 2026 | Low | Low | Low | Low | Low | Low |
| 18 | bruce 2022 | Low | Low | Low | Low | Some concerns | Some concerns |
| 19 | xu 2022 | Some concerns | Low | Low | Some concerns | Some concerns | Some concerns |
| 20 | xiao 2022 | Some concerns | Some concerns | Low | Low | Low | Some concerns |
| 21 | wang 2016 | Some concerns | Low | Low | Some concerns | Low | Some concerns |
| 22 | wang x 2022 | Some concerns | Low | Low | Low | Some concerns | Some concerns |
| 23 | ou ql 2023 | Some concerns | Some concerns | Low | Low | Low | Some concerns |
| 24 | lin cj 2011 | Low | Low | Low | Low | Some concerns | Some concerns |
| 25 | wan h 2019 | Low | Low | Low | Low | Some concerns | Some concerns |
| 26 | yang hj 2021 | Low | Low | Low | Low | Some concerns | Some concerns |
| 27 | zhang y 2015 | Some concerns | Some concerns | Low | Low | Low | Some concerns |
| 28 | zhuang 2025 | Low | Some concerns | Low | Low | Low | Some concerns |
| 29 | zhou j 2024 | Some concerns | Low | Low | Some concerns | Some concerns | Some concerns |
| 30 | lian ll 2016 | Low | Low | Low | Low | Some concerns | Some concerns |
| 31 | huang c 2021 | Low | Some concerns | Low | Some concerns | Some concerns | Some concerns |
| 32 | huang yp 2021 | Some concerns | Low |  | Low | Some concerns | Some concerns |
| 33 | huang m 2022 | Low | Low | Low | Low | Some concerns | Some concerns |
| 34 | wang hl 2018 | Some concerns | Low | Low | Low | Low | Some concerns |
| 35 | gao hx 2014 | Low | Low | Low | Low | Some concerns | Some concerns |
| 36 | ma l 2021 | Low | Low | Low | Low | Low | Low |
| 37 | ma xf 2013 | Some concerns | Some concerns | Low | Some concerns | Some concerns | Some concerns |
| 38 | ma yp 2024 | Some concerns | Some concerns | Low | Some concerns | Some concerns | Some concerns |
| 39 | ma zq 2018 | Some concerns | Low | Low | Low | Some concerns | Some concerns |
| 40 | gu yt 2020 | Some concerns | Some concerns | Low | Low | Low | Some concerns |
| 41 | chen l2018 | Some concerns | Some concerns | Low | Low | Some concerns | Some concerns |
| 42 | chen yq 2022 | Low | Some concerns | Low | Low | Some concerns | Some concerns |
| 43 | hao sh 2015 | Some concerns | Low | Low | Low | Some concerns | Some concerns |
| 44 | zhao yj 2024 | Low | Some concerns | Low | Low | Low | Some concerns |
| 45 | jia jy 2023 | Some concerns | Low | Low | Low | Some concerns | Some concerns |
| 46 | yuan yl 2023 | Low | Some concerns | Low | Low | Some concerns | Some concerns |
| 47 | jiang jy 2025 | Low | Low | Low | Some concerns | Low | Some concerns |
| 48 | dong y 2017 | Low | Low | Low | Low | Some concerns | Some concerns |
| 49 | mao cl2020 | Some concerns | Some concerns | Low | Low | Some concerns | Some concerns |
| 50 | luo ll 2011 | Some concerns | Some concerns | Low | Low | Some concerns | Some concerns |
| 51 | cheng h 2021 | Low | Low | Low | Low | Some concerns | Some concerns |
| 52 | cheng y2021 | Some concerns | Some concerns | Low | Low | Low | Some concerns |
| 53 | zhu cq 2014 | Some concerns | Some concerns | Some concerns | Low | Some concerns | Some concerns |
| 54 | zhu yn 2017 | Some concerns | Low | Some concerns | Low | Low | Some concerns |
| 55 | shi lf2022 | Low | Low | Some concerns | Low | Low | Some concerns |
| 56 | wang h2018 | Some concerns | Some concerns | Low | Low | Some concerns | Some concerns |
| 57 | wang qy 2021 | Some concerns | Low | Low | Low | Low | Some concerns |
| 58 | wang sq2020 | Some concerns | Low | Low | Low | Some concerns | Some concerns |
| 59 | wang cy | Some concerns | Low | Low | Low | Some concerns | Some concerns |
| 60 | wang hj 2012 | Some concerns | Low | Low | Low | Some concerns | Some concerns |
| 61 | wang s2011 | Some concerns | Some concerns | Some concerns | Low | Some concerns | Some concerns |
| 62 | wang ql2018 | Some concerns | Low | Low | Low | Some concerns | Some concerns |
| 63 | mou q2025 | Low | Low | Low | Low | Some concerns | Some concerns |
| 64 | xiong p 2017 | Low | Low | Low | Low | Some concerns | Some concerns |
| 65 | yin xl2020 | Low | Some concerns | Low | Low | Some concerns | Some concerns |
| 66 | ouyang xj 2017 | Some concerns | Low | Low | Low | Some concerns | Some concerns |
| 67 | liangxf 2020 | Low | Low | Some concerns | Low | Some concerns | Some concerns |
| 68 | lin mh2021 | Low | Low | Low | Low | Low | Low |
| 69 | yang fq2019 | Some concerns | Low | Low | Low | Low | Some concerns |
| 70 | wu yy 2020 | Low | Some concerns | Low | Low | Some concerns | Some concerns |
| 71 | li h2015 | Low | Low | Low | Low | Low | Low |
| 72 | li xj 2018 | Low | Some concerns | Low | Some concerns | Some concerns | Some concerns |
| 73 | li xh 2021 | Low | Some concerns | Low | Some concerns | Low | Some concerns |
| 74 | li n2021 | Low | Some concerns | Low | Some concerns | Low | Some concerns |
| 75 | li jb2019 | Some concerns | Some concerns | Low | Low | Some concerns | Some concerns |
| 76 | zhu yc | Low | Some concerns | Low | Low | Low | Some concerns |
| 77 | xu yd2009 | Some concerns | Low | Some concerns | Low | Some concerns | Some concerns |
| 78 | xu xj 2020 | Low | Low | Low | Low | Some concerns | Some concerns |
| 79 | zhang yw 2014 | Low | Low | Low | Low | Low | Low |
| 80 | zhang m2025 | Some concerns | Low | Low | Low | Some concerns | Some concerns |
| 81 | liao z2015 | Some concerns | Low | Low | Low | Low | Some concerns |
| 82 | zuo yq 2014 | Some concerns | Some concerns | Low | Some concerns | Some concerns | Some concerns |
| 83 | song lf 2024 | Low | Low | Some concerns | Low | Some concerns | Some concerns |
| 84 | sun lq 2009 | Some concerns | Some concerns | Low | Low | Some concerns | Some concerns |
| 85 | jiang xl2014 | Low | Low | Low | Low | Low | Low |
| 86 | yaochen sy 2023 | Low | Low | Low | Low | Some concerns | Some concerns |
| 87 | xia y2018 | Some concerns | Low | Low | Some concerns | Some concerns | Some concerns |
| 88 | zhou h 2013 | Some concerns | Low | Some concerns | Low | Low | Some concerns |
| 89 | wu q 2017 | Low | Low | Low | Low | Some concerns | Some concerns |
| 90 | zhao nx 2021 | Low | Low | Low | Some concerns | Low | Some concerns |
| 91 | wu yp2015 | Low | Low | Low | Low | Some concerns | Some concerns |
| 92 | wu bing 2022 | Low | Some concerns | Some concerns | Low | Some concerns | Some concerns |
| 93 | liu y 2016 | Some concerns | Low | Low | Low | Some concerns | Some concerns |
| 94 | liu cl 2020 | Some concerns | Low | Low | Low | Some concerns | Some concerns |
| 95 | liu q 201 | Low | Low | Low | Low | Some concerns | Some concerns |
| 96 | liu pq 2019 | Low | Some concerns | Low | Some concerns | Some concerns | Some concerns |
| 97 | liu hy 2013 | Low | Some concerns | Low | Low | Some concerns | Some concerns |
| 98 | he ym 2021 | Low | Low | Low | Low | Low | Low |
| 99 | he xm2025 | Low | Low | Low | Some concerns | Some concerns | Some concerns |
| 100 | chou xx 2018 | Low | Low | Low | Low | Some concerns | Some concerns |

**Supplementary Material S3. Intervention node codes and definitions**

| **Node** | **English/node meaning** | **Chinese meaning** | **Outcomes represented** |
| --- | --- | --- | --- |
| UC-EX | usual care/education with unsupervised exercise | Usual care/education with unsupervised exercise; reference node | BCRL, ROM, volume |
| MLD | manual lymphatic drainage | Manual lymphatic drainage | BCRL, ROM, volume |
| IPC | intermittent pneumatic compression | Intermittent pneumatic compression | BCRL, ROM |
| COMP | compression therapy | Compression therapy | BCRL, volume |
| RT | resistance training | Resistance training | BCRL, ROM, volume |
| SUP-EX | supervised exercise | Supervised exercise/structured rehabilitation training | BCRL, volume |
| MW | microwave therapy | Microwave therapy/thermotherapy | BCRL |
| AM | acupoint massage | Acupoint massage | BCRL, ROM |
| Mox | moxibustion | Moxibustion/warming moxibustion | BCRL, ROM, volume |
| ISO | isokinetic strength training | Isokinetic strength training | ROM |
| AM+IPC | acupoint massage + intermittent pneumatic compression | Acupoint massage + intermittent pneumatic compression | BCRL, ROM |
| AM+ISO | acupoint massage + isokinetic strength training | Acupoint massage + isokinetic strength training | ROM |
| AM+MW | acupoint massage + microwave therapy | Acupoint massage + microwave therapy/thermotherapy | BCRL |
| COMP+MLD | compression therapy + manual lymphatic drainage | Compression therapy + manual lymphatic drainage | BCRL, volume |
| COMP+RT | compression therapy + resistance training | Compression therapy + resistance training | BCRL, ROM, volume |
| COMP+SUP-EX | compression therapy + supervised exercise | Compression therapy + supervised exercise | BCRL, volume |
| COMP+MLD+RT | compression therapy + manual lymphatic drainage + resistance training | Compression therapy + manual lymphatic drainage + resistance training | BCRL, volume |
| COMP+Mox+MLD | compression therapy + moxibustion + manual lymphatic drainage | Compression therapy + moxibustion/warming moxibustion + manual lymphatic drainage | BCRL |
| COMP+Mox+RT | compression therapy + moxibustion + resistance training | Compression therapy + moxibustion/warming moxibustion + resistance training | BCRL |
| IPC+MLD | intermittent pneumatic compression + manual lymphatic drainage | Intermittent pneumatic compression + manual lymphatic drainage | BCRL, ROM |
| IPC+MW | intermittent pneumatic compression + microwave therapy | Intermittent pneumatic compression + microwave therapy/thermotherapy | BCRL |
| IPC+RT | intermittent pneumatic compression + resistance training | Intermittent pneumatic compression + resistance training | BCRL, ROM |
| ISO+KT | isokinetic strength training + other interventions | Isokinetic strength training + other interventions | ROM |
| MLD+KT | manual lymphatic drainage + other interventions | Manual lymphatic drainage + other interventions | ROM |
| RT+SUP-EX | resistance training + supervised exercise | Resistance training + supervised exercise | BCRL, volume |

**Supplementary Material S4. Baseline characteristics of included studies**

*56 studies included in quantitative synthesis.*

| **Study** | **Title** | **Country/region** | **Sample size** | **Original intervention description / standardized node / main-model handling** | **Main analysis time point** | **Outcomes extracted in this review** | **Notes** |
| --- | --- | --- | --- | --- | --- | --- | --- |
| Qiu Xiaoxi (2018) | Comprehensive nursing intervention for upper-limb lymphedema after breast cancer surgery | China | T/Co=39/35 | T: COMP+MLD Co: UC-EX | 6 | Circumference-based lymphedema incidence |  |
| Wan Hui (2019) | Effect of acupoint massage on upper-limb lymphedema after breast cancer surgery | China | T/Co=30/30 | Original intervention description: MLD+AM vs MLD Standardized node/main-model handling: not used as a formal node in the final main network | 3 | Circumference-based lymphedema incidence |  |
| Zimmermann (2012) | Efficacy of Manual Lymphatic Drainage in Preventing Secondary Lymphedema after Breast Cancer Surgery | Germany | T/Co=33/34 | T: MLD Co: UC-EX | 6 months | Bilateral arm volume; Vol%; BMI; WHR. |  |
| Zhang (2025) | Preventive effects of progressive resistance training of different intensities on breast cancer–related lymphedema | China | Control/low-load/moderate-to-high-load=38/38/38; completers=37/36/37 | T: RT Co: UC-EX | 6 months | Segmental water difference; extracellular water ratio; 1-kHz and 5-kHz SFBIA; grip strength. |  |
| Torres Lacomba (2010) | Effectiveness of early physiotherapy to prevent lymphoedema after surgery for breast cancer randomised, single blinded, clinical trial | Spain | T/Co=60/60; completed 12-month follow-up=59/57 (total 116) | T: MLD Co: UC-EX | 12 months | Incidence of clinically significant secondary lymphedema; arm-circumference difference; volume ratio. |  |
| Schmitz (2010) | Weight lifting for women at risk for breast cancer-related lymphedema a randomized trial | United States | T/Co=77/77; analysis set=72/75 | T: RT Co: UC-EX | 12 months | Incident BCRL; clinically defined BCRL; symptom changes. |  |
| Sagen (2009) | Physical activity for the affected limb and arm lymphedema after breast cancer surgery. A prospective, randomized controlled trial with two years follow-up | Norway | NAR/AR=104/100 | T: RT+SUP-EX Co: SUP-EX | 3 months | Voldiff; ALE incidence; pain; physical activity level. |  |
| Ochalek (2018) | Do compression sleeves reduce the incidence of arm lymphedema and improve quality of life Two-year results from a prospective randomized trial in breast cancer survivors | Poland | CG/NCG=23/22; 2-year follow-up=20/21 | T: COMP Co: UC-EX | 12 months | Change in arm volume; BCRL incidence; EORTC QLQ-C30; EORTC QLQ-BR23. |  |
| Lin (2023) | Effectiveness of three exercise programs and intensive follow-up in improving quality of life, pain, and lymphedema among breast cancer survivors a randomized, controlled 6-month trial | China | G0/G1/G2/G3=50/50/50/50; completed=48/48/49/47 | T1: SUP-EX T2: not included in this node analysis T3: RT+SUP-EX Co: UC-EX | 6 months | FACT-B;NRS;RVC. |  |
| Kaş Özdemir (2025) | The effects of early rehabilitation on physical and psychosocial functions after breast cancer surgery | Turkey | G1/G2/G3=21/21/21 | T1: SUP-EX T2: not included in this node analysis Co: UC-EX | 3 months | Change in upper-limb circumference/volume difference; shoulder function: Constant-Murley; FACT-B; fatigue. |  |
| Hu (2025) | Three-Month Functional Exercise Reduces Upper Limb Lymphedema After Breast Cancer Surgery A Prospective Randomized Controlled Trial | China | 60/60 | T: COMP+SUP-EX Co: UC-EX | 3 months | Shoulder function (Constant-Murley), BCRL incidence, FACT-B, BPNS, HADS |  |
| Donmez (2017) | The effectiveness of a clinical and home-based physical activity program and simple lymphatic drainage in the prevention of breast cancer-related lymphedema a prospective randomized controlled study | Turkey | 25/27 | Original intervention description: SUP-EX+MLD vs UC-EX Standardized node/main-model handling: not used as a formal node in the final main network | 1.5 months | Upper-limb circumference, severity of lymphedema-related symptoms, DASH, VAS | Not included in the corresponding main network, or node mapping requires manual verification. |
| Devoogdt (2011) | Effect of manual lymph drainage in addition to guidelines and exercise therapy on arm lymphoedema related to breast cancer randomised controlled trial | Belgium | 79/81 | T: MLD Co: UC-EX | 12 months | Cumulative incidence of lymphedema, time to onset, point prevalence, arm volume/circumference |  |
| Castro-Sanchez (2011) | Preventing lymphoedema after breast cancer surgery by elastic restraint orthotic and manual lymphatic drainage A randomized clinical trial | Spain | 24/24 | T: COMP+MLD Co: UC-EX | 8 months | Quality of life, body composition/extracellular water, skin temperature, shoulder function, pain, upper-limb volume |  |
| Bundred (2023) | Prevention of lymphoedema after axillary clearance by external compression sleeves PLACE randomised trial results. Effects of high BMI | United Kingdom | 74/69 | T: COMP Co: UC-EX | 12 months | Clinical lymphedema incidence, moderate lymphedema defined as RAVI >20%, QoL, effect of BMI on outcomes, cellulitis |  |
| Anderson (2012) | A randomized trial of exercise on well-being and function following breast cancer surgery The RESTORE trial | United States | 52/52 | T: COMP+SUP-EX Co: UC-EX | — | HRQoL, 6-minute walk, upper- and lower-limb strength, arm volume, physical activity |  |
| Ammitzboll (2019) | Progressive resistance training to prevent arm lymphedema in the first year after breast cancer surgery Results of a randomized controlled trial | Denmark | 82/76 | T: RT Co: UC-EX | 12 months | Arm volume, clinically relevant lymphedema, lymphedema-related symptoms/clinical criteria, tissue-composition measures |  |
| Ridner (2019) | A Randomized Trial Evaluating Bioimpedance Spectroscopy Versus Tape Measurement for the Prevention of Lymphedema Following Treatment for Breast Cancer Interim Analysis | United States/Australia multicenter | TM/BIS=245/263 (interim subset; total N=508) | Original intervention description: BIS+COMP vs TM+COMP Standardized node/main-model handling: not used as a formal node in the final main network | — | Trigger rate, progression to clinical BCRL requiring CDP, time to trigger, time to progression | Monitoring-trigger strategy; not included in the final intervention-node table in version 6, section 2.5; retained according to the original study characteristics. |
| Fan (2026) | Effects of Resistance Training at Different Intensities on Preventing Breast Cancer-Related Lymphedema A 1-Year Randomized Controlled Trial | China | CG/L-MIEG/M-HIEG=38/38/38(completed37/36/37) | T: RT Co: UC-EX | 12 months | BCRL incidence, circumference difference, ECW, SFBIA (1/5 kHz), grip strength, adherence/adverse events |  |
| Bruce (2022) | Exercise to prevent shoulder problems after breast cancer surgery the PROSPER RCT | United Kingdom | 196/196 (191/191 after treatment allocation; baseline questionnaires 175/175) | T: SUP-EX Co: UC-EX | — | DASH, pain (NRS/DN4/FACT-B4), wound complications, LBCQ lymphedema symptoms, SF-12, EQ-5D-5L, resource use |  |
| Xu Lingling (2022) | Application of acupoint massage combined with upper-limb rehabilitation training for affected-limb functional exercise after modified radical mastectomy for breast cancer | China | 85/85(completed84/81) | T: IPC+AM Co: IPC | 3 months | Shoulder abduction/forward flexion/extension, lymphedema incidence, FACT-B |  |
| Xiao Zhiping (2022) | Effect of isokinetic strength training combined with kinesiology taping on radiotherapy-related adverse effects after radical mastectomy for breast cancer | China | 32/32 | T: ISO+OTHERS Co: UC-EX | 1.17 months | Shoulder ROM, DASH, lymphedema incidence, radiation-induced skin injury |  |
| Wang Xianqing (2016) | Effect of early numerically controlled pneumatic therapy for preventing affected-limb edema after breast cancer surgery | China | 30/30 | T: IPC Co: UC-EX | 0.5 months | Change in affected-limb circumference; incidence and severity of lymphedema on postoperative day 15 |  |
| Wang Xuan (2022) | Effects of manual lymphatic drainage and rehabilitation training based on the concept of hand-function rehabilitation on limb lymphedema and quality of life after breast cancer surgery | China | 80/80 | T: MLD Co: UC-EX | 6 months | Lymphedema incidence and grade, AWS incidence, shoulder-joint function, hand function, quality of life |  |
| Ou Qinglian (2023) | Effect of manual lymphatic drainage in promoting limb functional recovery after breast cancer surgery | China | 40/40 | T: MLD Co: UC-EX | 3 months | Pain score, excellent/good rate of shoulder-joint function, lymphedema incidence and stage |  |
| Lin Caijuan (2011) | Clinical study of the effect of exercise training on quality of life after breast cancer surgery | China | 80/76 | T: SUP-EX Co: UC-EX | 1 months | Incidence of ipsilateral upper-limb edema; SF-36 quality of life |  |
| Yang Haijun (2021) | Effect of progressive resistance-training nursing intervention for preventing lymphedema after breast cancer surgery | China | 50/50 | T: RT Co: UC-EX | — | Shoulder range of motion, lymphedema incidence, upper-limb volume difference |  |
| Zhang Yin (2015) | Cause analysis, prevention, and treatment of upper-limb edema after modified radical mastectomy for breast cancer | China | 70/70 | T: MLD+OTHERS Co: UC-EX | 2 months | Postoperative pain grade, severity of upper-limb edema, time from upper-limb rehabilitation training to vertical arm elevation |  |
| Zhuang Yuhong (2025) | Application of early manual lymphatic drainage combined with resistance exercise for preventing lymphedema after breast cancer surgery | China | 34/34 | T: MLD+RT Co: UC-EX | — | Mean bilateral upper-limb circumference difference, BCLE-SEI, FACT-B |  |
| Zhou Jie (2024) | Analysis of psychological support combined with progressive resistance training on psychological status and risk of upper-limb lymphedema after radical mastectomy for breast cancer | China | 35/35 | T: RT Co: UC-EX | 3 months | SAS, SDS, upper-limb lymphedema incidence, nursing satisfaction |  |
| Lian Lingling (2016) | Effect of massage combined with an air-wave pressure therapy device for preventing lymphedema after modified radical mastectomy for breast cancer | China | 55/55/55 | T1: IPC+MLD T2: MLD Co: UC-EX | 6 months | Lymphedema incidence and severity; functional recovery of the affected limb (abduction/rotation/elevation) |  |
| Huang Cheng (2021) | Effect of massage combined with an air-wave pressure therapy device for preventing lymphedema after modified radical mastectomy for breast cancer | China | 36/36 | T: AM+IPC Co: AM | 3 months | — |  |
| Huang Yuping (2021) | Effects of progressive rehabilitation training on lymphedema and affected-limb range of motion after radical mastectomy for breast cancer | China | 43/43 | T: SUP-EX Co: UC-EX | 3 months | Affected-limb range-of-motion score, affected-limb muscle strength, affected-limb lymphedema |  |
| Huang Mei (2022) | Role of a gradient pressure therapy device combined with anticipatory nursing for affected-limb lymphedema after breast cancer surgery | China | 60/60 | T: IPC+AM Co: AM | 1 months | Affected-limb lymphedema incidence, shoulder ROM, EORTC QLQ-C30 quality of life |  |
| Wang Hongli (2018) | Effect of manual lymphatic drainage for preventing upper-limb lymphedema after breast cancer surgery | China | 50/40 | T: MLD Co: UC-EX | 6 months | Lymphedema incidence, AWS incidence |  |
| Gao Haixia (2014) | Nursing intervention for postoperative affected-limb lymphedema in patients with breast cancer | China | 30/30 | T: IPC Co: UC-EX | 3 months | Ipsilateral upper-limb edema incidence and severity |  |
| Ma Li (2021) | Analysis of the preventive effect of manual lymphatic drainage combined with upper-limb functional exercise on upper-limb lymphedema after breast cancer surgery | China | 40/40 | T: MLD Co: UC-EX | 12 months | Upper-limb lymphedema incidence, severity, and intervention effect |  |
| Ma Xiufen (2013) | Clinical study of manual lymph drainage combined with air-wave pressure therapy for postoperative affected-limb rehabilitation in patients with breast cancer | China | 62/58 | T: IPC+MLD Co: UC-EX | 2 months | Upper-limb lymphedema incidence and severity, shoulder ROM, affected-limb muscle strength, pain score |  |
| Ma Yuping (2024) | Analysis of progressive resistance-training nursing for preventing lymphedema after breast cancer surgery | China | 44/44 | T: COMP+RT Co: UC-EX | — | Lymphedema incidence, lymphedema severity, nursing satisfaction |  |
| Ma Zhiqiang (2018) | Preventive effect of a pneumatic pump combined with microwave therapy on upper-limb lymphedema after modified radical mastectomy for breast cancer | China | 103/103(analysis99/96) | T: IPC+ MW Co: UC-EX | 6 months | Lymphedema incidence at 6 months and 1 year after surgery; analysis of influencing factors |  |
| Gu Yetong (2020) | Effect of manual lymphatic drainage on upper-limb lymphedema after radical mastectomy for breast cancer | China | 84/84 | T: MLD Co: UC-EX | 1 months | Incidence of upper-limb lymphedema on the surgical side |  |
| Chen Li (2018) | Effect of an air-wave pressure therapy device in 60 cases of ipsilateral upper-limb lymphedema after breast cancer surgery | China | 30/30 | T: IPC Co: UC-EX | 1 months | Ipsilateral upper-limb edema incidence and severity |  |
| Chen Yuqin (2022) | Preventive effect of manual lymphatic drainage combined with pneumatic therapy on upper-limb lymphedema after breast cancer surgery | China | 40/40 | T: IPC+MLD Co: UC-EX | 1 months | Upper-limb lymphedema incidence at 1 and 4 weeks after surgery, edema severity, self-efficacy level |  |
| Hao Suhong (2015) | Clinical study of tuina therapy for preventing and treating upper-limb lymphedema after breast cancer surgery | China | 36/36 | T: AM Co: UC-EX | 3 months | Lymphedema incidence; excellent recovery rate of ipsilateral upper-limb function |  |
| Zhao Yajing (2024) | Value of resistance training combined with staged health education for preventing lymphedema after breast cancer surgery | China | 52/52 | T: RT Co: UC-EX | 6 months | Postoperative lymphedema incidence, FACT-B quality of life |  |
| Jia Jingyi (2023) | Effect of isokinetic strength training combined with acupoint massage on upper-limb lymphedema and limb function after breast cancer surgery | China | 41/41 | T: ISO+AM Co: UC-EX | 1 months | DASH score, grip strength, FACT-B score, shoulder ROM, upper-limb lymphedema incidence |  |
| Yuan Yulian (2023) | Clinical study of copper bian-stone gua sha combined with manual lymphatic drainage for preventing breast cancer-related lymphedema | China | 30/30 | Original intervention description: MLD+TCM vs UC-EX Standardized node/main-model handling: not used as a formal node in the final main network | 0.93 months | Lymphedema severity, EORTC QLQ-C30, EORTC QLQ-BR23 | Not included in the corresponding main network, or node mapping requires manual verification. |
| Jiang Jinying (2025) | Analysis of whole-process management of hand-function exercise after breast cancer surgery | China | 150/150 | T: SUP-EX Co: UC-EX | — | Satisfaction, DASH score, FMA score, SAS score, SDS score, FACT-B score, lymphedema incidence |  |
| Dong Yan (2017) | Effect of an integrated Chinese-Western medicine "three-in-one" intervention for preventing ipsilateral upper-limb edema after breast cancer surgery | China | 50/50 | T: AM+Mox Co: UC-EX | 0.5 months | Affected-limb edema incidence, edema severity, numbness/swelling sensation of the affected limb |  |
| Mao Chuanlan (2020) | Evaluation of meridian moxibustion for preventing and treating ipsilateral upper-limb edema after radical mastectomy for breast cancer | China | 75/75 | T: Mox Co: UC-EX | 1 months | Upper-limb edema incidence, edema severity, comfort score |  |
| Luo Lingling (2021) | Effect of psychological intervention combined with air-pressure wave therapy on upper-limb lymphedema after breast cancer surgery | China | 55/55 | T: IPC Co: UC-EX | 6 months | Lymphedema incidence at 6 and 12 months after surgery, edema severity, anxiety/depression scores, satisfaction rate, quality of life |  |
| Cheng Hong (2021) | Effect of an air-wave pressure therapy device combined with progressive resistance training on postoperative upper-limb function and lymphedema incidence in patients with breast cancer | China | 43/43 | T: IPC+RT Co: UC-EX | 3 months | Affected-limb elevation height, rotation angle, abduction angle, DASH score, lymphedema severity |  |
| Cheng Yi (2021) | Effect of postoperative gradient pressure therapy on lymphedema in patients with breast cancer | China | 43/43 | T: IPC Co: UC-EX | — | Lymphedema severity; shoulder adduction/abduction/elevation/extension ROM |  |
| Chu Yanqing (2014) | Effect of air-wave pressure therapy combined with rehabilitation training on upper-limb functional recovery after radical mastectomy for breast cancer | China | 40/40 | T: IPC Co: UC-EX | 6 months | VAS score, Constant-Murley score, shoulder ROM, rehabilitation-training adherence, complications |  |
| Zhu Yanan (2017) | Study of traditional Chinese medicine nursing techniques for reducing postoperative complications in patients with breast cancer | China | 100/100 (analyzed 94/92) | T: AM+Mox Co: UC-EX | 0.25 months | Arm circumference, accompanying symptoms of the affected limb, Athens Insomnia Scale, shoulder ROM, subcutaneous effusion, skin-flap necrosis, affected-limb edema, sleep disturbance, shoulder-joint dysfunction |  |
| Shi Lingfang (2022) | Application value of kinesiology taping combined with tai chi exercise in postoperative rehabilitation after breast cancer surgery | China | 48/48 | T: OTHERS Co: UC-EX | 6 months | Ipsilateral upper-limb edema incidence, shoulder ROM, bilateral upper-arm circumference difference, upper-limb lymph flow, FACT-B score, Constant-Murley score |  |
| Wang Hong (2018) | Application value of different nursing methods for hand-foot syndrome and lymphedema in patients with breast cancer | China | A/B/C=40/40/40 | T1: IPC+MLD T2: MLD Co: UC-EX | 1 months | Upper-limb edema incidence; excellent/good rate for affected-limb elevation/abduction/rotation function; hand-foot syndrome grade |  |
| Wang Qinyu (2021) | Effect of a pressure-wave therapy device combined with functional rehabilitation exercise on upper-limb lymphedema and rehabilitation after breast cancer surgery | China | 44/44 | T: IPC+MLD Co: MLD | 1 months | Upper-limb lymphedema severity; shoulder adduction/abduction/extension/flexion ROM |  |
| Wang Shuqing (2020) | Effect of TDP irradiation combined with finger acupoint stimulation for preventing lymphedema after breast cancer surgery | China | Control/A/B=30/30/30 | T1: MW T2: AM+MW Co: UC-EX | 3 months | Upper-limb edema incidence, edema severity |  |
| Wang Chunying (2019) | Analysis of the effect of manual lymphatic drainage for preventing postoperative lymphedema in patients with breast cancer | China | 100/100 | T: MLD Co: UC-EX | 6 months | Lymphedema incidence at different postoperative time points |  |
| Wang Huijing (2012) | Clinical observation of early moxibustion intervention for promoting affected-limb functional recovery after breast cancer surgery | China | 30/30 | T: Mox Co: UC-EX | 2 months | Shoulder ROM, grip-strength index, upper-limb lymphedema, satisfaction with affected-limb functional recovery |  |
| Wang Shuai (2011) | Application of an air-wave pressure therapy device for preventing affected-limb lymphedema after breast cancer surgery | China | 60/60 | T: IPC Co: UC-EX | 1 months | Change in affected-limb circumference, lymphedema incidence, edema severity |  |
| Wang Qianlin (2018) | Application of comprehensive lymphedema treatment after breast cancer surgery | China | 140/140 | T: IPC+MLD Co: UC-EX | 6 months | Self-care ability score, bilateral upper-limb circumference difference, lymphedema incidence at 1/3/6/12 months after surgery, edema severity at 12 months after surgery |  |
| Mou Qing (2025) | Application value of progressive resistance training combined with lymphatic drainage for preventing upper-limb edema after breast cancer surgery | China | 45/45 | T: COMP+MLD+RT Co: MLD | 12 months | Upper-limb lymphedema incidence at 12 months after surgery, swelling severity, DASH score, FACT-B score |  |
| Xiong Ping (2017) | Effect of nursing intervention for preventing lymphedema after breast cancer surgery | China | 60/60 | T: IPC+MLD Co: UC-EX | 12 months | Lymphedema incidence, edema severity |  |
| Yin Xiuling (2020) | Effects of different nursing interventions on upper-limb lymphedema after breast cancer surgery | China | usual care/massage/compression=42/42/42 | T1: MLD T2: IPC+MLD Co: UC-EX | 9 months | Affected-limb edema severity; recovery of shoulder elevation/abduction/rotation function |  |
| Ouyang Xiangjun (2017) | Application of a pressure therapy device in patients with lymphedema after breast cancer surgery | China | 46/46 | T: IPC+MLD Co: UC-EX | 6 months | Lymphedema severity; upper-limb range of motion (elevation/rotation/abduction) |  |
| Liang Xuefang (2020) | Study of a pneumatic pump combined with manual massage for preventing upper-limb lymphedema after breast cancer surgery | China | 71/142 (the main text states "213 patients were randomly allocated equally") | T: IPC+MLD Co: UC-EX | 6 months | Upper-limb lymphedema incidence |  |
| Lin Meihong (2021) | Effect of air-pressure wave therapy combined with functional rehabilitation training on lymphedema and limb function after modified radical mastectomy for breast cancer | China | 40/40 | T: IPC Co: UC-EX | 6 months | Lymphedema incidence at 3/6/12 months after surgery, Constant-Murley shoulder-joint score |  |
| Yang Fuquan (2019) | Effect of microwave therapy for preventing upper-limb lymphedema after modified radical mastectomy for breast cancer | China | 43/43 | T: MW Co: UC-EX | 12 months | Occurrence of upper-limb lymphedema, VAS score, excellent/good rate of upper-limb functional recovery |  |
| Wu Yuyun (2020) | Effect of progressive resistance-training nursing intervention for preventing lymphedema after breast cancer surgery | China | 34/34 | T: COMP+RT Co: UC-EX | 3 months | Lymphedema severity, upper-arm volume difference, shoulder ROM |  |
| Li He (2015) | Study of postoperative progressive resistance training for promoting rehabilitation in patients with breast cancer | China | 33/33 (final completers 30/30) | T: RT Co: UC-EX | 3 months | Grip strength, shoulder ROM, bilateral arm circumference/lymphedema occurrence, FACT-B, PTGI |  |
| Li Xiaojin (2018) | Effect of manual lymphatic drainage combined with functional exercise on quality of life after breast cancer surgery | China | 57/59 | T: MLD Co: UC-EX | 3 months | Pain NRS, lymphedema occurrence, affected-limb functional status, quality of life (functional dimensions of the EORTC QLQ-C30) |  |
| Li Xiaohua (2021) | Preventive nursing effect of warm acupuncture combined with progressive resistance training for upper-limb lymphedema after radical mastectomy for breast cancer | China | 51/51 | T: COMP+Mox+RT Co: COMP+RT | 12 months | Grip strength, shoulder ROM ratio, FACT-B, efficacy index at 10 cm above the elbow, lymphedema incidence and swelling severity at 1 year after surgery, DASH, FMA |  |
| Li Na (2021) | Effect of gradient pressure therapy on postoperative lymphedema, limb functional rehabilitation, and quality of life in patients with breast cancer | China | 43/42 | T: IPC Co: UC-EX | 3 months | Lymphedema incidence, shoulder ROM, FACT-B |  |
| Li Jiabao (2019) | Effect of progressive resistance training for preventing upper-limb lymphedema after breast cancer surgery | China | 736/736 | T: COMP+RT Co: UC-EX | 12 months | Upper-limb lymphedema occurrence at 12 months; DASH upper-limb disability symptoms |  |
| Zhu Yongcui (2021) | Effect of manual lymphatic drainage combined with functional rehabilitation exercise on affected-limb functional recovery after breast cancer surgery | China | 30/30 | T: MLD Co: UC-EX | 3 months | Lymphedema severity, pain NRS, affected-limb flexion/abduction/internal and external rotation/extension angles, anterior/lateral wall-climbing height |  |
| Xu Youdi (2009) | Effect of a gradient pressure therapy device for preventing affected-limb lymphedema after breast cancer surgery | China | 50/50 | T: IPC Co: UC-EX | 1.25 months | Change in affected-limb circumference, lymphedema incidence, shoulder ROM |  |
| Xu Xijuan (2020) | Effect of self-lymphatic drainage for preventing lymphedema after breast cancer surgery | China | 60/60 | T: MLD Co: UC-EX | 6 months | Increase in ipsilateral upper-limb volume at different time points, lymphedema incidence |  |
| Zhang Yuwei (2014) | Effect of thermotherapy combined with manual therapy on affected-limb dysfunction after breast cancer surgery | China | 35/35 | T: MW Co: UC-EX | 6 months | Upper-limb edema, subcutaneous effusion, skin-flap necrosis, upper-limb numbness with restricted activity, ipsilateral muscle strength, abduction/elevation/rotation function |  |
| Zhang Shumin (2025) | Clinical effect of mild moxibustion for preventing breast cancer-related lymphedema | China | 44/44 (final analysis 42/43) | T: COMP+Mox+MLD Co: COMP+MLD | 6 months | Six-point arm-circumference measurements, local tissue water ratio, BCLE-SEI, FACT-B, lymphedema incidence |  |
| Liao Zhen (2015) | Nursing effect of compression nursing intervention for affected-limb lymphedema after breast cancer surgery | China | 40/40 | T: COMP Co: UC-EX | 3 months | Incidence and severity of ipsilateral upper-limb edema at 12 weeks after surgery |  |
| Zuo Yaqin (2014) | Application of acupoint massage for operative-limb functional rehabilitation after modified radical mastectomy for breast cancer | China | 30/30 | T: AM Co: UC-EX | — | Shoulder-joint function score, operative-limb volume |  |
| Song Lifang (2024) | Analysis of the preventive effect of manual lymphatic drainage plus upper-limb functional exercise on upper-limb lymphedema after breast cancer surgery | China | 30/30 | T: MLD Co: UC-EX | — | Upper-limb lymphedema status, treatment effect, bilateral upper-arm circumference difference, upper-limb lymph flow, upper-limb function, quality-of-life score |  |
| Sun Lianqin (2009) | Effect of functional exercise methods for the ipsilateral upper limb after radical mastectomy for breast cancer | China | 50/50 | T: SUP-EX Co: UC-EX | 0.33 months | Postoperative elevation gap, backward-extension gap, subcutaneous effusion, primary wound healing, ipsilateral upper-arm edema, activity target achievement, affected-limb functional recovery |  |
| Jiang Xiaoling (2014) | Effect of ipsilateral upper-limb compression nursing on affected-limb lymphedema after breast cancer surgery | China | 45/45 | T: COMP Co: UC-EX | 3 months | Incidence and severity of upper-limb edema at 3 months after surgery |  |
| Yaochen Siyu (2023) | Effect of early manual lymphatic drainage combined with compression sleeves for preventing breast cancer-related lymphedema | China | Control/MLD/MLD+sleeve=38/38/38 (completed 38/36/35) | T1: MLD T2: COMP+MLD Co: UC-EX | 3 months | BCRL incidence at 1 and 3 months after surgery; breast cancer lymphedema symptom index at 3 months after surgery; quality of life at 3 months after surgery |  |
| Xia Ying (2018) | Effect of functional rehabilitation exercise combined with adjunctive gradient pressure therapy on postoperative recovery in patients with breast cancer | China | 40/40 | T: IPC Co: UC-EX | 3 months | Lymphedema incidence at postoperative weeks 4 and 12; shoulder ROM (extension, forward flexion/elevation, abduction, adduction) |  |
| Zhou Hong (2013) | Effect of moxibustion intervention for preventing affected-limb lymphedema after breast cancer surgery | China | 40/40 | T: AM+Mox Co: UC-EX | — | Circumference differences at six measurement points |  |
| Wu Jing (2017) | Acupoint massage combined with manual lymphatic drainage for preventing affected-limb lymphedema after breast cancer surgery | China | 20/20 | Original intervention description: AM+MoxLD vs UC-EX Standardized node/main-model handling: not used as a formal node in the final main network | — | Operative-limb circumference (10 cm below the elbow, 10 cm above the elbow, 15 cm above the elbow), operative-limb volume | Not included in the corresponding main network, or node mapping requires manual verification. |
| Zhao Nixia (2021) | Effect of resistance training combined with targeted nursing on upper-limb lymphedema and upper-limb function after breast cancer surgery | China | 41/41 | T: COMP+MLD+RT Co: COMP+MLD | — | Upper-limb infection incidence, upper-arm circumference difference, upper-limb volume difference, upper-limb lymph flow, excellent/good rate of upper-limb function, FACT-B |  |
| Wu Yuping (2015) | Effect of an air-wave therapy device for preventing and treating upper-limb lymphedema after radical mastectomy for breast cancer | China | 50/50 | T: IPC Co: UC-EX | 0.7 months | Anxiety score (SAS), lymphedema severity, lymphedema incidence |  |
| Wu Bing (2022) | Effect of enhanced recovery training combined with air-pressure wave therapy on upper-limb function after breast cancer surgery | China | 60/60 | T: IPC Co: UC-EX | 1 months | Time to awakening from anesthesia, time to first ambulation, length of hospital stay, shoulder ROM (flexion/extension/abduction/adduction), Constant-Murley score, complications (upper-limb edema, pulmonary infection, subcutaneous effusion, urinary retention, skin-flap necrosis) |  |
| Liu Yu (2016) | Preventive value of moxibustion for upper-limb lymphedema after breast cancer surgery and nursing experience | China | 54/46 | T: Mox Co: UC-EX | 3 months | Lymphedema incidence and grade; swelling volume of the affected limb |  |
| Liu Chunling (2020) | Effect of manual massage combined with a positioning cushion for preventing lymphedema after radical mastectomy for breast cancer | China | 22/20 | T: MLD Co: UC-EX | 1 months | Bilateral upper-arm circumference difference, VAS, incidence of mild-or-worse lymphedema, FACT-B |  |
| Liu Qing (2021) | Effect of an air-pressure wave therapy device combined with a solution-focused nursing model on ipsilateral upper-limb lymphedema after breast cancer surgery | China | 44/44 | T: IPC+MLD Co: MLD | 3 months | Upper-limb lymphedema severity at 3 months after surgery; elevation/rotation/abduction range of motion |  |
| Liu Pinqian (2019) | Application of an air-pressure wave therapy device in nursing care for ipsilateral upper-limb lymphedema after breast cancer surgery | China | 39/39 | T: IPC Co: UC-EX | — | Upper-limb lymphedema incidence and grade; circumference at the lower edge of the deltoid/above the elbow/below the elbow/above the wrist |  |
| Liu Huayu (2013) | Study of moxibustion at acupoints combined with massage for preventing ipsilateral upper-limb lymphedema after breast cancer surgery | China | 40/40 | T: Mox+AM Co: UC-EX | 2 months | Five-point arm circumference measurements; postoperative upper-limb edema occurrence |  |
| He Yuanmei (2021) | Effect of meridian-based acupoint massage for preventing lymphedema after breast cancer surgery | China | 60/60 | T: AM Co: UC-EX | — | Continuous upper-limb circumference, Breast Cancer Lymphedema Symptom Experience Index, VAS |  |
| He Ximei (2025) | Effect of manual lymphatic drainage combined with kinesiology taping on postoperative rehabilitation level in patients with breast cancer | China | 65/65 | T: MLD+OTHERS Co: UC-EX | 1 months | Shoulder ROM, FACT-B, Constant-Murley, 6-minute walking distance, duration of maximum heart rate, heart-rate recovery time, Gunnar Borg score, upper-limb edema incidence |  |
| **No.** | **Study** | **Country/region** | **Original sample-size description** | **Included-outcome summary** | **Notes** |  |  |
| 1 | Zimmermann (2012) | Germany | T/Co=33/34 | Volume | Volume:retained outside the window |  |  |
| 2 | Torres Lacomba (2010) | Spain | T/Co=60/60; completed 12-month follow-up=59/57 (total 116) | BCRL |  |  |  |
| 3 | Schmitz (2010) | United States | T/Co=77/77; analysis set=72/75 | BCRL |  |  |  |
| 4 | Sagen (2009) | Norway | NAR/AR=104/100 | BCRL, volume | Volume:retained outside the window; volume: unclear time point; includes an extremely sparse node |  |  |
| 5 | Ochalek (2018) | Poland | CG/NCG=23/22; 2-year follow-up=20/21 | BCRL, volume | Volume:retained outside the window; volume: includes an extremely sparse node; converted SD |  |  |
| 6 | Lin (2023) | China | G0/G1/G2/G3=50/50/50/50; completed=48/48/49/47 | BCRL |  |  |  |
| 7 | Kaş Özdemir (2025) | Turkey | G1/G2/G3=21/21/21 | Volume |  |  |  |
| 8 | Hu (2025) | China | 60/60 | BCRL |  |  |  |
| 9 | Devoogdt (2011) | Belgium | 79/81 | BCRL, volume |  |  |  |
| 10 | Castro-Sanchez (2011) | Spain | 24/24 | BCRL, volume | Volume:converted SD |  |  |
| 11 | Bundred (2023) | United Kingdom | 74/69 | BCRL |  |  |  |
| 12 | Anderson (2012) | United States | 52/52 | Volume | Volume:retained outside the window; volume: includes an extremely sparse node |  |  |
| 13 | Ammitzboll (2019) | Denmark | 82/76 | BCRL |  |  |  |
| 14 | Fan (2026) | China | CG/L-MIEG/M-HIEG=38/38/38(completed37/36/37) | BCRL |  |  |  |
| 15 | Xu Lingling (2022) | China | 85/85(completed84/81) | BCRL, ROM |  |  |  |
| 16 | Xiao Zhiping (2022) | China | 32/32 | ROM | ROM:<3 months, supplementary |  |  |
| 17 | Wang Xuan (2022) | China | 80/80 | BCRL |  |  |  |
| 18 | Ou Qinglian (2023) | China | 40/40 | BCRL |  |  |  |
| 19 | Yang Haijun (2021) | China | 50/50 | ROM, volume | ROM:retained outside the window; volume: retained outside the window; volume: unclear time point; includes an extremely sparse node |  |  |
| 20 | Zhou Jie (2024) | China | 35/35 | BCRL |  |  |  |
| 21 | Lian Lingling (2016) | China | massage/combined/usual care=55/55/55 | BCRL |  |  |  |
| 22 | Huang Cheng (2021) | China | 36/36 | BCRL |  |  |  |
| 23 | Huang Yuping (2021) | China | 43/43 | BCRL |  |  |  |
| 24 | Huang Mei (2022) | China | 60/60 | ROM | ROM:<3 months, supplementary |  |  |
| 25 | Wang Hongli (2018) | China | 50/40 | BCRL |  |  |  |
| 26 | Gao Haixia (2014) | China | 30/30 | BCRL |  |  |  |
| 27 | Ma Li (2021) | China | 40/40 | BCRL |  |  |  |
| 28 | Ma Xiufen (2013) | China | 62/58 | ROM | ROM:retained outside the window |  |  |
| 29 | Ma Zhiqiang (2018) | China | 103/103(analysis99/96) | BCRL |  |  |  |
| 30 | Hao Suhong (2015) | China | 36/36 | BCRL |  |  |  |
| 31 | Zhao Yajing (2024) | China | 52/52 | BCRL |  |  |  |
| 32 | Jia Jingyi (2023) | China | 41/41 | ROM | ROM:<3 months, supplementary |  |  |
| 33 | Luo Lingling (2021) | China | 55/55 | BCRL |  |  |  |
| 34 | Cheng Hong (2021) | China | 43/43 | BCRL, ROM |  |  |  |
| 35 | Cheng Yi (2021) | China | 43/43 | ROM | ROM:retained outside the window |  |  |
| 36 | Chu Yanqing (2014) | China | 40/40 | BCRL |  |  |  |
| 37 | Shi Lingfang (2022) | China | 48/48 | BCRL, ROM |  |  |  |
| 38 | Wang Qinyu (2021) | China | 44/44 | ROM | ROM:<3 months, supplementary |  |  |
| 39 | Wang Shuqing (2020) | China | Control/A/B=30/30/30 | BCRL |  |  |  |
| 40 | Wang Chunying (2019) | China | 100/100 | BCRL |  |  |  |
| 41 | Wang Huijing (2012) | China | 30/30 | ROM | ROM:<3 months, supplementary |  |  |
| 42 | Wang Qianlin (2018) | China | 140/140 | BCRL |  |  |  |
| 43 | Mou Qing (2025) | China | 45/45 | BCRL |  |  |  |
| 44 | Xiong Ping (2017) | China | 60/60 | BCRL |  |  |  |
| 45 | Yin Xiuling (2020) | China | usual care/massage/compression=42/42/42 | BCRL |  |  |  |
| 46 | Ouyang Xiangjun (2017) | China | 46/46 | BCRL |  |  |  |
| 47 | Liang Xuefang (2020) | China | 71/142 (the main text states "213 patients were randomly allocated equally," inconsistent with the analysis sample size) | BCRL |  |  |  |
| 48 | Lin Meihong (2021) | China | 40/40 | BCRL |  |  |  |
| 49 | Yang Fuquan (2019) | China | 43/43 | BCRL |  |  |  |
| 50 | Wu Yuyun (2020) | China | 34/34 | BCRL, ROM, volume | Volume:includes an extremely sparse node |  |  |
| 51 | Li He (2015) | China | 33/33 (final completers 30/30) | BCRL | BCRL:double-zero-event study; included in the descriptive network but excluded from the default RR main model |  |  |
| 52 | Li Xiaojin (2018) | China | 57/59 | BCRL |  |  |  |
| 53 | Li Xiaohua (2021) | China | 51/51 | BCRL |  |  |  |
| 54 | Li Na (2021) | China | 43/42 | BCRL, ROM |  |  |  |
| 55 | Li Jiabao (2019) | China | 736/736 | BCRL |  |  |  |
| 56 | Zhu Yongcui (2021) | China | 30/30 | BCRL |  |  |  |
| 57 | Xu Youdi (2009) | China | 50/50 | ROM | ROM:<3 months, supplementary |  |  |
| 58 | Xu Xijuan (2020) | China | 60/60 | BCRL, volume |  |  |  |
| 59 | Zhang Yuwei (2014) | China | 35/35 | BCRL |  |  |  |
| 60 | Zhang Shumin (2025) | China | 44/44 (final analysis 42/43) | BCRL |  |  |  |
| 61 | Liao Zhen (2015) | China | 40/40 | BCRL |  |  |  |
| 62 | Jiang Xiaoling (2014) | China | 45/45 | BCRL |  |  |  |
| 63 | Yaochen Siyu (2023) | China | Control/MLD/MLD+sleeve=38/38/38 (completed 38/36/35) | BCRL |  |  |  |
| 64 | Xia Ying (2018) | China | 40/40 | BCRL, ROM |  |  |  |
| 65 | Zhao Nixia (2021) | China | 41/41 | Volume | Volume:includes an extremely sparse node |  |  |
| 66 | Wu Bing (2022) | China | 60/60 | ROM | ROM:<3 months, supplementary |  |  |
| 67 | Liu Yu (2016) | China | 54/46 | BCRL, volume | Volume:retained outside the window; volume: includes an extremely sparse node |  |  |
| 68 | Liu Qing (2021) | China | 44/44 | BCRL, ROM | ROM:CHECK: record note says abduction ROM difference |  |  |
| 69 | He Ximei (2025) | China | 65/65 | ROM | ROM:<3 months, supplementary |  |  |
| **No.** | **Study ID** | **Study** | **Quantitative-analysis sample size** | **Original intervention description** | **Node/comparison** | **Time point (months)** | **Notes** |
| 1 | BCRL:004 | Torres Lacomba (2010) | BCRL:116 | BCRL: T: MLD; Co: UC-EX | MLD / UC-EX | 12 |  |
| 2 | BCRL:006 | Schmitz (2010) | BCRL:147 | BCRL: T: RT; Co: UC-EX | RT / UC-EX | 12 |  |
| 3 | BCRL:007 | Sagen (2009) | BCRL:204 | BCRL: T: RT+SUP-EX; Co: SUP-EX | RT+SUP-EX / SUP-EX | 3 | Volume:retained outside the window; volume: unclear time point; includes an extremely sparse node |
| 4 | BCRL:008 | Ochalek (2018) | BCRL:41 | BCRL: T: COMP; Co: UC-EX | COMP / UC-EX | 12 | Volume:retained outside the window; volume: includes an extremely sparse node; converted SD |
| 5 | BCRL:009 | Lin (2023) | BCRL:143 | BCRL: T1: SUP-EX; T2: AE+SUP-EX; T3: RT+SUP-EX; Co: UC-EX | SUP-EX / RT+SUP-EX / UC-EX | 6 |  |
| 6 | BCRL:011 | Hu (2025) | BCRL:120 | BCRL: T: COMP+SUP-EX; Co: UC-EX | COMP+SUP-EX / UC-EX | 3 |  |
| 7 | BCRL:013 | Devoogdt (2011) | BCRL:154 | BCRL: T: MLD; Co: UC-EX | MLD / UC-EX | 12 |  |
| 8 | BCRL:014 | Castro-Sanchez (2011) | BCRL:48 | BCRL: T: COMP+MLD; Co: UC-EX | COMP+MLD / UC-EX | 8 | Volume:converted SD |
| 9 | BCRL:015 | Bundred (2023) | BCRL:132 | BCRL: T: COMP; Co: UC-EX | COMP / UC-EX | 12 |  |
| 10 | BCRL:018 | Ammitzboll (2019) | BCRL:158 | BCRL: T: RT; Co: UC-EX | RT / UC-EX | 12 |  |
| 11 | BCRL:023 | Fan (2026) | BCRL:73 | BCRL: T1: RT(L-MIEG); T2: RT(M-HIEG); Co: UC-EX | RT / UC-EX | 12 |  |
| 12 | BCRL:026 | Xu Lingling (2022) | BCRL:165 | BCRL: T: AM+IPC; Co: IPC | AM+IPC / IPC | 3 |  |
| 13 | BCRL:031 | Wang Xuan (2022) | BCRL:160 | BCRL: T: MLD; Co: UC-EX | MLD / UC-EX | 6 |  |
| 14 | BCRL:032 | Ou Qinglian (2023) | BCRL:80 | BCRL: T: MLD; Co: UC-EX | MLD / UC-EX | 3 |  |
| 15 | BCRL:037 | Zhou Jie (2024) | BCRL:70 | BCRL: T: RT; Co: UC-EX | RT / UC-EX | 3 |  |
| 16 | BCRL:038 | Lian Lingling (2016) | BCRL:165 | BCRL: T1: IPC+MLD; T2: MLD; Co: UC-EX | IPC+MLD / MLD / UC-EX | 6 |  |
| 17 | BCRL:039 | Huang Cheng (2021) | BCRL:72 | BCRL: T: AM+IPC; Co: AM | AM+IPC / AM | 3 |  |
| 18 | BCRL:040 | Huang Yuping (2021) | BCRL:86 | BCRL: T: SUP-EX; Co: UC-EX | SUP-EX / UC-EX | 3 |  |
| 19 | BCRL:042 | Wang Hongli (2018) | BCRL:90 | BCRL: T: MLD; Co: UC-EX | MLD / UC-EX | 6 |  |
| 20 | BCRL:043 | Gao Haixia (2014) | BCRL:60 | BCRL: T: IPC; Co: UC-EX | IPC / UC-EX | 3 |  |
| 21 | BCRL:044 | Ma Li (2021) | BCRL:80 | BCRL: T: MLD; Co: UC-EX | MLD / UC-EX | 12 |  |
| 22 | BCRL:047 | Ma Zhiqiang (2018) | BCRL:195 | BCRL: T: IPC+ MW; Co: UC-EX | IPC+MW / UC-EX | 6 |  |
| 23 | BCRL:051 | Hao Suhong (2015) | BCRL:72 | BCRL: T: AM; Co: UC-EX | AM / UC-EX | 3 |  |
| 24 | BCRL:052 | Zhao Yajing (2024) | BCRL:104 | BCRL: T: RT; Co: UC-EX | RT / UC-EX | 6 |  |
| 25 | BCRL:058 | Luo Lingling (2021) | BCRL:110 | BCRL: T: IPC; Co: UC-EX | IPC / UC-EX | 6 |  |
| 26 | BCRL:059 | Cheng Hong (2021) | BCRL:86 | BCRL: T: IPC+RT; Co: UC-EX | IPC+RT / UC-EX | 3 |  |
| 27 | BCRL:061 | Chu Yanqing (2014) | BCRL:80 | BCRL: T: IPC; Co: UC-EX | IPC / UC-EX | 6 |  |
| 28 | BCRL:063 | Shi Lingfang (2022) | BCRL:96 | BCRL: T: OTHERS; Co: UC-EX | OTHERS / UC-EX | 6 |  |
| 29 | BCRL:066 | Wang Shuqing (2020) | BCRL:90 | BCRL: T1: MW; T2: AM+MW; Co: UC-EX | MW / AM+MW / UC-EX | 3 |  |
| 30 | BCRL:067 | Wang Chunying (2019) | BCRL:200 | BCRL: T: MLD; Co: UC-EX | MLD / UC-EX | 6 |  |
| 31 | BCRL:070 | Wang Qianlin (2018) | BCRL:280 | BCRL: T: IPC+MLD; Co: UC-EX | IPC+MLD / UC-EX | 6 |  |
| 32 | BCRL:071 | Mou Qing (2025) | BCRL:90 | BCRL: T: COMP+MLD+RT; Co: MLD | COMP+MLD+RT / MLD | 12 |  |
| 33 | BCRL:072 | Xiong Ping (2017) | BCRL:120 | BCRL: T: IPC+MLD; Co: UC-EX | IPC+MLD / UC-EX | 12 |  |
| 34 | BCRL:073 | Yin Xiuling (2020) | BCRL:126 | BCRL: T1: MLD; T2: MLD+IPC; Co: UC-EX | MLD / IPC+MLD / UC-EX | 9 |  |
| 35 | BCRL:074 | Ouyang Xiangjun (2017) | BCRL:92 | BCRL: T: IPC+MLD; Co: UC-EX | IPC+MLD / UC-EX | 6 |  |
| 36 | BCRL:075 | Liang Xuefang (2020) | BCRL:213 | BCRL: T: IPC+MLD; Co: UC-EX | IPC+MLD / UC-EX | 6 |  |
| 37 | BCRL:076 | Lin Meihong (2021) | BCRL:80 | BCRL: T: IPC; Co: UC-EX | IPC / UC-EX | 6 |  |
| 38 | BCRL:077 | Yang Fuquan (2019) | BCRL:86 | BCRL: T: MW; Co: UC-EX | MW / UC-EX | 12 |  |
| 39 | BCRL:078 | Wu Yuyun (2020) | BCRL:68 | BCRL: T: COMP+RT; Co: UC-EX | COMP+RT / UC-EX | 3 | Volume:includes an extremely sparse node |
| 40 | BCRL:079 | Li He (2015) | BCRL:60 | BCRL: T: RT; Co: UC-EX | RT / UC-EX | 3 | BCRL:double-zero-event study; included in the descriptive network but excluded from the default RR main model |
| 41 | BCRL:080 | Li Xiaojin (2018) | BCRL:116 | BCRL: T: MLD; Co: UC-EX | MLD / UC-EX | 3 |  |
| 42 | BCRL:081 | Li Xiaohua (2021) | BCRL:102 | BCRL: T: COMP+Mox+RT; Co: COMP+RT | COMP+Mox+RT / COMP+RT | 12 |  |
| 43 | BCRL:082 | Li Na (2021) | BCRL:85 | BCRL: T: IPC; Co: UC-EX | IPC / UC-EX | 3 |  |
| 44 | BCRL:083 | Li Jiabao (2019) | BCRL:1472 | BCRL: T: COMP+RT; Co: UC-EX | COMP+RT / UC-EX | 12 |  |
| 45 | BCRL:084 | Zhu Yongcui (2021) | BCRL:60 | BCRL: T: MLD; Co: UC-EX | MLD / UC-EX | 3 |  |
| 46 | BCRL:086 | Xu Xijuan (2020) | BCRL:120 | BCRL: T: MLD; Co: UC-EX | MLD / UC-EX | 6 |  |
| 47 | BCRL:087 | Zhang Yuwei (2014) | BCRL:70 | BCRL: T: MW; Co: UC-EX | MW / UC-EX | 6 |  |
| 48 | BCRL:088 | Zhang Shumin (2025) | BCRL:85 | BCRL: T: COMP+Mox+MLD; Co: COMP+MLD | COMP+Mox+MLD / COMP+MLD | 6 |  |
| 49 | BCRL:091 | Liao Zhen (2015) | BCRL:80 | BCRL: T: COMP; Co: UC-EX | COMP / UC-EX | 3 |  |
| 50 | BCRL:095 | Jiang Xiaoling (2014) | BCRL:90 | BCRL: T: COMP; Co: UC-EX | COMP / UC-EX | 3 |  |
| 51 | BCRL:096 | Yaochen Siyu (2023) | BCRL:109 | BCRL: T1: MLD; T2: COMP+MLD; Co: UC-EX | MLD / COMP+MLD / UC-EX | 3 |  |
| 52 | BCRL:097 | Xia Ying (2018) | BCRL:80 | BCRL: T: IPC; Co: UC-EX | IPC / UC-EX | 3 |  |
| 53 | BCRL:103 | Liu Yu (2016) | BCRL:100 | BCRL: T: Mox; Co: UC-EX | Mox / UC-EX | 3 | Volume:retained outside the window; volume: includes an extremely sparse node |
| 54 | BCRL:106 | Liu Qing (2021) | BCRL:88 | BCRL: T: IPC+MLD; Co: MLD | IPC+MLD / MLD | 3 | ROM:CHECK: record note says abduction ROM difference |
| **No.** | **Study ID** | **Study** | **Quantitative-analysis sample size** | **Original intervention description** | **Node/comparison** | **Time point (months)** | **Notes** |
| 1 | ROM:026 | Xu Lingling (2022) | ROM:170 | ROM: T: AM+IPC; Co: IPC | AM+IPC vs IPC | 3 |  |
| 2 | ROM:028 | Xiao Zhiping (2022) | ROM:64 | ROM: T: ISO+OTHERS; Co: UC-EX | ISO+OTHERS vs UC-EX | 1.17 | ROM:<3 months, supplementary |
| 3 | ROM:034 | Yang Haijun (2021) | ROM:100 | ROM: T: RT; Co: UC-EX | RT vs UC-EX |  | ROM:retained outside the window; volume: retained outside the window; volume: unclear time point; includes an extremely sparse node |
| 4 | ROM:041 | Huang Mei (2022) | ROM:120 | ROM: T: AM+IPC; Co: AM | AM+IPC vs AM | 1 | ROM:<3 months, supplementary |
| 5 | ROM:045 | Ma Xiufen (2013) | ROM:120 | ROM: T: IPC+MLD; Co: UC-EX | IPC+MLD vs UC-EX |  | ROM:retained outside the window |
| 6 | ROM:053 | Jia Jingyi (2023) | ROM:82 | ROM: T: AM+ISO; Co: UC-EX | AM+ISO vs UC-EX | 1 | ROM:<3 months, supplementary |
| 7 | ROM:059 | Cheng Hong (2021) | ROM:86 | ROM: T: IPC+RT; Co: UC-EX | IPC+RT vs UC-EX | 3 |  |
| 8 | ROM:060 | Cheng Yi (2021) | ROM:86 | ROM: T: IPC; Co: UC-EX | IPC vs UC-EX |  | ROM:retained outside the window |
| 9 | ROM:063 | Shi Lingfang (2022) | ROM:96 | ROM: T: OTHERS; Co: UC-EX | OTHERS vs UC-EX | 6 |  |
| 10 | ROM:065 | Wang Qinyu (2021) | ROM:88 | ROM: T: IPC+MLD; Co: MLD | IPC+MLD vs MLD | 1 | ROM:<3 months, supplementary |
| 11 | ROM:068 | Wang Huijing (2012) | ROM:60 | ROM: T: Mox; Co: UC-EX | Mox vs UC-EX | 2 | ROM:<3 months, supplementary |
| 12 | ROM:078 | Wu Yuyun (2020) | ROM:68 | ROM: T: COMP+RT; Co: UC-EX | COMP+RT vs UC-EX | 3 | Volume:includes an extremely sparse node |
| 13 | ROM:082 | Li Na (2021) | ROM:85 | ROM: T: IPC; Co: UC-EX | IPC vs UC-EX | 3 |  |
| 14 | ROM:085 | Xu Youdi (2009) | ROM:100 | ROM: T: IPC; Co: UC-EX | IPC vs UC-EX | 1.17 | ROM:<3 months, supplementary |
| 15 | ROM:097 | Xia Ying (2018) | ROM:80 | ROM: T: IPC; Co: UC-EX | IPC vs UC-EX | 3 |  |
| 16 | ROM:102 | Wu Bing (2022) | ROM:120 | ROM: T: IPC; Co: UC-EX | IPC vs UC-EX | 1 | ROM:<3 months, supplementary |
| 17 | ROM:106 | Liu Qing (2021) | ROM:88 | ROM: T: IPC+MLD; Co: MLD | IPC+MLD vs MLD | 3 | ROM:CHECK: record note says abduction ROM difference |
| 18 | ROM:111 | He Ximei (2025) | ROM:130 | ROM: T: MLD+OTHERS; Co: UC-EX | MLD+OTHERS vs UC-EX | 1 | ROM:<3 months, supplementary |
| **No.** | **Study ID** | **Study** | **Quantitative-analysis sample size** | **Original intervention description** | **Node/comparison** | **Time point (months)** | **Notes** |
| 1 | Volume:001 | Zimmermann (2012) | Volume:67 | Volume: T: MLD; Co: UC-EX | MLD vs UC-EX | 6 | Volume:retained outside the window |
| 2 | Volume:007 | Sagen (2009) | Volume:204 | Volume: T: RT+SUP-EX; Co: SUP-EX | RT+SUP-EX vs SUP-EX |  | Volume:retained outside the window; volume: unclear time point; includes an extremely sparse node |
| 3 | Volume:008 | Ochalek (2018) | Volume:41 | Volume: T: COMP; Co: UC-EX | COMP vs UC-EX | 3 | Volume:retained outside the window; volume: includes an extremely sparse node; converted SD |
| 4 | Volume:010 | Kaş Özdemir (2025) | Volume:42 | Volume: T1: SUP-EX; T2: not used; Co: UC-EX | SUP-EX vs UC-EX | 3 |  |
| 5 | Volume:013 | Devoogdt (2011) | Volume:160 | Volume: T: MLD; Co: UC-EX | MLD vs UC-EX | 12 |  |
| 6 | Volume:014 | Castro-Sanchez (2011) | Volume:48 | Volume: T: COMP+MLD; Co: UC-EX | COMP+MLD vs UC-EX | 8 | Volume:converted SD |
| 7 | Volume:017 | Anderson (2012) | Volume:104 | Volume: T: COMP+SUP-EX; Co: UC-EX | COMP+SUP-EX vs UC-EX | 18 | Volume:retained outside the window; volume: includes an extremely sparse node |
| 8 | Volume:034 | Yang Haijun (2021) | Volume:100 | Volume: T: RT; Co: UC-EX | RT vs UC-EX |  | ROM:retained outside the window; volume: retained outside the window; volume: unclear time point; includes an extremely sparse node |
| 9 | Volume:078 | Wu Yuyun (2020) | Volume:68 | Volume: T: COMP+RT; Co: UC-EX | COMP+RT vs UC-EX | 3 | Volume:includes an extremely sparse node |
| 10 | Volume:086 | Xu Xijuan (2020) | Volume:120 | Volume: T: MLD; Co: UC-EX | MLD vs UC-EX | 6 |  |
| 11 | Volume:100 | Zhao Nixia (2021) | Volume:82 | Volume: T: COMP+MLD+RT; Co: COMP+MLD | COMP+MLD+RT vs COMP+MLD | 3 | Volume:includes an extremely sparse node |
| 12 | Volume:103 | Liu Yu (2016) | Volume:100 | Volume: T: Mox; Co: UC-EXT: Mox; Co: UC-EX | Mox vs UC-EX | 1.87 | Volume:retained outside the window; volume: includes an extremely sparse node |
| **No.** | **Study** | **Disposition** |  |  |  |  |  |
| 1 | Qiu Xiaoxi (2018) | Retained in Supplementary Table S4-1 as a full-text included/qualitatively described study; not included in the final three-outcome Bayesian NMA quantitative synthesis set listed in Supplementary Table S4-2. This table only indicates study disposition and should not be interpreted as a study-by-study exclusion reason or a formal node-mapping basis. |  |  |  |  |  |
| 2 | Wan Hui (2019) | Retained in Supplementary Table S4-1 as a full-text included/qualitatively described study; not included in the final three-outcome Bayesian NMA quantitative synthesis set listed in Supplementary Table S4-2. This table only indicates study disposition and should not be interpreted as a study-by-study exclusion reason or a formal node-mapping basis. |  |  |  |  |  |
| 3 | Zhang (2025) | Retained in Supplementary Table S4-1 as a full-text included/qualitatively described study; not included in the final three-outcome Bayesian NMA quantitative synthesis set listed in Supplementary Table S4-2. This table only indicates study disposition and should not be interpreted as a study-by-study exclusion reason or a formal node-mapping basis. |  |  |  |  |  |
| 4 | Donmez (2017) | Retained in Supplementary Table S4-1 as a full-text included/qualitatively described study; not included in the final three-outcome Bayesian NMA quantitative synthesis set listed in Supplementary Table S4-2. This table only indicates study disposition and should not be interpreted as a study-by-study exclusion reason or a formal node-mapping basis. |  |  |  |  |  |
| 5 | Ridner (2019) | Retained in Supplementary Table S4-1 as a full-text included/qualitatively described study; not included in the final three-outcome Bayesian NMA quantitative synthesis set listed in Supplementary Table S4-2. This table only indicates study disposition and should not be interpreted as a study-by-study exclusion reason or a formal node-mapping basis. |  |  |  |  |  |
| 6 | Bruce (2022) | Retained in Supplementary Table S4-1 as a full-text included/qualitatively described study; not included in the final three-outcome Bayesian NMA quantitative synthesis set listed in Supplementary Table S4-2. This table only indicates study disposition and should not be interpreted as a study-by-study exclusion reason or a formal node-mapping basis. |  |  |  |  |  |
| 7 | Wang Xianqing (2016) | Retained in Supplementary Table S4-1 as a full-text included/qualitatively described study; not included in the final three-outcome Bayesian NMA quantitative synthesis set listed in Supplementary Table S4-2. This table only indicates study disposition and should not be interpreted as a study-by-study exclusion reason or a formal node-mapping basis. |  |  |  |  |  |
| 8 | Lin Caijuan (2011) | Retained in Supplementary Table S4-1 as a full-text included/qualitatively described study; not included in the final three-outcome Bayesian NMA quantitative synthesis set listed in Supplementary Table S4-2. This table only indicates study disposition and should not be interpreted as a study-by-study exclusion reason or a formal node-mapping basis. |  |  |  |  |  |
| 9 | Zhang Yin (2015) | Retained in Supplementary Table S4-1 as a full-text included/qualitatively described study; not included in the final three-outcome Bayesian NMA quantitative synthesis set listed in Supplementary Table S4-2. This table only indicates study disposition and should not be interpreted as a study-by-study exclusion reason or a formal node-mapping basis. |  |  |  |  |  |
| 10 | Zhuang Yuhong (2025) | Retained in Supplementary Table S4-1 as a full-text included/qualitatively described study; not included in the final three-outcome Bayesian NMA quantitative synthesis set listed in Supplementary Table S4-2. This table only indicates study disposition and should not be interpreted as a study-by-study exclusion reason or a formal node-mapping basis. |  |  |  |  |  |
| 11 | Ma Yuping (2024) | Retained in Supplementary Table S4-1 as a full-text included/qualitatively described study; not included in the final three-outcome Bayesian NMA quantitative synthesis set listed in Supplementary Table S4-2. This table only indicates study disposition and should not be interpreted as a study-by-study exclusion reason or a formal node-mapping basis. |  |  |  |  |  |
| 12 | Gu Yetong (2020) | Retained in Supplementary Table S4-1 as a full-text included/qualitatively described study; not included in the final three-outcome Bayesian NMA quantitative synthesis set listed in Supplementary Table S4-2. This table only indicates study disposition and should not be interpreted as a study-by-study exclusion reason or a formal node-mapping basis. |  |  |  |  |  |
| 13 | Chen Li (2018) | Retained in Supplementary Table S4-1 as a full-text included/qualitatively described study; not included in the final three-outcome Bayesian NMA quantitative synthesis set listed in Supplementary Table S4-2. This table only indicates study disposition and should not be interpreted as a study-by-study exclusion reason or a formal node-mapping basis. |  |  |  |  |  |
| 14 | Chen Yuqin (2022) | Retained in Supplementary Table S4-1 as a full-text included/qualitatively described study; not included in the final three-outcome Bayesian NMA quantitative synthesis set listed in Supplementary Table S4-2. This table only indicates study disposition and should not be interpreted as a study-by-study exclusion reason or a formal node-mapping basis. |  |  |  |  |  |
| 15 | Yuan Yulian (2023) | Retained in Supplementary Table S4-1 as a full-text included/qualitatively described study; not included in the final three-outcome Bayesian NMA quantitative synthesis set listed in Supplementary Table S4-2. This table only indicates study disposition and should not be interpreted as a study-by-study exclusion reason or a formal node-mapping basis. |  |  |  |  |  |
| 16 | Jiang Jinying (2025) | Retained in Supplementary Table S4-1 as a full-text included/qualitatively described study; not included in the final three-outcome Bayesian NMA quantitative synthesis set listed in Supplementary Table S4-2. This table only indicates study disposition and should not be interpreted as a study-by-study exclusion reason or a formal node-mapping basis. |  |  |  |  |  |
| 17 | Dong Yan (2017) | Retained in Supplementary Table S4-1 as a full-text included/qualitatively described study; not included in the final three-outcome Bayesian NMA quantitative synthesis set listed in Supplementary Table S4-2. This table only indicates study disposition and should not be interpreted as a study-by-study exclusion reason or a formal node-mapping basis. |  |  |  |  |  |
| 18 | Mao Chuanlan (2020) | Retained in Supplementary Table S4-1 as a full-text included/qualitatively described study; not included in the final three-outcome Bayesian NMA quantitative synthesis set listed in Supplementary Table S4-2. This table only indicates study disposition and should not be interpreted as a study-by-study exclusion reason or a formal node-mapping basis. |  |  |  |  |  |
| 19 | Zhu Yanan (2017) | Retained in Supplementary Table S4-1 as a full-text included/qualitatively described study; not included in the final three-outcome Bayesian NMA quantitative synthesis set listed in Supplementary Table S4-2. This table only indicates study disposition and should not be interpreted as a study-by-study exclusion reason or a formal node-mapping basis. |  |  |  |  |  |
| 20 | Wang Hong (2018) | Retained in Supplementary Table S4-1 as a full-text included/qualitatively described study; not included in the final three-outcome Bayesian NMA quantitative synthesis set listed in Supplementary Table S4-2. This table only indicates study disposition and should not be interpreted as a study-by-study exclusion reason or a formal node-mapping basis. |  |  |  |  |  |
| 21 | Wang Shuai (2011) | Retained in Supplementary Table S4-1 as a full-text included/qualitatively described study; not included in the final three-outcome Bayesian NMA quantitative synthesis set listed in Supplementary Table S4-2. This table only indicates study disposition and should not be interpreted as a study-by-study exclusion reason or a formal node-mapping basis. |  |  |  |  |  |
| 22 | Zuo Yaqin (2014) | Retained in Supplementary Table S4-1 as a full-text included/qualitatively described study; not included in the final three-outcome Bayesian NMA quantitative synthesis set listed in Supplementary Table S4-2. This table only indicates study disposition and should not be interpreted as a study-by-study exclusion reason or a formal node-mapping basis. |  |  |  |  |  |
| 23 | Song Lifang (2024) | Retained in Supplementary Table S4-1 as a full-text included/qualitatively described study; not included in the final three-outcome Bayesian NMA quantitative synthesis set listed in Supplementary Table S4-2. This table only indicates study disposition and should not be interpreted as a study-by-study exclusion reason or a formal node-mapping basis. |  |  |  |  |  |
| 24 | Sun Lianqin (2009) | Retained in Supplementary Table S4-1 as a full-text included/qualitatively described study; not included in the final three-outcome Bayesian NMA quantitative synthesis set listed in Supplementary Table S4-2. This table only indicates study disposition and should not be interpreted as a study-by-study exclusion reason or a formal node-mapping basis. |  |  |  |  |  |
| 25 | Zhou Hong (2013) | Retained in Supplementary Table S4-1 as a full-text included/qualitatively described study; not included in the final three-outcome Bayesian NMA quantitative synthesis set listed in Supplementary Table S4-2. This table only indicates study disposition and should not be interpreted as a study-by-study exclusion reason or a formal node-mapping basis. |  |  |  |  |  |
| 26 | Wu Jing (2017) | Retained in Supplementary Table S4-1 as a full-text included/qualitatively described study; not included in the final three-outcome Bayesian NMA quantitative synthesis set listed in Supplementary Table S4-2. This table only indicates study disposition and should not be interpreted as a study-by-study exclusion reason or a formal node-mapping basis. |  |  |  |  |  |
| 27 | Wu Yuping (2015) | Retained in Supplementary Table S4-1 as a full-text included/qualitatively described study; not included in the final three-outcome Bayesian NMA quantitative synthesis set listed in Supplementary Table S4-2. This table only indicates study disposition and should not be interpreted as a study-by-study exclusion reason or a formal node-mapping basis. |  |  |  |  |  |
| 28 | Liu Chunling (2020) | Retained in Supplementary Table S4-1 as a full-text included/qualitatively described study; not included in the final three-outcome Bayesian NMA quantitative synthesis set listed in Supplementary Table S4-2. This table only indicates study disposition and should not be interpreted as a study-by-study exclusion reason or a formal node-mapping basis. |  |  |  |  |  |
| 29 | Liu Pinqian (2019) | Retained in Supplementary Table S4-1 as a full-text included/qualitatively described study; not included in the final three-outcome Bayesian NMA quantitative synthesis set listed in Supplementary Table S4-2. This table only indicates study disposition and should not be interpreted as a study-by-study exclusion reason or a formal node-mapping basis. |  |  |  |  |  |
| 30 | Liu Huayu (2013) | Retained in Supplementary Table S4-1 as a full-text included/qualitatively described study; not included in the final three-outcome Bayesian NMA quantitative synthesis set listed in Supplementary Table S4-2. This table only indicates study disposition and should not be interpreted as a study-by-study exclusion reason or a formal node-mapping basis. |  |  |  |  |  |
| 31 | He Yuanmei (2021) | Retained in Supplementary Table S4-1 as a full-text included/qualitatively described study; not included in the final three-outcome Bayesian NMA quantitative synthesis set listed in Supplementary Table S4-2. This table only indicates study disposition and should not be interpreted as a study-by-study exclusion reason or a formal node-mapping basis. |  |  |  |  |  |

**Supplementary Material S5. BCRL league table (11-node primary analysis)**

See standalone file: **S5_BCRL_League_Table.xlsx**

Complete 11 × 11 pairwise comparison league table for BCRL incidence (27 studies, 11 treatment nodes, ≥6-month follow-up). Each cell shows RR (95% CrI). Significant comparisons highlighted.

**Supplementary Material S6. BCRL ranking probabilities (11-node primary analysis)**

See standalone file: **S6_Ranking_Probabilities.xlsx**

Ranking probabilities for 11 treatment nodes (ranks 1–11), SUCRA values, and overall ranks. Cumulative ranking curves: BCRL_Cumulative_Ranking_Curves.png/pdf.

**Supplementary Material S7. BCRL model convergence and fit diagnostics**

See standalone file: **S7_BCRL_Convergence.xlsx**

DIC, Rhat, ESS, τ estimates for the BCRL primary analysis. Gelman–Rubin plots and MCMC trace/density plots provided as separate figure files.

**Supplementary Material S8. BCRL dataset sensitivity analysis**

See standalone file: **S8_BCRL_Dataset_Sensitivity.xlsx**

Five dataset sensitivity analyses (primary, sparse-removed, volumetric-only, clear-definition, non-Chinese) with within-subset sparse-node removal. Note: COMP+RT excluded as sparse in volumetric and clear-definition subsets. Summary of DIC, τ, Rhat, ESS, and top-ranked treatments.

**Supplementary Material S9. BCRL prior sensitivity analysis (11-node)**

See standalone file: **S9_S11_Prior_Sensitivity.xlsx (Sheet 1: BCRL)**

Baseline: d ~ N(0, 2.5²), τ ~ HN(0, 1²). Five alternatives tested. COMP+RT rank 1 in all (SUCRA 84.9%–88.2%).

**Supplementary Material S10. BCRL consistency assessment (11-node)**

See standalone file: **S10_BCRL_Consistency.xlsx**

DIC comparison: ΔDIC = 0 (no inconsistency). Node-splitting: IPC+MLD vs MLD, p = 0.264.

**Supplementary Material S11. Complete Bayesian NMA results for ROM (11-node)**

See standalone file: **S11_ROM_League_Table.xlsx + S11_ROM_Ranking_Probabilities.xlsx + S9_S11_Prior_Sensitivity.xlsx (Sheet 2)**

ROM league table (MD, 95% CrI), ranking probabilities, SUCRA, prior sensitivity (AM+ISO rank 1 in all), consistency (ΔDIC = 0.04). Egger p = 0.037. Curves: ROM_Cumulative_Ranking_Curves_1.png/pdf.

**Supplementary Material S12. Bayesian NMA results for absolute volume difference (descriptive)**

Note: NMA for this outcome was abandoned in the main analysis due to irreconcilable data quality issues (see Section 3.6). These exploratory results are provided for transparency only and were not used for inference, ranking, or integrated interpretation.

**Supplementary Table S12-1. Volume difference MD results**

| **Intervention node** | **MD vs UC-EX** | **95% CrI** | **mean (supplementary)** | **Parameter** |
| --- | --- | --- | --- | --- |
| COMP | -22.20 | -254.43–215.56 | -21.6217544675207 | d.UC_EX.COMP |
| COMP+MLD | -190.63 | -496.34–119.08 | -189.969483350128 | d.UC_EX.COMP_MLD |
| COMP+MLD+RT | -341.75 | -723.53–35.21 | -341.474273773219 | d.UC_EX.COMP_MLD_RT |
| COMP+RT | -156.80 | -382.51–65.63 | -156.648628162155 | d.UC_EX.COMP_RT |
| COMP+SUP-EX | -29.33 | -263.09–204.41 | -28.8660428410591 | d.UC_EX.COMP_SUP_EX |
| MLD | -114.58 | -248.63–19.55 | -114.344589173134 | d.UC_EX.MLD |
| Mox | -80.82 | -305.16–142.99 | -80.5772486777595 | d.UC_EX.Mox |
| RT | -106.99 | -332.48–121.03 | -106.639235689147 | d.UC_EX.RT |
| RT+SUP-EX | -4.31 | -332.33–326.71 | -4.85298074053141 | d.UC_EX.RT_SUP_EX |
| SUP-EX | -36.46 | -272.76–197.44 | -36.4629607448873 | d.UC_EX.SUP_EX |
| treatment | rank_1 | rank_2 | rank_3 | rank_4 |
| COMP | 0.01315 | 0.02555 | 0.045225 | 0.061775 |
| COMP_MLD | 0.0228 | 0.410625 | 0.14495 | 0.0941 |
| COMP_MLD_RT | 0.713225 | 0.105125 | 0.050675 | 0.03505 |
| COMP_RT | 0.10235 | 0.166725 | 0.228075 | 0.147425 |
| COMP_SUP_EX | 0.0158 | 0.027375 | 0.050325 | 0.065775 |
| MLD | 0.01925 | 0.06295 | 0.140775 | 0.205825 |
| Mox | 0.029875 | 0.054875 | 0.0983 | 0.1177 |
| RT | 0.045225 | 0.0831 | 0.1396 | 0.1459 |
| RT_SUP_EX | 0.027125 | 0.03595 | 0.050925 | 0.0535 |
| SUP_EX | 0.0112 | 0.027725 | 0.05065 | 0.07015 |
| UC_EX | 0.0 | 0.0 | 0.0005 | 0.0028 |
| Model/analysis | DIC | Maximum upper Rhat | Minimum ESS |  |
| inconsistency_UME_HN100 |  |  | 26772.2 |  |
| main_random_HN100 |  |  | 26703.7 |  |
| sensitivity_tau_HN250 |  |  | 16614.6 |  |
| sensitivity_tau_HN50 |  |  | 34163.5 |  |
| sensitivity_tau_UNIF500 |  |  | 16085.9 |  |

**Supplementary Material S13. CINeMA assessment (11-node primary analyses)**

See standalone file: **S13_CINeMA_11node_BCRL_ROM.xlsx**

Four sheets: (1) BCRL CINeMA: 55 comparisons × 6 domains, Moderate 12, Low 10, Very low 33. (2) ROM CINeMA: 55 comparisons, Low 10, Very low 45. (3) Distribution summary. (4) Assessment notes.

**Supplementary Material S14. Contribution matrix**

See standalone file: **S14_Contribution_Matrix.xlsx**

BCRL 11-node contribution matrix. Heatmap: A6_contribution_heatmap.png.

**Supplementary Material S15. Summary of supplementary figures**

| **Figure** | **Description** | **File** |
| --- | --- | --- |
| Figure 1 | PRISMA flow diagram | PRISMA_2020_FlowDiagram_确定版_drawio.png/pdf |
| Figure 2 | RoB 2 summary barplot | RoB2_summary_barplot.png/pdf |
| Fig S2-1 | RoB 2 traffic light | RoB2_traffic_light.png/pdf |
| Fig S5-1 | BCRL network plot | A5_network_plot.png |
| Fig S5-2 | BCRL funnel plot | A5_funnel_plot.png |
| Fig S6-1 | BCRL cumulative ranking curves | BCRL_Cumulative_Ranking_Curves.png/pdf |
| Fig S6-2 | BCRL SUCRA barplot | A2_SUCRA_barplot.png |
| Fig S11-1 | ROM network plot | C3_network_plot.png |
| Fig S11-2 | ROM cumulative ranking curves | ROM_Cumulative_Ranking_Curves_1.png/pdf |
| Fig S11-3 | ROM SUCRA barplot | C1_SUCRA_barplot.png |
| Fig S14-1 | Contribution heatmap | A6_contribution_heatmap.png |
